# The *Cordyceps* Genus as a Potential Source of Bioactive Compounds for Adjuvant Cancer Therapy: A Network Pharmacology Approach

**DOI:** 10.3390/ph18050667

**Published:** 2025-04-30

**Authors:** Jose Luis Gonzalez-Llerena, Daniela Treviño-Almaguer, Jesus Alejandro Leal-Mendez, Gael Garcia-Valdez, Arely Guadalupe Balderas-Moreno, Michel Stéphane Heya, Isaias Balderas-Renteria, María del Rayo Camacho-Corona, Bryan Alejandro Espinosa-Rodriguez

**Affiliations:** 1 Laboratory of Molecular Pharmacology and Biological Models, School of Chemistry, Universidad Autonoma de Nuevo Leon, Monterrey 64570, Nuevo Leon, Mexico; jgonzalezl@uanl.edu.mx (J.L.G.-L.); daniela.trevinoal@uanl.edu.mx (D.T.-A.); jesus.lealm@uanl.edu.mx (J.A.L.-M.); gael.garciavld@uanl.edu.mx (G.G.-V.); guadalupe.balderasmrn@uanl.edu.mx (A.G.B.-M.); isaias.balderasrn@uanl.edu.mx (I.B.-R.); 2 Faculty of Public Health and Nutrition, Universidad Autonoma de Nuevo Leon, Monterrey 64460, Nuevo Leon, Mexico; michel.heyax@uanl.edu.mx; 3 Laboratory of Pharmaceutical Chemistry, School of Chemistry, Universidad Autonoma de Nuevo Leon, Monterrey 64570, Nuevo Leon, Mexico; 4 Laboratory of Biopharmacy, School of Chemistry, Universidad Autonoma de Nuevo Leon, San Nicolas de los Garza 66451, Nuevo Leon, Mexico

**Keywords:** *Cordyceps*, network pharmacology, cancer therapeutics, molecular docking, metabolite profiling, drug resistance, multitarget agents, natural products

## Abstract

**Background/Objectives:** Cancer remains one of the leading causes of mortality globally, underscoring the need for novel therapeutic strategies capable of targeting multiple molecular pathways simultaneously. Natural products, particularly fungal-derived metabolites from the genus *Cordyceps*, represent promising candidates due to their diverse biological activities. Although previous studies have indicated the anticancer potential of *Cordyceps* species, systematic characterization of their molecular targets has been limited. This study aimed to comprehensively identify and evaluate *Cordyceps* metabolites as potential multitarget anticancer agents through a network pharmacology approach. **Methods:** A total of 129 metabolites previously reported in the literature from polar aqueous, alcoholic, and non-polar extracts of *Cordyceps* were compiled and chemically classified using ChemMine tools. Structure-based target prediction and pathway enrichment analyses were performed to investigate their potential biological targets. Predicted molecular targets were cross-referenced with differentially expressed genes in breast, colorectal, and lung cancers to identify hub proteins. Molecular docking simulations were conducted to assess binding affinities of metabolites to key oncogenic targets, and SwissADME was utilized for pharmacokinetic profiling. **Results:** The analysis revealed that *Cordyceps* metabolites targeted critical oncogenic pathways, including cell cycle regulation, DNA replication, and apoptosis. Hub proteins such as TYMS, AURKA, and CDK1 were identified as primary targets. Docking simulations highlighted metabolites such as cordycepsidone A, jiangxienone, and flazin, demonstrating binding affinities comparable or superior to clinically used inhibitors. Pharmacokinetic profiling identified several metabolites with favorable drug-like properties, supporting their potential as lead compounds. **Conclusions:** *Cordyceps* extracts contain structurally diverse metabolites capable of modulating multiple cancer-relevant molecular targets, providing a robust foundation for their development into multitarget anticancer therapies. This integrative network pharmacology approach underscores the potential of fungal metabolites in oncology drug discovery.

## 1. Introduction

Cancer is a collection of diseases characterized by an uncontrolled proliferation of cells because of improper regulation in the mechanisms that control proliferative signaling, cell cycle regulators, programmed cell death, and growth regulators [1]. These characteristics lead to the development of tumors that acquire new blood vessels, the capacity to invade nearby tissues, and the potential to migrate to distant organs in the body. In recent years, the incidence and mortality of cancer have been increasing despite the new advances in its understanding and the development of new modalities for its management. For instance, in 2022, the International Agency for Research on Cancer (IARC) reported almost 20 million new cases of cancer, and lung, breast, and colorectal tumors showed the highest incidence. The same year, IARC reported 9.7 million deaths attributed to cancer, and the same tumor types were among those with the highest mortality [2]. Currently, the National Cancer Institute describes nine main treatments for the different types of cancer, including hormone therapy, radiotherapy, surgery, immunotherapy, hyperthermia, and chemotherapy, among others [3]. According to the clinical guidelines reported by the National Comprehensive Cancer Network (NCCN), chemotherapy still represents an essential component in the management of cancer [4]. However, its efficacy is limited by the development of short- and long-term adverse effects [5]. This situation results in a constant search of new potential candidate drugs that may overcome the current antineoplastic drugs in efficacy and safety.

The development of antineoplastic drugs has been changing dramatically with the progress in the understanding of cancer biology [6]. For instance, the concept of hallmarks of cancer described by Hanahan and Weinberg has strengthened the study of cancer and helped to understand key malignant transformation processes. In 2000, they defined six essential traits, including sustained proliferation, evasion of growth suppressors, resistance to apoptosis, unlimited replication, angiogenesis, and metastatic capacity [7]. In 2011, they added immune evasion, dysregulation of energy metabolism, and two facilitating conditions: genomic instability and tumor-promoting inflammation [1]. This study model of cancer has enabled the development of targeted therapies using tyrosine kinase inhibitors, apoptosis inducers, antiangiogenic agents, and immunotherapies, improving survival in various types of cancer [8]. However, intratumoral heterogeneity and the remarkable phenotypic adaptability of malignant cells require more integrated and synergistic therapeutic strategies to avoid resistance.

In addition, the concept of cellular plasticity has emerged as a new hallmark, as it allows cancer cells to transition between epithelial and mesenchymal states, with the ability to reprogram themselves toward alternative lineages, increasing their therapeutic evasion [9]. Similarly, cellular senescence, traditionally considered an antitumor barrier due to its ability to halt cell proliferation, has been re-evaluated in its relationship with cancer, as evidence shows that the persistence of senescent cells, and their secretion of proinflammatory factors known as ‘senescence-associated secretory phenotype’ (SASP) can promote tumor progression and recurrence after conventional treatments [10]. To combat this, dual strategies have been developed that combine the controlled induction of senescence with the subsequent elimination of senescent cells using senolytic agents [11]. 

Another key therapeutic focus is the complexity of the tumor microenvironment (TME), composed of immune cells, fibroblasts, extracellular matrix, and a network of cytokines [12]. TME modulates tumor progression and influences the efficacy of therapies. Immunotherapy, through immunoblockade, has transformed inactive microenvironments into immunologically active ones, leading to substantial improvements in survival in patients with melanoma and other malignancies [13]. 

The identification of new molecules that may modulate the hallmarks of cancer, TME, and the program of senescence are active areas of research in medicinal chemistry, and natural products represent a common source for new drug discoveries. Indeed, many chemotherapeutic drugs still recommended by the NCCN, such as paclitaxel, doxorubicin, etoposide, and irinotecan, were isolated or derived from nature, including plants, animals, and fungi [14]. In the current landscape of biomedical research, fungi have emerged as a promising source of bioactive compounds and this is largely due to their ability to synthesize secondary metabolites with complex chemical structures and diverse pharmacological properties [15]. Within this context, the *Cordyceps* genus, a collection of ascomycete parasitic fungi that feed on insects and arthropods, has gained great relevance in recent years because of its promising activity over a wide range of diseases, including type 2 diabetes mellitus, autoimmune disorders, aging, and cancer [16].

Among its most notable bioactive constituents, cordycepin has demonstrated the ability to induce apoptosis and regulate key signaling pathways, including PI3K-Akt-mTOR, and alter the transcription of critical genes involved in essential cellular processes, including cell cycle regulation, mitotic control, DNA replication and genomic integrity, centrosome dynamics, and mechanisms underlying chemotherapeutic resistance [17]. Additionally, more than 20 compounds have been identified in *Cordyceps*, including nucleosides, polysaccharides, sterols, and lactones, different peptides like cordycedipeptide A, and cordymin [16]. Furthermore, the discovery of ergosterol, present in some *Cordyceps* extracts, as a cytotoxic agent shows that even seemingly common metabolites can reveal high therapeutic potential upon closer examination or structural modification [18].

In recent years, new methodologies have arisen to design new therapeutic combinations that prevent resistance emergence, enhance therapeutic synergies, and discover new therapeutic molecules. These approaches combine omics data, computational simulations, and experimental validations. The integration of this knowledge applied to the field of pharmacology is known as ‘network pharmacology’, an approach that moves away from traditional paradigm and embraces the complexity of molecular interactions [19]. 

In this framework, our study proposes adopting a network pharmacology approach to identify and predict potential therapeutic targets of bioactive metabolites present in *Cordyceps* extracts. We seek to create a functional map that illustrates how these molecules modulate key pathways in cancer progression through the integration of chemical data, computational prediction of targets, and analysis of molecular interactions. This approach, in addition to validating the antitumor potential of *Cordyceps* from a mechanistic perspective, lays the groundwork for the rational development of more effective multitarget therapies that are less prone to tumor resistance. Finally, the proposed strategy highlights the use of natural compounds with pharmacological potential in cancer, as well as the exploration of therapeutic synergies, enabling the design of safer and more personalized adjuvant therapies that enhance the clinical value of fungi-derived medicinal products in oncology. 

## 2. Results

### 2.1. Chemical Classification of Cordyceps Metabolites

Metabolites previously reported in polar aqueous, alcoholic, and non-polar extracts of *Cordyceps* were compiled from existing literature sources [20,21,22,23]. In the polar aqueous extract, 19 metabolites were included, such as adenosine, 3′-deoxyinosine, adenine, guanine, ergothioneine, and 2′-deoxyuridine, among others. The polar alcoholic extract was composed of 63 metabolites that included cordycepin, daidzein, annullatin A, cordycepol, ophicordin, and cordysinin A, among others. On the other hand, the non-polar extract was composed of 47 metabolites, including pinophilin C, cordycol, terreusinone A, rugulosin, skyrin, cordypyridone A, ergosterol, and palmitic acid, among others. These metabolites were clustered in ChemMine tools to identify the most representative families of natural products in each extract.

The polar aqueous extract was composed mainly of five groups: purines, nucleosides, cytosine, thymine, and ergothioneine. The cluster that contained more metabolites was the one that was composed of nucleosides. Although cytosine and thymine are both pyrimidines, they were not clustered, probably because they differ in their methylation status. Ergothioneine was excluded as a separate cluster because it is not a nucleoside nor a nitrogenous base. In addition, some metabolites agreed well with the expected metabolites present in the aqueous fraction based on their physicochemical properties because nucleosides and ergothioneine are soluble in water, while purines and pyrimidines tend to be poorly soluble in water. For the polar alcoholic extract, the chemical composition consisted in 28 clusters, including cordycepiamides, peptides, cordycepin and derivatives, annullatins, and derivatives of erythrostominone as the clusters with the highest number of members. The non-polar extract was composed of 29 clusters, and the top five most prominent clusters were fatty acids, derivatives of ergosterol, cordypyridones, annullatins, and derivatives of erythrostominone, among others, while the rest of the clusters were composed of one or two members. On the other hand, the pattern of metabolites expected in the polar alcoholic and the non-polar fractions agreed well with those reported in the literature. For instance, annullatins tend to show poor solubility in water but a high solubility in alcohols and a moderate solubility in non-polar solvents. Indeed, annullatins were present in both the alcoholic and the non-polar fractions. Other examples were the fatty acids and the derivatives of ergosterol that appeared only in the non-polar extract due to their high lipophilicity.

### 2.2. Predicted Targets of Cordyceps-Derived Metabolites 

**Figure 1:**
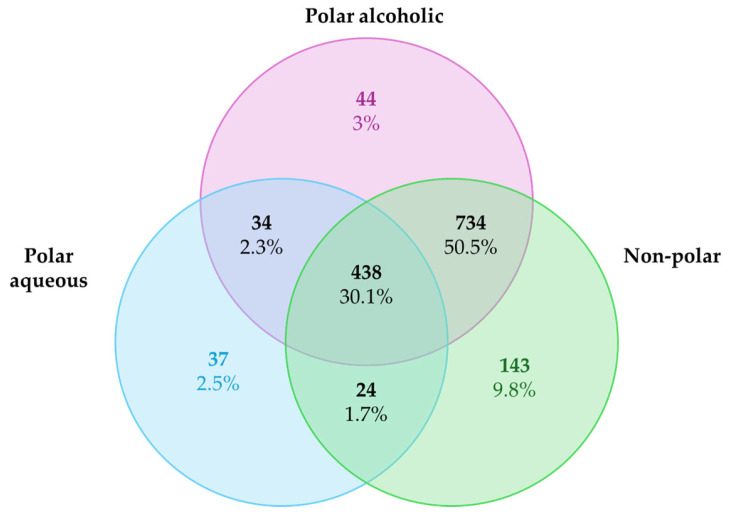
Venn diagram of the target predicted for the polar aqueous, polar alcoholic, and non-polar fraction.

The list of predicted targets, their corresponding common names, Uniprot IDs, ChEMBL IDs, target class, probability of the prediction, and known actives (3D/2D) for each metabolite were grouped into three different list according to the extract categories and deleting duplicates. The targets collected were 533 for the polar aqueous fraction, 1250 for the polar alcoholic fraction, and 1339 for the non-polar fraction (Figure 1). It is noteworthy that 30.1% of the predicted targets (n = 438) were shared across all extract types. A larger proportion, 59.5% (n = 734), was common to the polar alcoholic and non-polar fractions, while only 2.3% (n = 34) overlapped between the polar aqueous and alcoholic fractions, and 1.7% (n = 24) were shared by the polar aqueous and non-polar fractions. The complete lists of predicted targets for each extract can be found in the Supplementary Materials.

The list of predicted targets was analyzed in ShinyGO 0.82 to see which pathways were altered by selecting 20 representative pathways with an FDR cutoff of 0.05. We obtained eight shared pathways between the three extracts (30.1%): metabolic pathways, lipid and atherosclerosis, microRNAs in cancer, hepatitis B, PI3K-Akt signaling pathway, neuroactive ligand-receptor interaction, pathways in cancer, and proteoglycans in cancer. Also, the non-polar and polar alcoholic fraction (59.5%) shared seven pathways such as pathways of neurodegeneration-multiple diseases, calcium signaling pathway, Alzheimer disease, MAPK signaling pathway, spinocerebellar ataxia, cAMP signaling pathway, and Epstein–Barr virus infection. Among the polar fractions (2.3%) we found three common pathways: EGFR tyrosine kinase inhibitor resistance, Kaposi sarcoma-associated herpesvirus infection, and apoptosis. Finally, polar aqueous and non-polar fraction (1.7%) shared two pathways: prostate cancer and FoxO signaling pathway. 

### 2.3. Cancer-Associated Targets Affected by Metabolites from Cordyceps Extracts 

**Figure 2:**
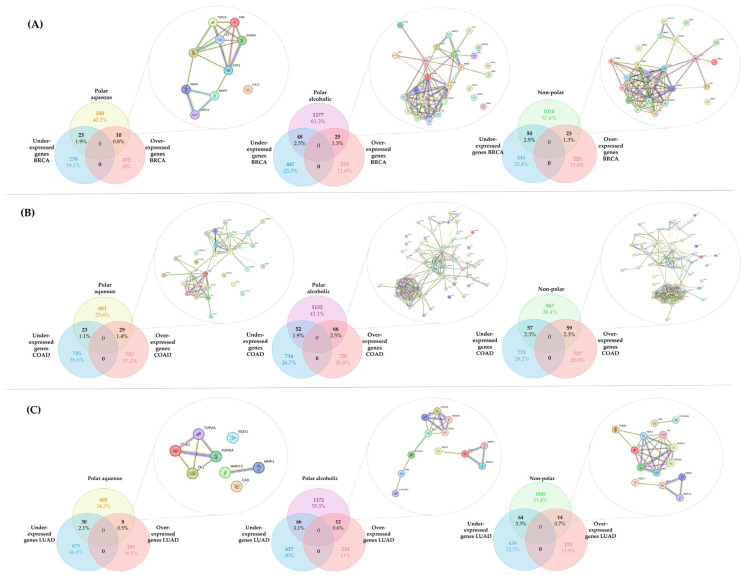
Venn diagram of the overlapping proteins between the differential genes (over- and underexpressed) of (**A**) BRCA, (**B**) COAD, and (**C**) LUAD and the predicted targets of each extract. Only the PPI network corresponding to the overexpressed genes is shown. This figure was generated using individual images of the PPI network obtained from STRING.

Once the lists of predicted targets were obtained for each *Cordyceps* extract (polar aqueous, polar alcoholic, and non-polar), they were individually compared using Venn diagrams, with the lists of differentially overexpressed (Log_2_FC ≥ 2) and underexpressed (Log_2_FC ≤ −2) genes identified in breast invasive carcinoma (BRCA), colon adenocarcinoma (COAD), and lung adenocarcinoma (LUAD), using standardized cancer-type abbreviations retrieved from the GEPIA2 platform (Gene Expression Profiling Interactive Analysis), which are based on TCGA and GTEx datasets. The common targets were analyzed through protein–protein interaction (PPI) network analysis using the STRING database. Figure 2 illustrates the overlap between the differentially expressed genes (both over- and underexpressed) and the predicted targets of each extract; however, only the PPI network corresponding to the overexpressed genes is shown. For complete PPI data, including underexpressed genes, refer to the Supplementary Materials. The PPI networks were sent to Cytoscape to obtain the hub proteins by calculating the nodes and ranking a top three using maximal clique centrality (MCC) as the centrality measure. Table 1 shows the hub proteins identified by Cytoscape 3.10.3.

**Figure 3:**
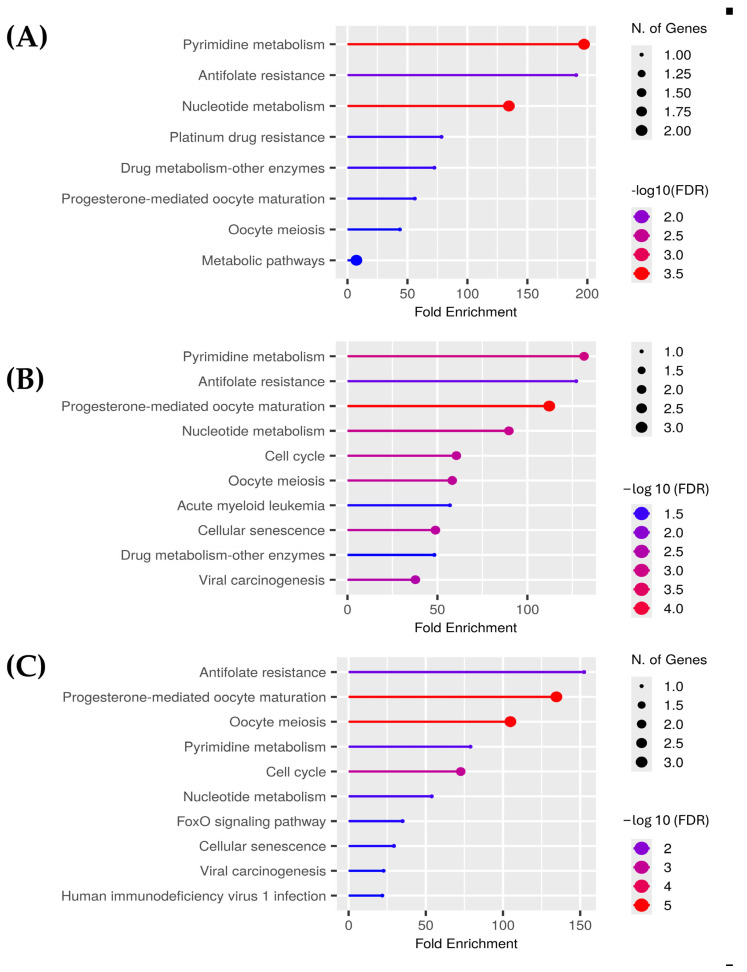
Enriched pathways associated with the top three hub proteins derived from the overexpressed genes of breast invasive carcinoma (BRCA), colon adenocarcinoma (COAD), and lung adenocarcinoma (LUAD), collectively altered by the (**A**) polar aqueous, (**B**) polar alcoholic, and (**C**) non-polar fractions of *Cordyceps*. Pathway enrichment was performed using ShinyGO 0.82 with an FDR cutoff of 0.05. This figure was generated using individual images obtained from ShinyGO 0.82.

**Figure 4:**
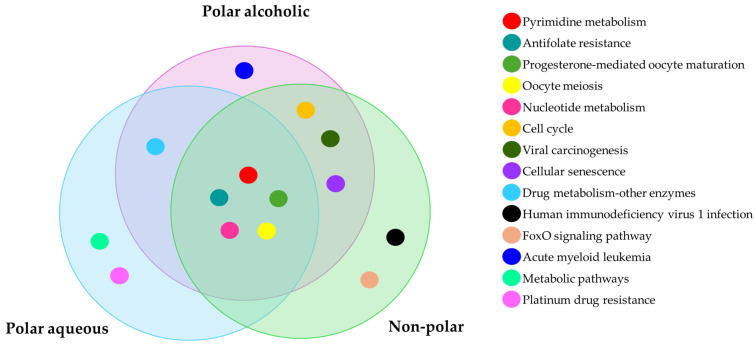
Venn diagram of the overlapping enriched pathways of the top three hub proteins of BRCA, COAD, and LUAD together altered by the polar aqueous, polar alcoholic, and non-polar fraction of *Cordyceps* obtained from ShinyGO 0.82 with an FDR cutoff of 0.05.

For the polar aqueous fraction, the hub proteins obtained by evaluating the overexpressed genes in the three types of cancer (BRCA, COAD, and LUAD) were TYMS, CDKN1, TOP2A, AURKA, and TK-1. For the alcoholic fraction were CDK1, AURKA, AURKB, CCNA2, TYMS, and TK-1. Meanwhile, for the non-polar fraction, we obtained TOP2, PLK1, TYMS, AURKA, CDK1, and NEK2. As we can see in Figure 3, several proteins were shared between the extracts, and because of that the hub proteins were enriched in ShinyGO 0.82 to obtain the pathways altered by these extracts on the evaluated cancers selecting the pathways with an FDR cutoff of 0.05. After that, we compared the overlapping pathways with a Venn diagram tool and found that the three extracts shared five pathways: pyrimidine metabolism, antifolate resistance, progesterone-mediated oocyte maturation, oocyte meiosis, and nucleotide metabolism. The polar aqueous and polar alcoholic fractions shared only the drug metabolism–other enzymes pathway. In contrast, the polar alcoholic and non-polar fractions shared pathways related to cell cycle, viral carcinogenesis, and cellular senescence. Notably, the polar aqueous fraction did not share any pathways with the non-polar fraction (Figure 4).

On the other hand, the hub proteins identified by evaluating the underexpressed genes of BRCA, COAD, and LUAD against the target genes of the extracts were as follows: for the polar aqueous fraction—IL6, PPARG, EGFR, ADCY5, PDE2A, PDE5A, SELP, and CXCR1; for the polar alcoholic fraction—IL6, EGFR, PPARG, FGF2, PDGFRA, CACNA1C, C5AR1, and CXCR1; and for the non-polar fraction—PPARG, IL6, FABP4, ADCY5, PDE2A, PDE5A, C5AR1, and CXCR1. We performed an enriched in ShinyGO 0.82 as mentioned before (Supplementary Materials) and we found five common pathways among the three extracts: pathways in cancer, phospholipase D signaling pathway, coronavirus disease (COVID-19), viral protein interaction with cytokine and cytokine receptor, and human cytomegalovirus infection. For the polar aqueous and non-polar fraction, we found six shared pathways such as purine metabolism, morphine addiction, aldosterone synthesis and secretion, longevity regulating pathway, cGMP-PKG signaling pathway, and lipid and atherosclerosis. Three overlapping pathways were observed for both polar fractions: EGFR tyrosine kinase inhibitor resistance, epithelial cell signaling in *Helicobacter pylori* infection, and Gap junction. However, we did not find any overlapping pathways between the polar alcoholic and non-polar pathways. 

To reinforce the reliability of our network pharmacology predictions, we performed an exploratory validation using public gene expression data (GSE81728) from TK-10 human renal carcinoma cells treated with a *Cordyceps* extract reported by Hwang et al. (2017) [24]. While this cell line is not among the cancer types primarily analyzed in our study, it was selected due to the current lack of available transcriptomic datasets in GEO for *Cordyceps*-treated breast, lung, or colon cancer models. Nevertheless, TK-10 cells exhibited modulation of several pathways consistent with our computational predictions, suggesting that the observed mechanisms may be conserved across multiple cancer types.

The transcriptomic analysis revealed upregulation of pathways related to cell cycle, neuroactive ligand-receptor interaction, and cell adhesion molecules, aligning with our in silico predictions. Notably, downregulated genes were enriched in key cancer-related and inflammatory pathways, including cytokine–cytokine receptor interaction, Wnt, TNF, and MAPK signaling, as well as gastric, colorectal, endometrial, and breast cancer pathways (see Supplementary Materials for data not shown).

Despite the limited availability of suitable datasets and proteomic data, this preliminary validation may provide meaningful evidence consistent with our computational predictions and sets the foundation for future experimental studies. However, it is important to acknowledge that the dataset used includes only a single replicate and lacks biological triplicates, which limits the statistical robustness of the analysis. Moreover, no additional transcriptomic datasets from similar experimental conditions are currently available, further constraining comprehensive validation.

### 2.4. Assessment of Affinity and Binding Modes Between the Hub Proteins and Cordyceps Metabolites by Molecular Docking Simulations

Once the hub proteins in the PPI networks were identified, the binding modes and affinities of metabolites from the different *Cordyceps* extracts were tested against these targets in molecular docking simulations as a strategy of screening to identify the best hit candidates. To begin the simulations, six hub proteins from the overexpressed targets that were common to lung, breast, and colorectal cancers were selected, including AURKA, AURKB, NEK2, TK-1, CDK1, and TYMS. As mentioned in the Methods Section, these proteins were retrieved from PDB and processed using UCSF ChimeraX and AutoDock Tools prior to the simulations. The ligands were constructed, and their energy was minimized using Avogadro. Then, the simulations were run using AutoDock Vina and the corresponding config files generated during the validation of each target. These config files contain the 3D coordinates and sizes of the grid box used during the simulations. It is noteworthy that during the simulations of each hub protein, at least one positive control was included to compare the affinity of a known inhibitor against the metabolites from *Cordyceps* extracts.

Table 2 shows that the binding energy values of some metabolites from *Cordyceps* were comparable to those observed for positive controls. For instance, in NEK2, the binding energies for jiangxienone, deacetylcytochalasin C, and 5α,6α-epoxy-5α-ergosta-7,22-dien-3β-ol were −9.5 kcal/mol, −9.2 kcal/mol, and −9.1 kcal/mol, respectively, while the positive control MBM-5 showed a binding energy of −9.2 kcal/mol. Another example was TYMS, where cryptosporioptide A, cordycepsidone A, and ergosterol peroxide showed binding energies of −8.9 kcal/mol, −8.7 kcal/mol, and −8.2 kcal/mol, respectively. In comparison, the positive control FdUMP (the active form of 5-fluorouracil) showed a binding energy of −8.6 kcal/mol. As can be observed, the difference between the binding energy values tended to be less than 1 kcal/mol. Remarkably, in some targets, the difference between the binding energy values of *Cordyceps* metabolites and the positive control was higher than 1 kcal/mol or even almost 2 kcal/mol. For example, in CDK1, the positive control dinaciclib showed a binding energy of −7.5 kcal/mol, but jiangxienone, cordycepsidone B, and cordycepsidone A showed values of −9.4 kcal/mol, −9.1 kcal/mol, and −9.0 kcal/mol, respectively. In addition, it is important to mention that, although these results were not shown, for some metabolites, positive binding energies were obtained because their size exceeded the binding site of the assayed target.

The results of molecular docking simulations for the rest of the metabolites from *Cordyceps* in every hub protein can be found in Supplementary Materials.

**Figure 5:**
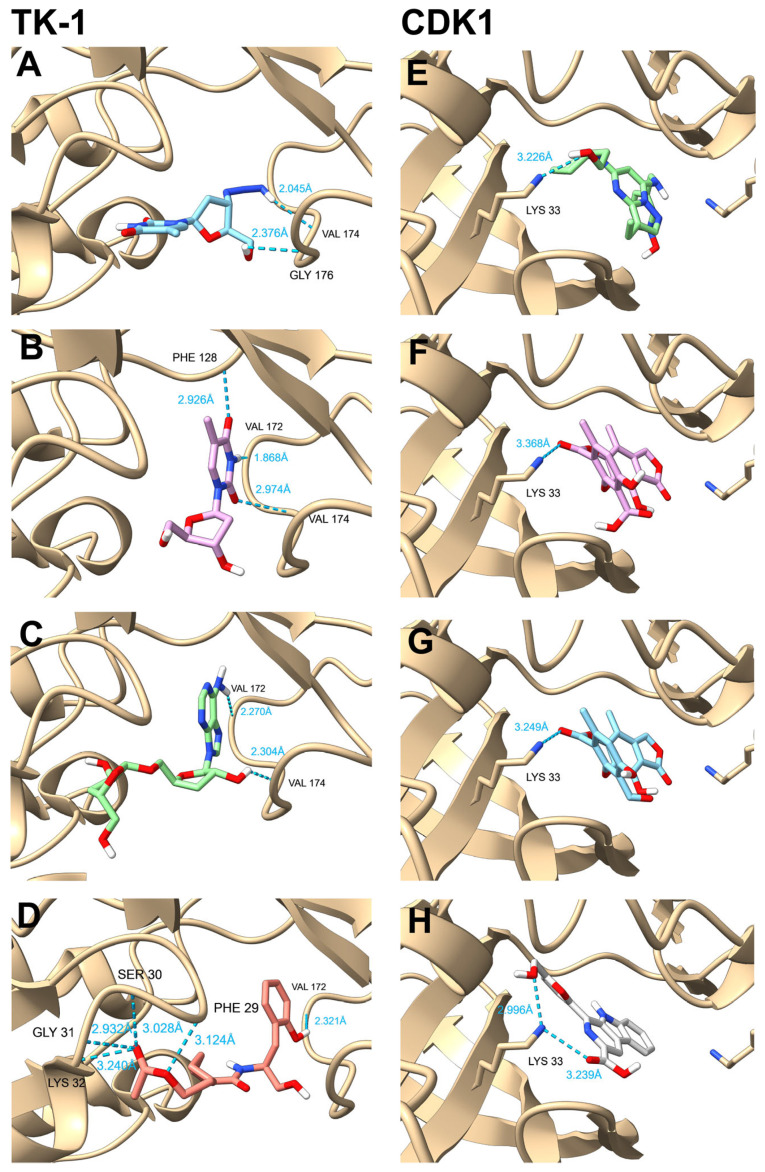
Binding modes of positive controls and selected metabolites from *Cordyceps* against TK-1 and CDK1. (**A**) Interaction between zidovudine (positive control) and TK-1, highlighting hydrogen bonds formed with residues Val174 and Gly176. (**B**) Interaction between the endogenous substrate thymidine to TK-1, showing hydrogen bonds with residues Phe128, Val172, and Val174. (**C**) Interaction between 5′-(3″-Deoxy-β-D-ribofuranosyl)-3′-deoxyadenosine and TK-1, emphasizing hydrogen bonds with residues Val174 and Val176. (**D**) Binding of cordycepiamide C to TK-1, showing hydrogen bonds with key residues (Phe29, Ser30, Gly31, Lys32, Val172). (**E**) Comparative binding of the positive control dinaciclib to CDK1, illustrating hydrogen bond interactions with Lys33. (**F**) Interaction of cordycepsidone B with CDK1, highlighting hydrogen bonds with Lys33, a key residue in the ATP-binding pocket. (**G**) Binding mode of cordycepsidone A to CDK1, emphasizing hydrogen bond interactions with Lys33. (**H**) Interaction between flazin and CDK1, illustrating hydrogen bond interactions involving Lys33. This figure was generated using individual images obtained from ChimeraX.

In addition to the comparison of binding energies, the binding modes of metabolites from *Cordyceps* were also compared in the hub proteins to sustain these observations at a structural level. As can be observed in Figure 5, thymidine established three hydrogen bonds with Phe128, Val172, and Val174 in TK-1. Indeed, according to UniProt, these interactions agreed well with the amino acid residues involved in the binding of thymidine, the substrate of TK-1. In our simulations, the positive control zidovudine established two hydrogen bonds with Val174 and Gly176, while 5′-(3″-Deoxy-β-D-ribofuranosyl)-3′-deoxyadenosine established two hydrogen bonds with Val174 and Val176; and cordycepiamide C established five hydrogen bonds with Phe29, Ser30, Gly31, Lys32, and Val172. It is noteworthy that the interacting amino acid residues observed in the simulations of zidovudine and 5′-(3″-Deoxy-β-D-ribofuranosyl)-3′-deoxyadenosine were reported to be involved in the binding of thymidine inside TK-1, but those interacting residues observed in the simulation of cordycepiamide C were reported to be involved in the binding of ATP, the cosubstrate of TK-1.

Other interesting results were the simulations of CDK1 because the binding of the positive control dinaciclib, cordycepsidone A, cordycepsidone B, and flazin involved Lys33, a key amino acid residue relevant in the binding of ATP. Despite this similar binding mode, it must be highlighted that the affinities of metabolites from *Cordyceps* were higher than dinaciclib.

To strengthen the reliability of our findings, we performed a detailed pharmacophoric analysis using LigandScout 4.5, aiming to identify and compare the key interaction features between the tested *Cordyceps*-derived metabolites and their molecular targets, TK-1, CDK1, and TYMS, relative to the known positive controls. As expected, some metabolites exhibited a conserved pharmacophoric alignment, consistently sharing at least three critical features, such as hydrogen bond donors/acceptors or aromatic moieties, with the reference inhibitors. Notably, the spatial orientation and nature of the pharmacophoric elements, including hydrogen bonding patterns and π-π interactions, were consistent between some of the active metabolites and their respective controls. These observations suggest that metabolites exhibiting closer pharmacophoric resemblance to validated inhibitors are more likely to achieve favorable binding orientations and possibly high docking scores. Interestingly, some of the tested metabolites exhibited moderate binding scores despite lacking significant pharmacophoric similarity to the reference compounds, highlighting a discrepancy between their docking affinities and their pharmacophoric profiles. This inconsistency highlights the limitations of purely in silico predictions and underscores the importance of subsequent experimental validation to confirm biological relevance and binding efficacy. The results of the pharmacophoric analysis are not shown in this manuscript but can be found in Supplementary Materials.

This finding implies that some metabolites from *Cordyceps* may possess even higher affinity to the hub targets involved in pathophysiology of cancer and can be potential hit molecules that can be improved when used as chemical scaffolds for derivatization.

### 2.5. Identification of the Best Potential Candidate Metabolites as Anticancer Drugs Based on Their Pharmacokinetic Properties

After molecular docking simulations, the pharmacokinetic properties of metabolites were predicted using SwissADME to integrate these data with the affinity results of the previous step to identify the best druggable hit molecules from *Cordyceps*. Once the results were generated in SwissADME, the molecular weight, hydrogen bond donors and acceptors, topological surface area, molar refractivity, gastrointestinal absorption, blood–brain barrier permeability, metabolism by cytochromes, and other parameters were collected. Table 3 shows the pharmacokinetic properties predicted for the five best metabolites with the highest affinities in the different hub proteins.

As can be observed in Table 3, the best candidate metabolites to be hit compounds are cordycepsidone A, deoxyerythrostominone, flazin, cordycepiamide C, cordycepiamide D, and cordyceamide A considering that they showed 0 violations in Lipinski rules and high gastrointestinal absorption. These metabolites were exclusively from the polar alcoholic and the non-polar fractions. The predicted pharmacokinetic properties of all metabolites from *Cordyceps* can be found in the Supplementary Materials.

Despite the presence of violations in Lipinski rules, the metabolites may still be promising candidates to be hit molecules, but chemical modifications need to be applied to increase their druglikeness. As shown in Table 3, ergosterol and its derivatives and jianxienone showed a violation in Lipinski rules because their log *p* values were higher than 5. This violation may be fixed by introducing polar groups by derivatization of these metabolites. Cryptosporioptide A, deacetylcytochalasin C, and 5′-(3″-Deoxy-β-D-ribofuranosyl)-3′-deoxyadenosine showed 1 violation to Lipinski rules because they have high numbers of hydrogen bond donors and acceptors (NorO > 10), and this negatively impacts their log *p* values. For instance, cryptosporioptide A, deacetylcytochalasin C, and 5′-(3″-Deoxy-β-D-ribofuranosyl)-3′-deoxyadenosine had consensus log *p* values of −0.66, 1.17, and −0.99, leading to a lower lipophilicity and, consequently, a lower capacity to pass through lipid membranes. However, this violation may be corrected through derivatization of these metabolites by esterification or etherification, and the replacement of groups with a high hydrogen bonding capacity by isosters with a lower hydrogen bonding capacity, among other strategies. Despite demonstrating a significant binding energy in NEK2, 11,11′-dideoxyverticillin A also showed a violation in Lipinski rules because its molecular weight exceeded 500 Da, although this may be improved by the simplification of its chemical structure via pharmacophoric analysis to identify those structural and physicochemical characteristics responsible for its binding to pharmacological targets.

## 3. Discussion

Despite significant advances in cancer biology and treatment, the growing burden of cancer incidence and mortality continues to demand novel therapeutic strategies that are both effective and safe. In this context, the application of network pharmacology and in silico drug discovery tools has emerged as a promising route to identify multitarget agents derived from natural sources with the potential to modulate complex biological systems such as tumors and their microenvironments. Our study integrates these approaches to explore the pharmacological landscape of *Cordyceps* metabolites, with a focus on their predicted interactions with cancer-associated pathways and molecular targets.

The chemical classification of metabolites across the polar aqueous, polar alcoholic, and non-polar fractions of *Cordyceps* extracts revealed a structurally diverse repertoire, including purines, nucleosides, fatty acids, annullatins, and sterols. The clustering patterns observed with ChemMine tools validated the physicochemical rationale behind solvent-based extraction and provided a molecular-level basis for subsequent bioinformatic analyses. The rich presence of nucleosides and ergothioneine in the aqueous phase and bioactive lactones and sterols in lipophilic fractions is consistent with previous phytochemical studies, yet the integration of these data into network pharmacology frameworks remains underexplored [25,26].

Our structure-based target prediction yielded several potential interactions, many of which overlapped with known cancer drivers. Notably, 30.1% of targets were shared across all extracts, and a higher proportion was shared between polar alcoholic and non-polar extracts (59.5%), suggesting that hydrophobic metabolites may possess a broader pharmacological reach as indicated by previous experimental studies [27]. Pathway enrichment analysis via ShinyGO 0.82 confirmed that these targets were involved in fundamental oncogenic mechanisms such as PI3K-Akt signaling, cell cycle regulation, and apoptosis—pathways directly linked to the hallmarks of cancer, including sustained proliferative signaling, evasion of cell death, and replicative immortality [28,29,30].

Further intersecting these predicted targets with transcriptomic datasets from breast, colorectal, and lung cancers highlighted hub proteins such as TYMS, AURKA, CDK1, and TK-1, which are critical regulators of DNA synthesis, mitotic progression, and cell cycle checkpoints. These proteins represent key nodes in the control of tumor proliferation and have been associated with resistance to traditional chemotherapeutics. The overlap of *Cordyceps* metabolite targets with these hub proteins reinforces their potential to modulate central oncogenic axes.

Additionally, the exploratory transcriptomic validation performed in this study may provide concise but meaningful support for the biological relevance of our network pharmacology predictions. Despite TK-10 cells do not represent any of the cancer types primarily analyzed, the response to the *Cordyceps* extract showed convergence with predicted pathways—highlighting upregulation of cell cycle and neuroactive interactions, and downregulation of pro-oncogenic signaling such as MAPK, Wnt, and TNF pathways. These results suggested that *Cordyceps*-derived metabolites may simultaneously activate antitumor mechanisms and suppress tumor-promoting processes across various cancer contexts.

Despite how paradoxical it may seem, an increase in cell cycle–related transcripts can occur under certain inhibitory conditions. A particularly relevant aspect is the possibility of transcriptional compensation, whereby some transcripts are upregulated in response to diminished protein function [31]. This phenomenon, observed in other stress and drug-response models, implies that mRNA levels may not always correlate with protein activity [32]. Thus, *Cordyceps* metabolites could potentially inhibit protein function despite elevated transcription levels, especially if they exert effects at the post-transcriptional or post-translational level. This opens new avenues for investigating non-canonical modes of inhibition by natural compounds.

Molecular docking simulations revealed that multiple metabolites, including cordycepsidones, cordycepiamides, cordyceamides, flazin, deoxyerythrostominone, jiangxienone, and ergosterol derivatives, demonstrated comparable or superior binding affinities to used inhibitors. Importantly, the binding modes of these compounds often involved amino acid residues essential for ATP binding or enzymatic function, suggesting a high degree of structural complementarity. For example, the engagement of TK-1 by cordycepiamide C involved residues associated with ATP binding, not only confirming strong affinity but also indicating potential for regulatory interference beyond competitive inhibition. In addition, the molecular docking findings were further supported by a detailed pharmacophore analysis. Several *Cordyceps*-derived metabolites exhibited pharmacophoric profiles that partially resemble those of the reference inhibitors for TK-1, CDK1, and TYMS. Specifically, these metabolites consistently shared at least three critical pharmacophoric features, such as hydrogen bond donors, hydrogen bond acceptors, and aromatic moieties, which are essential for effective target engagement. This threshold, requiring a minimum of three overlapping pharmacophoric elements, aligns with some accepted criteria in pharmacophore-based methods for drug discovery, and it reflects a meaningful structural congruence with known bioactive compounds [33]. Moreover, the nature and spatial distribution of the pharmacophoric features in the metabolites were consistent with the key interactions required for ATP binding and enzymatic modulation, further reinforcing their potential as functional modulators rather than merely high-affinity binders.

Unfortunately, these *Cordyceps* metabolites have not been studied as extensively as cordycepin, although some reports can be found. For instance, flazin has shown favorable binding energy values of −9.08 kcal/mol and −8.886 kcal/mol in MAPK1 and MAPK8, respectively, in molecular docking simulations [34]. These two targets are serine/threonine protein kinases involved in the MAPK pathway that respond to different stimuli, such as mitogens, damage, or inflammation. MAPK1 (ERK-2), is usually involved in proliferative signaling while MAPK8 (JNK1) is associated with apoptosis or inflammation [35]. In our study, flazin was evaluated against the binding site of ATP in CDK1 yielding a favorable binding energy of −9 kcal/mol, but it is possible that if there was a structural or physicochemical resemblance with ATP, it may bind to the ATP binding site of several kinases. This hypothesis may explain the reported results and suggest that flazin may be a multitarget inhibitor of kinases, but this needs experimental validation because hydrophobic pockets present in kinases may influence selectivity of ligands by affecting their binding efficacy [36]. Additionally, flazin may be a pleiotropic substance because it demonstrated promoting lipolysis while suppressing lipogenesis in human hepatocytes and an increased protection against oxidation through activation of Nrf2 pathway [37,38].

In the case of cordycepiamide D, it has demonstrated the inhibition of nitric oxide production at a concentration of 10 µM in LPS-stimulated RAWR264.7 cells. Other molecules of this family, such as cordycepiamide A and B, also showed favorable inhibitory effects on the NO production, but unfortunately, cordycepiamide C was not evaluated [39].

Deoxyerythrostominone has been evaluated in vitro in cancer cell lines from breast and epidermoid carcinoma of the mouth, and it yielded EC50 of 9.7 µg/mL (29.2 µM) and 23 µg/mL (69.2 µM), respectively [40]. This activity was modest compared with the obtained for ellipticine with 1.22 µM in both cell lines. In the context of our network pharmacology analysis, deoxyerythrostominone was predicted to target AURKA and AURKB, key mitotic regulators frequently associated with oncogenic progression. Therefore, the observed moderate cytotoxicity may not reflect insufficient intrinsic activity, but rather limitations related to target expression in the evaluated cell lines or to compound bioavailability.

As we mentioned, this may be due to a lower penetration of deoxyerythrostominone into the cells, but our pharmacokinetic prediction suggested that its absorption should be high. Another explanation may be that the cell lines used in the assay did not overexpress AURKA or AURKB, interfering with its mechanism of action. This scenario could be sustained by the fact that deoxyerythrostominone exerted a higher cytotoxic effect in breast cancer cells than epidermoid carcinoma cells. This is consistent with our network pharmacology model where one of the overexpressed hub targets identified from the transcriptomic analysis was AURKA.

As part of the ongoing characterization of bioactive metabolites derived from *Cordyceps*, previous studies have identified cordyceamide A as a naturally occurring amide with prominent cytotoxic potential. This compound was functionally assessed in three representative in vitro models: murine fibroblasts (L929), human melanoma cells (A375), and HeLa cells originating from cervical carcinoma [41]. The results revealed a marked reduction in cell viability across all tested lines, with particularly enhanced cytotoxic effects observed in L929 and A375 cells. It is worth noting that, to date, no specific molecular mechanisms have been reported for Cordyceamide A or the other previously discussed compounds. Their modes of action remain unclear, highlighting the need for further in-depth investigation.

The pharmacokinetic evaluation via SwissADME further refined the list of candidate compounds, identifying several metabolites with favorable absorption and druglikeness properties and no Lipinski rule violations. Even metabolites with minor violations—such as excessive lipophilicity or high molecular weight—remain promising as hit compounds, amenable to derivatization strategies such as esterification or fragment-based simplification. This underscores the potential of *Cordyceps*-derived compounds as chemical scaffolds in rational drug design.

Our findings underscore that the physicochemical and pharmacokinetic profiles of *Cordyceps* metabolites vary notably depending on their extraction fraction, influencing their potential applicability in preclinical and clinical settings.

Metabolites in the aqueous fraction, such as thymidine, uridine, and 2′-deoxyuridine, display high aqueous solubility and adequate gastrointestinal absorption; however, their low lipophilicity limits passive cellular membrane permeability. Enhancing their intratumoral delivery could require formulation into aqueous-based nanoemulsions or hydrophilic nanoparticles, particularly beneficial for tumors with permeable vascular structures.

Conversely, the alcoholic fraction, including metabolites like cordycepsidone A and B, flazin, and deoxyerythrostominone, exhibits highly favorable drug-like characteristics—optimal gastrointestinal uptake, compliance with Lipinski’s rule of five, and moderate CYP enzyme interactions—making these compounds prime candidates for preclinical evaluations. Their balanced solubility profile permits versatile formulation strategies, facilitating testing in animal models of breast, colon, and lung cancers.

Compounds in the non-polar fraction, notably ergosterol derivatives and fatty acids, are characterized by high lipophilicity, enhancing membrane permeability yet hindering aqueous solubility. Their effective translation to in vivo studies would thus necessitate specialized lipid-based carriers, such as liposomes or emulsions, to ensure adequate systemic distribution and intratumoral bioavailability.

Importantly, none of the evaluated metabolites showed predicted permeability across the blood–brain barrier (as determined by computational predictions using SwissADME), highlighting their preferential applicability for peripheral tumors. Nevertheless, given the inherent limitations of computational models, experimental validation may reveal differences in actual permeability, underscoring the potential of specialized delivery systems to facilitate central nervous system targeting.

These findings gain further significance considering the current understanding of cancer as a dynamic and heterogeneous disease. Several of the predicted targets and enriched pathways, including cellular senescence, immune signaling, and metabolic reprogramming, align with emerging hallmarks such as cellular plasticity and tumor-promoting inflammation. Moreover, the involvement of metabolites in modulating components of the tumor microenvironment—such as cytokine interactions and viral oncogenesis—suggests their potential to overcome therapeutic resistance through multitarget action.

Taken together, our data supports the utility of *Cordyceps* metabolites as a rich source of hit compounds capable of engaging diverse molecular targets implicated in cancer progression. By leveraging a network pharmacology approach, we provide mechanistic insights into how natural products may function beyond single-target inhibition, offering a framework for the development of synergistic, low-toxicity, multitarget anticancer therapies. These results not only validate traditional medicinal claims associated with *Cordyceps* but also pave the way for experimental validation and structure-activity optimization of its most promising metabolites.

This study is based on computational approaches that, while powerful for hypothesis generation, require experimental validation to confirm biological activity and therapeutic efficacy. Similar studies on natural product-derived compounds have reinforced the translational relevance of in silico predictions through complementary in vitro assays [42]. The predicted protein–metabolite interactions, binding affinities, and pathway modulations were obtained through in silico simulations and databases, which may not fully capture the complexity of cellular environments or off-target effects. Additionally, metabolite concentrations used in molecular docking do not reflect physiological bioavailability, and pharmacokinetic predictions, although useful, are estimative and context dependent. Moreover, the extraction process and metabolite annotation were literature-based and may omit minor constituents or unreported compounds with relevant activity.

Future research should include experimental validation of the top candidate metabolites using in vitro and in vivo cancer models to confirm their cytotoxicity, selectivity, and mechanism of action. High-throughput screening, gene expression analysis, and pathway inhibition assays could provide further evidence of their multitarget effects. Additionally, structural optimization via medicinal chemistry approaches may enhance the pharmacological properties of the most promising compounds, such as improving lipophilicity, reducing molecular weight, or enhancing metabolic stability. Finally, the development of formulations or delivery systems, especially for poorly soluble metabolites, may facilitate their transition into preclinical development and potential clinical evaluation.

## 4. Materials and Methods

To elucidate the possible mechanisms of action of the different *Cordyceps* extracts responsible for reported anticancer activity, the following general methodology was followed: (1) construction of a database containing the reported *Cordyceps* metabolites in the different extracts; (2) structure-based target prediction of metabolites; (3) comparison of the predicted targets against key proteins involved in the pathophysiology of breast cancer, lung cancer, and colorectal cancer to identify the potential targets affected by the *Cordyceps* metabolites in each tumor; (4) analysis of protein–protein interaction networks of the affected targets to identify hub proteins and potential pathways regulated by *Cordyceps* metabolites; (5) assessment of affinity and binding modes between the hub proteins and *Cordyceps* metabolites by molecular docking simulations; and (6) identification of the best potential candidate metabolites as anticancer drugs based on their pharmacodynamic and pharmacokinetic properties. Each step of this general methodology is described separately below.

### 4.1. Compilation of Metabolites from Cordyceps Extracts into a Literature-Based Database

First, an intensive review of literature was conducted to search for reported metabolites present in the different extracts of *Cordyceps*. These metabolites were recorded in a database and classified in three categories: (1) polar aqueous fraction, (2) polar alcoholic fraction, and (3) non-polar fraction. For the polar alcoholic fraction, metabolites identified in extractions with methanol, ethanol, and butanol were included. For the non-polar fraction, the metabolites identified in extractions with hexane, chloroform, and ethyl acetate were included. For each metabolite, the SMILES was collected from PubChem [43].

### 4.2. Chemical Classification of Metabolites from Cordyceps Extracts

Before the elucidation of the biological actions, a chemical classification of the metabolites was performed to identify the most representative families of natural products contained in each extract. These classifications were carried out using ChemMine tools (https://chemminetools.ucr.edu/) accessed on 13 February 2025 [44]. Three lists of SMILES corresponding to the metabolites of each extract were introduced in the option ‘Add compounds’, then, the section ‘Cluster’ was accessed, and multidimensional scaling was selected. The dimensions and the similarity cutoff selected were 2D and 0.4, respectively.

### 4.3. Structure-Based Target Prediction of Metabolites from Cordyceps Extracts

The SMILES of each metabolite was introduced into the Swiss Target Prediction (STP) [45,46] or the Similarity Ensemble Approach (SEA) to perform a structure-based target prediction. This prediction is based on the structural similarity shared between the molecule of interest and reference molecules whose targets are known. Once the predicted targets were obtained, their corresponding common names, Uniprot IDs, ChEMBL IDs, target class, probability of the prediction, and known actives (3D/2D) were collected for each metabolite. After this step, the lists of the predicted targets of all metabolites from each extract were fused into a single list. It is noteworthy that duplicated targets were deleted.

### 4.4. Comparison of the Predicted Metabolite Targets and Tumor-Associated Targets in Lung, Colorectal, and Breast Cancers

To test if the predicted targets of the different extracts from *Cordyceps* may modulate relevant targets involved in the pathophysiology of lung, colorectal and breast tumors, comparisons through Venn diagrams were performed. Before the comparisons, the differentially altered genes of these tumor types were retrieved from GEPIA2 [47]. The search began by accessing ‘Expression Analysis’, then the option ‘Differential Genes’ was selected. Once there, the keys ‘BRCA’, COAD’, and ‘LUAD’ were chosen as dataset options to retrieve the genes from breast cancer, colon adenocarcinoma, and lung adenocarcinoma, respectively. The additional options used to perform the search were a Log_2_FC of 2 and −2, a q-value cutoff of 0.01, and limma as the differential method. With these options, overexpressed and underexpressed genes were retrieved from GEPIA2 for each tumor type. After this step, the comparison of the predicted targets of the different extracts from *Cordyceps* against the overexpressed and underexpressed genes of each tumor type was performed using the Draw Venn Diagram tool from the University of Gent (https://bioinformatics.psb.ugent.be/webtools/Venn/) accessed on 20 February 2025. The common targets from these comparisons were collected for further analysis.

### 4.5. Protein–Protein Interaction Networks to Identify Hub Proteins and Potential Pathways Regulated by the Cordyceps Extracts

The collected common targets identified in the comparisons between the predicted targets for each *Cordyceps* extract and overexpressed and underexpressed genes for each tumor type were introduced into STRING (https://string-db.org/) accessed on 25 Febrary 2025 [48] to construct protein–protein interaction (PPI) networks. These networks were sent to Cytoscape [49] to identify hub proteins. In this step, the scores of nodes were calculated, and the top three targets affected for each extract in the overexpressed and underexpressed cancer genes were ranked using maximal clique centrality (MCC) as the centrality measure using Cytohubba [50]. Additionally, enrichment analyses of the top three targets affected by the extracts were performed in ShinyGO 0.82 [51] to identify the possible signaling pathways, metabolic pathways, or other cellular processes that may be modulated by *Cordyceps* in the different tumor types. Before enrichment, the top three targets for each cancer type were compiled into a single list according to the extract used (polar aqueous, polar alcoholic, and non-polar). The enrichment analyses were conducted using the *Homo sapiens* as the model, an FDR cutoff of 0.05, the removal of redundancy, and a minimum and maximum pathway size of 2 and 5000, respectively. Once the enriched pathways were identified for each extract, a Venn diagram was constructed using the Draw Venn Diagram tool from the University of Gent (https://bioinformatics.psb.ugent.be/webtools/Venn/) accessed on 26 Febrary 2025 to compare the results and highlight the signaling or metabolic pathways shared among the different extracts. These comparisons were performed separately for overexpressed and underexpressed genes to distinguish potential extract-specific effects on upregulated and downregulated targets. Additionally, to further support our predictions, we performed a transcriptomic analysis in R 4.3.1 and RStudio 2024.12.1+563 using a public dataset (GSE81728) corresponding to TK-10 cancer cells treated with a *Cordyceps* extract reported by Hwang et al. (2017) [24]. It is important to note that the dataset included only one replicate for both the control and treated groups, and the analysis was intended solely as preliminary evidence.

### 4.6. Molecular Docking of Hub Proteins with Cordyceps Metabolites

Once the most relevant targets responsible for the anticancer activity of *Cordyceps* extracts in the different tumor types were identified, molecular docking simulations were used as a screening method to determine the best candidates for inhibitor drugs. To perform the docking simulations, the different metabolites were constructed as 3D coordinates files .mol2 by introducing the collected SMILES in Avogadro. Hydrogen atoms were added to the different molecules considering a physiological pH of 7.4. Additionally, the geometry was optimized using the force field MMFF94 and the Steepest Descent algorithm. The protein targets were retrieved from the Protein Data Bank and preprocessed using UCSF ChimeraX [52]. Solvent residues and crystallized ligands were removed. After that, each protein was introduced into AutoDock Tools [53], and the polar hydrogens were added, the non-polar hydrogens were removed, and the Kollman charges were added. Each ligand was also introduced in AutoDock Tools and prepared in a similar manner, but Gasteiger charges were added instead of Kollman charges. The grid boxes were defined considering the relevant amino acid residues involved in binding or catalysis according to information reported in UniProt or literature. The molecular docking simulations were carried out in AutoDock. It is noteworthy that the simulations were validated using the redocking methodology. Specific details about the molecular docking simulations can be found in the Supplementary Materials. Finally, to further support the results obtained in the molecular docking simulations, we conducted a pharmacophoric analysis using LigandScout 4.5. There, we performed the creation and the alignment of pharmacophores using the docked poses obtained for the *Cordyceps* metabolites and the positive controls in TK-1, TYMS, and CDK1. The representations were generated with LigandScout 4.5 (2025), Inte:Ligand GmbH, Vienna, Austria (www.inteligand.com/ligandscout) accessed on 18 April 2025.

### 4.7. Selection of Potential Anticancer Metabolites Based on Pharmacodynamic and Pharmacokinetic Properties

The pharmacokinetic properties of metabolites from *Cordyceps* were predicted by introducing the SMILES of each molecule in the SwissADME tool [54]. Once the results were generated, the molecular weight, hydrogen bond donors and acceptors, topological surface area, molar refractivity, gastrointestinal absorption, blood–brain barrier permeability, metabolism by cytochromes, and other parameters were collected. These data were combined with the affinity values obtained in the previous step, to identify the best druggable candidate hit molecules in *Cordyceps* extracts.

## 5. Conclusions

This study provides a comprehensive in silico characterization of bioactive metabolites from *Cordyceps* extracts, revealing their potential as multitarget anticancer agents. Through the integration of chemical classification, structure-based target prediction, pathway enrichment, and molecular docking simulations, we identified key interactions between *Cordyceps*-derived compounds and proteins critically involved in tumor proliferation, cell cycle regulation, and therapeutic resistance. Several metabolites, notably from the polar alcoholic and non-polar fractions, exhibited strong binding affinities and favorable pharmacokinetic properties, positioning them as promising hit candidates. Our findings support the concept that natural products, when analyzed under the framework of network pharmacology, can serve as powerful tools for discovering multitarget therapeutic agents capable of modulating cancer hallmarks. These results also highlight the relevance of fungi as a source of pharmacologically active compounds and lay the groundwork for further experimental validation and rational drug design based on *Cordyceps* metabolites. Ultimately, this work contributes to the advancement of integrative strategies in anticancer drug discovery, promoting the development of more effective, safer, and personalized therapeutic options.

## Figures and Tables

**Table 1:** Top hub proteins obtained from polar aqueous, polar alcoholic, and non-polar fraction of *Cordyceps* against differential genes over- and underexpressed from BRCA, COAD, and LUAD.

Cancer Type	Classification	Polar Aqueous Fraction	Polar Alcoholic Fraction	Non-Polar Fraction
BRCA	Overexpressed	TYMS	CDK1	TOP2
		CDKN1	AURKA	PLK1
		TOP2A	CCNA2	TYMS
	Underexpressed	IL6	IL6	PPARG
		PPARG	EGFR	IL6
		EGFR	PPARG	FABP4
COAD	Overexpressed	AURKA	AURKA	AURKA
		TYMS	CCNA2	TYMS
		CDKN1	TYMS	CDK1
	Underexpressed	ADCY5	FGF2	ADCY5
		PDE2A	PDGFRA	PDE2A
		PDE5A	CACNA1C	PDE5A
LUAD	Overexpressed	TK-1	TK-1	NEK2
		AURKA	AURKA	TOP2
		CDKN1/TOP2A	AURKB	AURKA
	Underexpressed	IL6	IL6	IL6
		SELP	C5AR1	C5AR1
		CXCR1	CXCR1	CXCR1

**Table 2:** Top five binding energies obtained from molecular docking simulations between selected hub proteins and metabolites from *Cordyceps* extracts.

Target	Ligand	Binding Energy (kcal/mol)
AURKA	Cordycepsidone A	−9.3
	Cordycepsidone B	−9.1
	Ergosterol peroxide	−9.0
	5α,6α-Epoxy-5α-ergosta-7,22-dien-3β-ol	−8.9
	Ergosterol	−8.5
	Danusertib *	−8.3
	Alisertib *	−8.2
AURKB	Ergosta-4,6,8 (14),22-tetraen-3-one	−9.7
	Cordycepsidone A	−9.2
	Cordycepsidone B	−9.0
	Ergosterol	−8.7
	Deoxyerythrostominone	−8.6
	Danusertib *	−8.3
CDK1	Jiangxienone	−9.4
	Cordycepsidone B	−9.1
	Cordycepsidone A	−9.0
	Flazin	−9.0
	Ergosta-4,6,8 (14),22-tetraen-3-one	−8.9
	Dinaciclib *	−7.5
TK-1	Thymidine	−9.1
	Cordycepiamide C	−8.6
	Uridine	−8.6
	5′-(3″-Deoxy-β-D-ribofuranosyl)-3′-deoxyadenosine	−8.5
	Cordycepiamide D	−8.5
	Zidovudine *	−7.6
NEK2	Jiangxienone	−9.5
	Deacetylcytochalasin C	−9.2
	5α,6α-Epoxy-5α-ergosta-7,22-dien-3β-ol	−9.1
	11,11′-Dideoxyverticillin A	−8.9
	Cordyceamide A	−8.9
	MBM-5 *	−9.2
TYMS	Cordycepsidone B	−8.9
	Cryptosporioptide A	−8.9
	Cordycepsidone A	−8.7
	Ergosterol peroxide	−8.2
	5α,6α-Epoxy-5α-ergosta-7,22-dien-3β-ol	−8.2
	FdUMP *	−8.6

* Positive control. AURKA: Aurora kinase A; AURKB: Aurora kinase B; CDK1: Cyclin-dependent kinase 1; TK-1: Thymidine kinase 1; NEK2: NIMA-related kinase 2; TYMS: Thymidylate synthetase; FdUMP: Fluorodeoxyuridylate.

**Table 3:** Pharmacokinetic profile of selected *Cordyceps*-derived metabolites with potential affinity against hub proteins.

Metabolite (Extract)	GI Absorption	BBB Permeability	P-Gp Substrate	CYP Inhibition	Lipinski Violations	Target(s)
Cordycepsidone A (PALC)	High	No	No	CYP3A4 CYP2C9	0	AURKA AURKB CDK1 TYMS
Cordycepsidone B (PALC)	Low	No	Yes	No	0	AURKA AURKB CDK1 TYMS
Ergosterol peroxide (NP)	High	No	No	No	1 *	AURKA TYMS
5α,6α-Epoxy-5α-ergosta-7,22-dien-3β-ol (PALC)	High	No	No	CYP2C9	1 *	AURKA NEK2 TYMS
Ergosterol (NP)	Low	No	No	CYP2C9	1 *	AURKA AURKB
Ergosta-4,6,8 (14),22-tetraen-3-one (NP)	Low	No	No	CYP2C9	1 *	AURKB TYMS CDK1
Deoxyerythrostominone (PALC/NP)	High	No	No	CYP1A2 CYP2C9	0	AURKA AURKB
Jiangxienone (NP)	High	No	Yes	CYP2C9 CYP3A4	1 *	CDK1 NEK2
Flazin (PALC/NP)	High	No	No	CYP1A2 CYP2D6	0	CDK1
Thymidine (PAQ)	High	No	No	No	0	TK-1
Cordycepiamide C (PALC)	High	No	No	No	0	TK-1
Uridine (PAQ)	Low	No	No	No	0	TK-1
5′-(3″-Deoxy-β-D-ribofuranosyl)-3′-deoxyadenosine (PALC)	Low	No	No	No	1 †	TK-1
Cordycepiamide D (PALC)	High	No	Yes	No	0	TK-1
Deacetylcytochalasin C (PALC)	High	No	No	CYP2C9	1 †	NEK2
11,11′-Dideoxyverticillin A (NP)	Low	No	Yes	No	1 ‡	NEK2
Cordyceamide A (PALC)	High	No	Yes	CYP2C19 CYP2C9 CYP2D6 CYP3A4	0	NEK2
Cryptosporioptide A (PALC/NP)	Low	No	No	No	1 †	TYMS

* log *p* > 5; † NorO > 10; ‡ MW > 500 Da. PALC: Polar alcoholic fraction; PAQ: Polar aqueous fraction; NP: Non-polar fraction; GI: Gastrointestinal; P-Gp: P-Glycoprotein; BBB: Blood–brain barrier; AURKA: Aurora kinase A; AURKB: Aurora kinase B; CDK1: Cyclin-dependent kinase 1; TK-1: Thymidine kinase 1; NEK2: NIMA-related kinase 2; TYMS: Thymidylate synthetase.

## Data Availability

All data generated or analyzed during this study are included in this published article and its Supplementary Information files.

## Supplementary Materials

The following supporting information can be downloaded at: https://www.mdpi.com/article/10.3390/ph18050667/s1, SupplementaryMaterials.zip.

## Abbreviations

The following abbreviations are used in this manuscript:

AURKA	aurora kinase A
AURKB	aurora kinase B
BBB	blood–brain barrier
BRCA	breast invasive adenocarcinoma
CDK1	cyclin-dependent kinase 1
COAD	colon adenocarcinoma
DNA	deoxyribonucleic acid
FDR	false discovery rate
IARC	International Agency for Research on Cancer
LUAD	lung adenocarcinoma
MCC	maximal clique centrality
NEK2	NIMA related kinase 2
NCCN	National Comprehensive Cancer Network
P-Gp	P-Glycoprotein
PDB	Protein Data Bank
PPI	protein–protein interaction
ROS	reactive oxygen species
SASP	senescence-associated secretory phenotype
TK-1	thymidine kinase 1
TME	tumor microenvironment
TYMS	thymidylate synthetase
