# Supplementary material for: The Cordyceps Genus as a Potential Source of Bioactive Compounds for Adjuvant Cancer Therapy: A Network Pharmacology Approach
Source: Pharmaceuticals (Basel). 2025 Apr 30;18(5):667. doi: 10.3390/ph18050667 (PMC12115173; doi:10.3390/ph18050667)
Supplement: Supplementary file 1 [file pharmaceuticals-18-00667-s001.zip › pharmaceuticals-3597034-supplementary/Supplementary Materials/Redocking, PPI Networks, and pharmacophoric analysis.pdf]

The Cordyceps Genus as a Potential Source of Bioactive Compounds for Adjuvant Cancer Therapy: A Network Pharmacology Approach

Jose Luis Gonzalez-Llerena[1], Daniela Treviño-Almaguer[1], Jesus Alejandro Leal-Mendez[1], Gael Garcia-Valdez[1], Arely Guadalupe Balderas-Moreno[1], Michel Stéphane Heya[2], Isaias Balderas-Renteria[1], Maria Del Rayo Camacho-Corona[3*] and Bryan Alejandro Espinosa-Rodriguez[4*]

Authors affiliation:

[1] Universidad Autonoma de Nuevo Leon, School of Chemistry, Laboratory of Molecular Pharmacology and Biological Models, Monterrey, Nuevo Leon, 64570, Mexico.

[2] Universidad Autonoma de Nuevo Leon, Faculty of Public Health and Nutrition, Monterrey 64460, Mexico.

[3] Universidad Autonoma de Nuevo Leon, School of Chemistry, Laboratory of Pharmaceutical Chemistry, Monterrey, Nuevo Leon, 64570, Mexico.

[4] Universidad Autonoma de Nuevo Leon, School of Chemistry, Laboratory of Biopharmacy, San Nicolas de los Garza, Nuevo Leon, 66451, Mexico.

**Corresponding authors*:

Maria Del Rayo Camacho-Corona

Universidad Autonoma de Nuevo Leon, School of Chemistry, Laboratory of Pharmaceutical Chemistry, San Nicolas de los Garza, Nuevo Leon, 66451, Mexico.

E-mail: maria.camachocn@uanl.edu.mx

Bryan Alejandro Espinosa-Rodriguez

Universidad Autonoma de Nuevo Leon, School of Chemistry, Laboratory of Biopharmacy, San Nicolas de los Garza, Nuevo Leon, 66451, Mexico.

E-mail: bespinosar@uanl.edu.mx

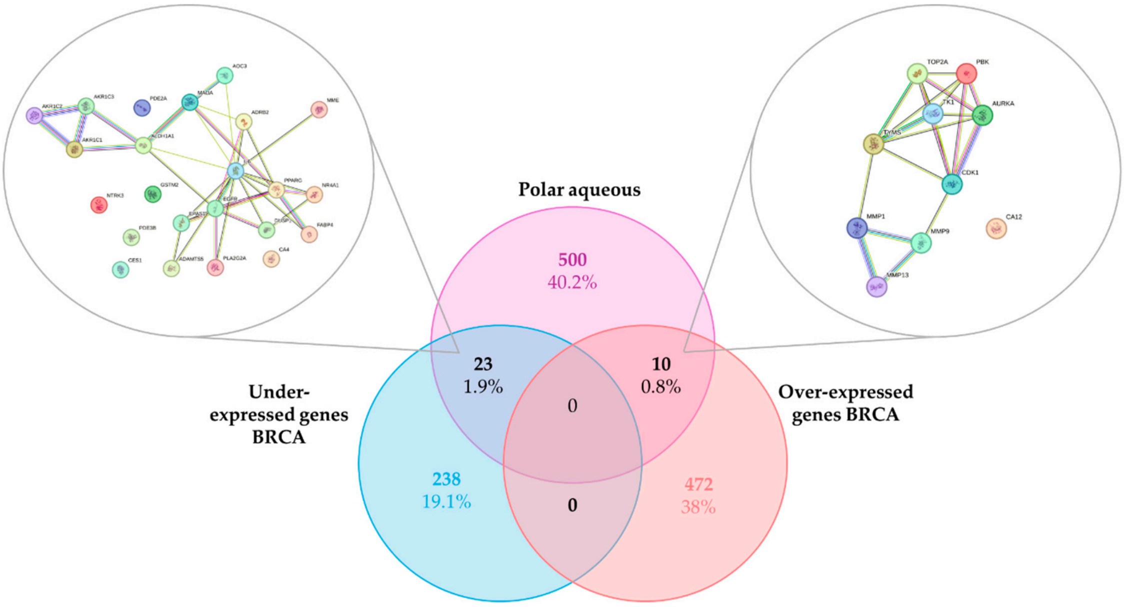

Figure S1. Venn diagram of the overlapping proteins between the differential genes (over and under-expressed) of BRCA and the predicted targets of the polar aqueous extract of *Cordyceps* with the PPI network corresponding to the over and under-expressed genes.

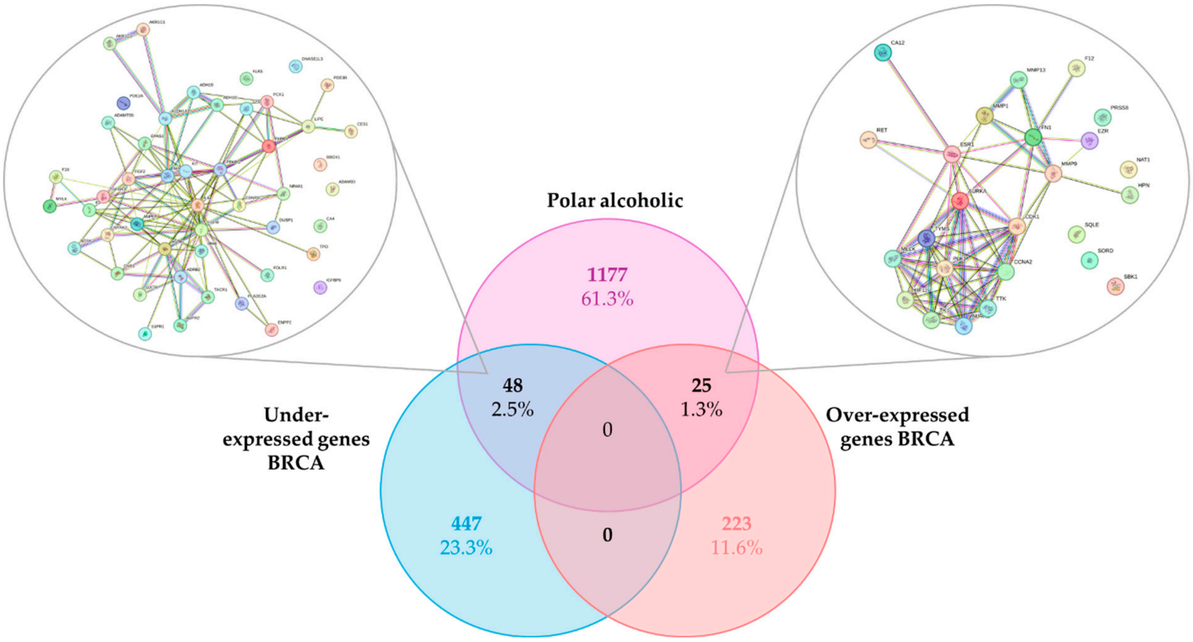

Figure S2. Venn diagram of the overlapping proteins between the differential genes (over and under-expressed) of BRCA and the predicted targets of the polar alcoholic extract of *Cordyceps* with the PPI network corresponding to the over and under-expressed genes.

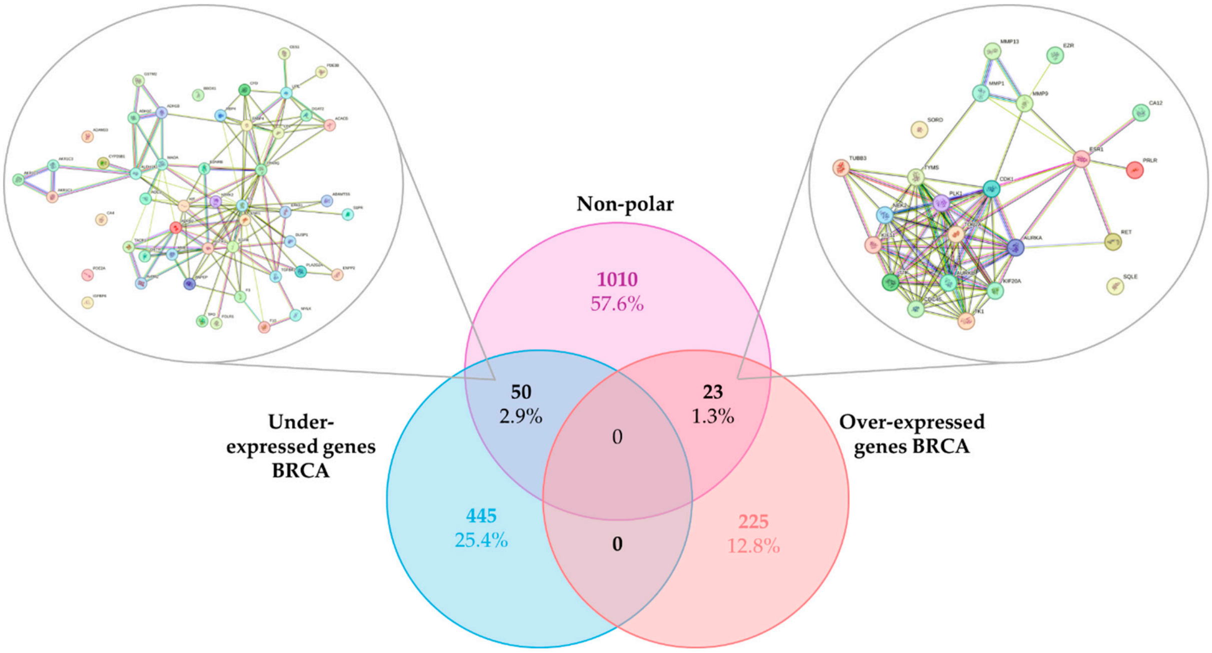

Figure S3. Venn diagram of the overlapping proteins between the differential genes (over and under-expressed) of BRCA and the predicted targets of the non-polar extract of *Cordyceps* with the PPI network corresponding to the over and under-expressed genes.

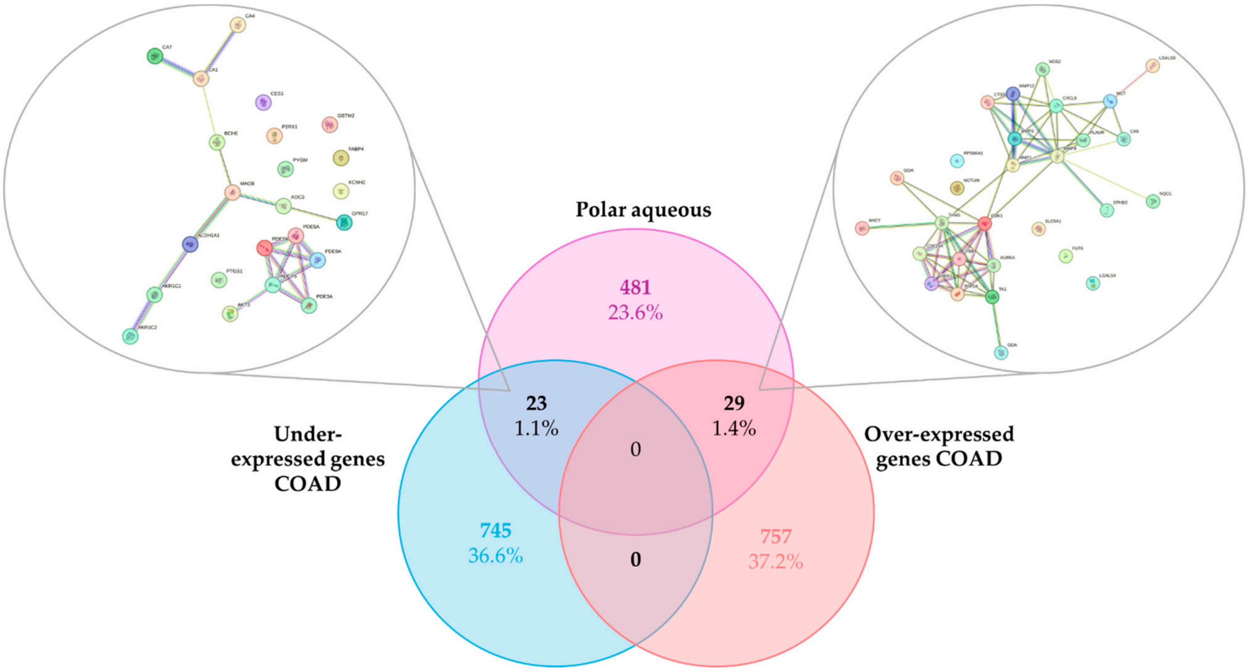

Figure S4. Venn diagram of the overlapping proteins between the differential genes (over and under-expressed) of COAD and the predicted targets of the polar aqueous extract of *Cordyceps* with the PPI network corresponding to the over and under-expressed genes.

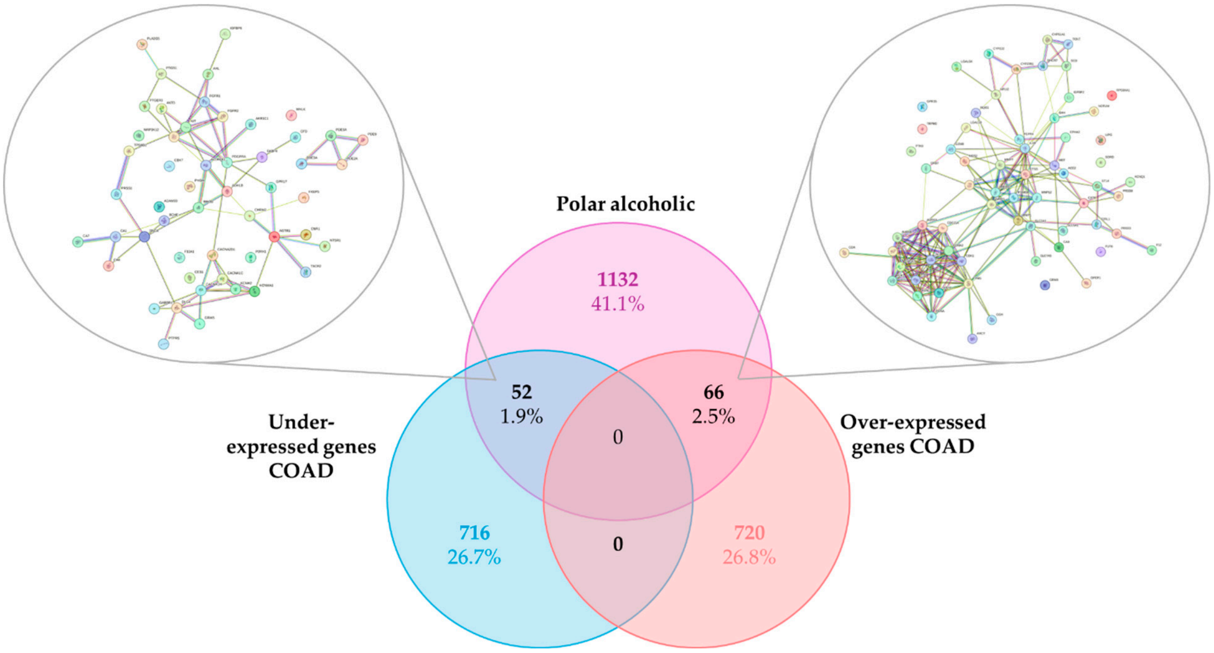

Figure S5. Venn diagram of the overlapping proteins between the differential genes (over and under-expressed) of COAD and the predicted targets of the polar alcoholic extract of *Cordyceps* with the PPI network corresponding to the over and under-expressed genes.

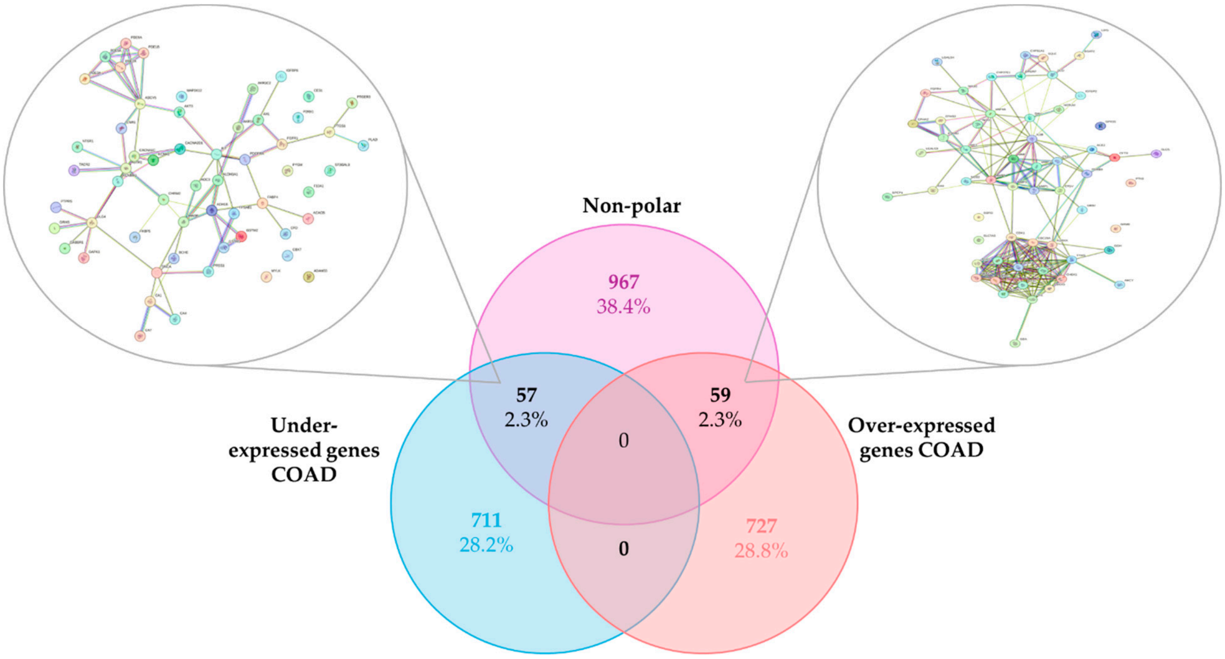

Figure S6. Venn diagram of the overlapping proteins between the differential genes (over and under-expressed) of COAD and the predicted targets of the non-polar extract of *Cordyceps* with the PPI network corresponding to the over and under-expressed genes.

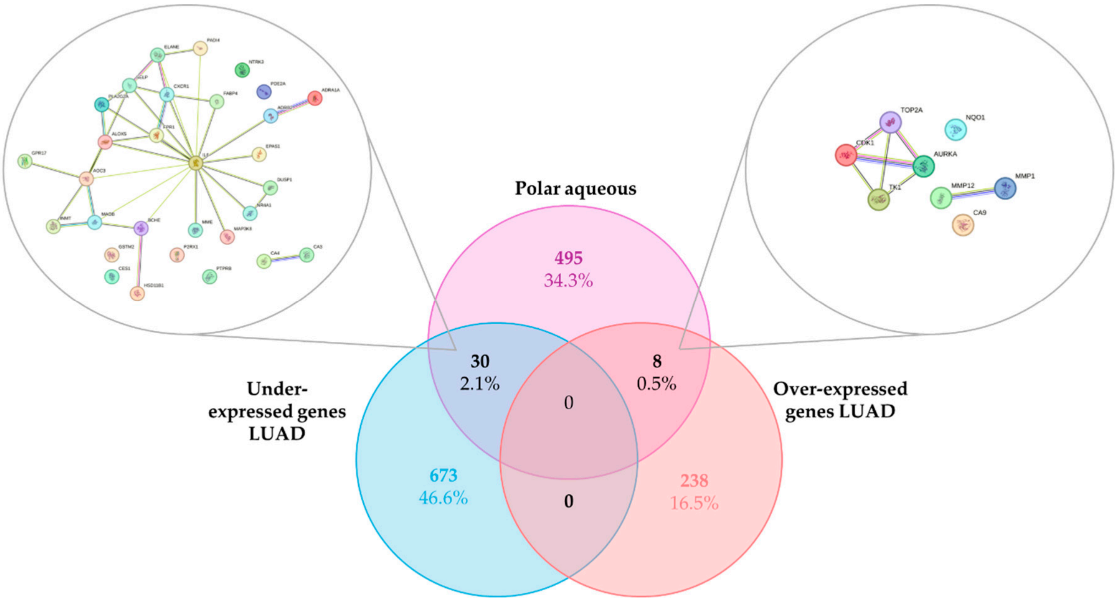

Figure S7. Venn diagram of the overlapping proteins between the differential genes (over and under-expressed) of LUAD and the predicted targets of the polar aqueous extract of *Cordyceps* with the PPI network corresponding to the over and under-expressed genes.

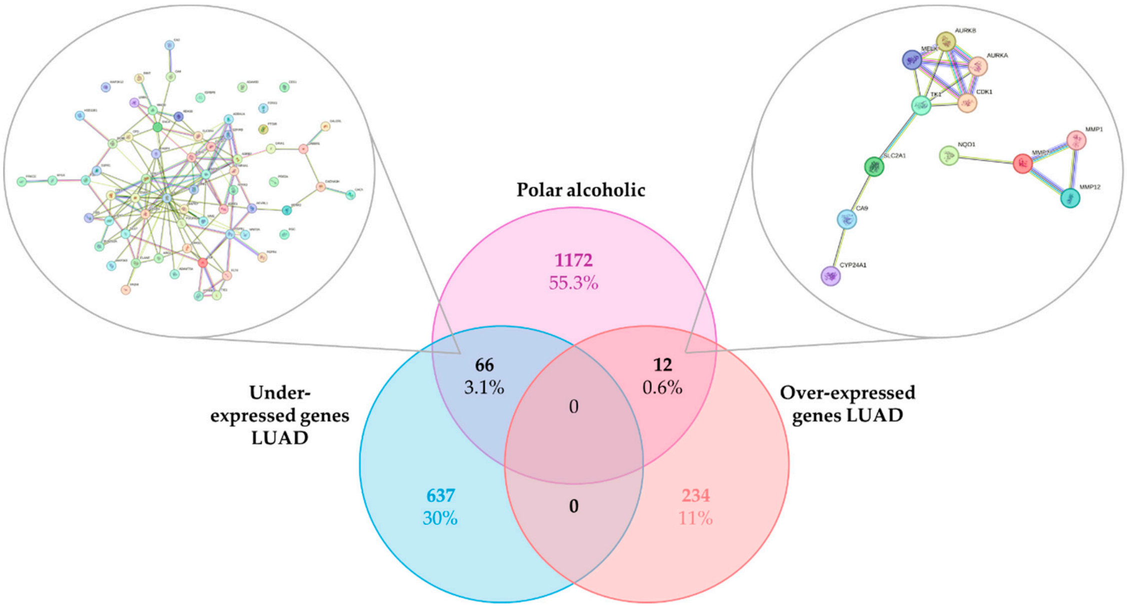

Figure S8. Venn diagram of the overlapping proteins between the differential genes (over and under-expressed) of LUAD and the predicted targets of the polar alcoholic extract of *Cordyceps* with the PPI network corresponding to the over and under-expressed genes.

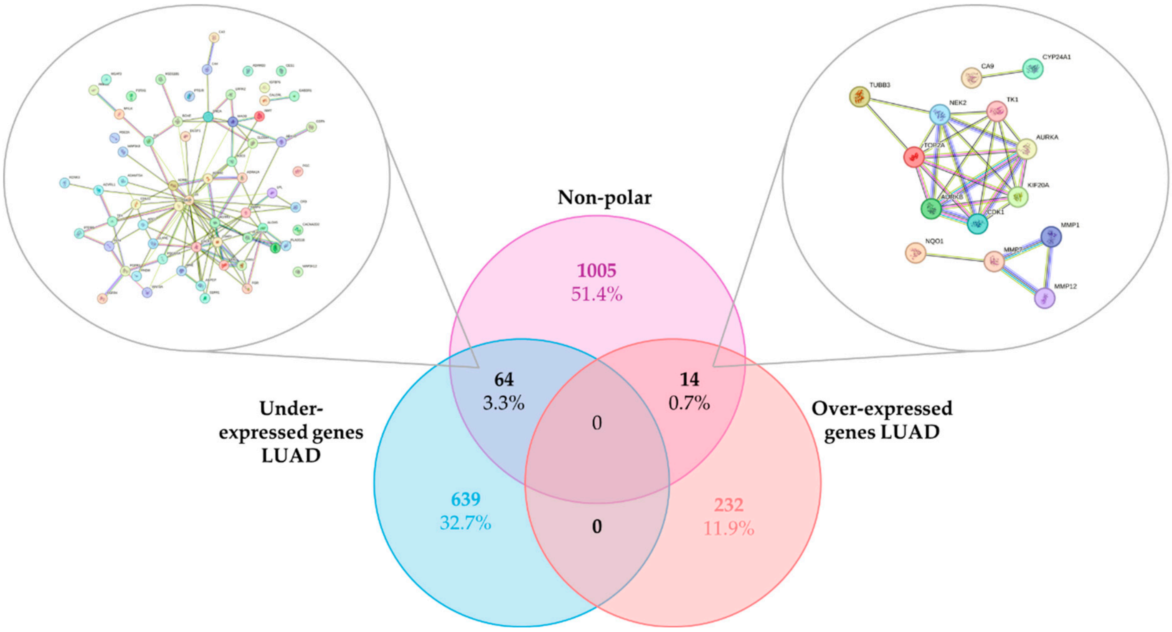

Figure S9. Venn diagram of the overlapping proteins between the differential genes (over and under-expressed) of LUAD and the predicted targets of the non-polar extract of *Cordyceps* with the PPI network corresponding to the over and under-expressed genes.

Table S1. Targets with their PDB code

Target	Code
AURKA	3CHO
AURKB	5K3Y
CDK1	6GU6
NEK2	2W5A
TK1	2B8T
TYMS	5X66

Table S2. Targets with their coordinates

Target	Grid Box Size			Grid Box Coordinates			RMSD
	X	Y	Z	X	Y	Z	
AURKA	13.077	52.937	97.197	15	15	15	4.546
AURKB	10.5397	-1.48045	3.04397	15.3163	15.7313	12.7554	7.457
CDK1	22.9455	21.9241	-1.1965	15	15	15	6.089
NEK2	-26.896	11.233	16.341	15	15	15	3.908
TK1	-18.6276	15.1095	45.3284	9.63916	9.37801	15.7574	2.926
TYMS	143.868	146.949	24.988	15	15	15	3.466

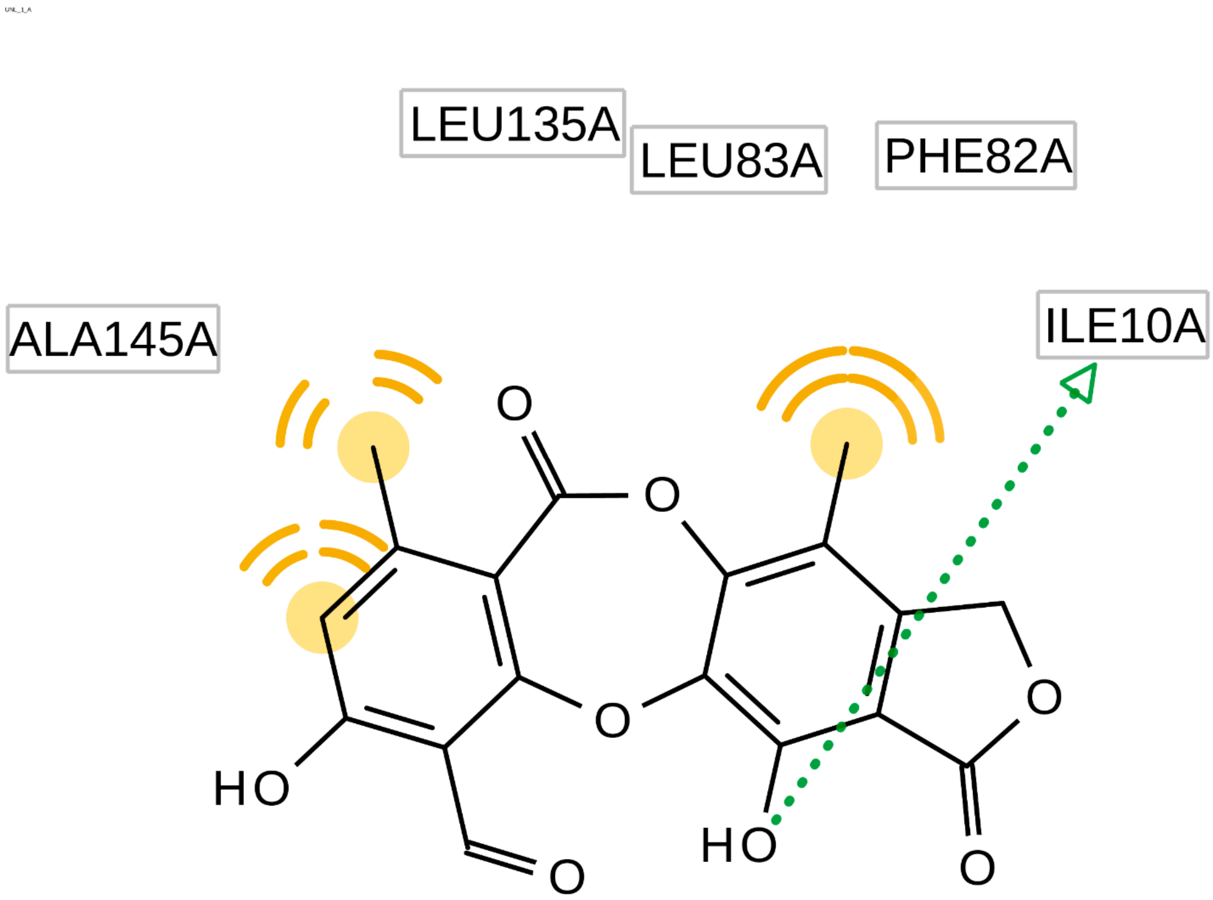

Figure S10. Pharmacophore analysis of the complex CDK1 and Cordycepsidone A in a 2D representation. This representation was generated with LigandScout 4.5 (2025), Inte:Ligand GmbH, Vienna, Austria (www.inteligand.com/ligandscout).

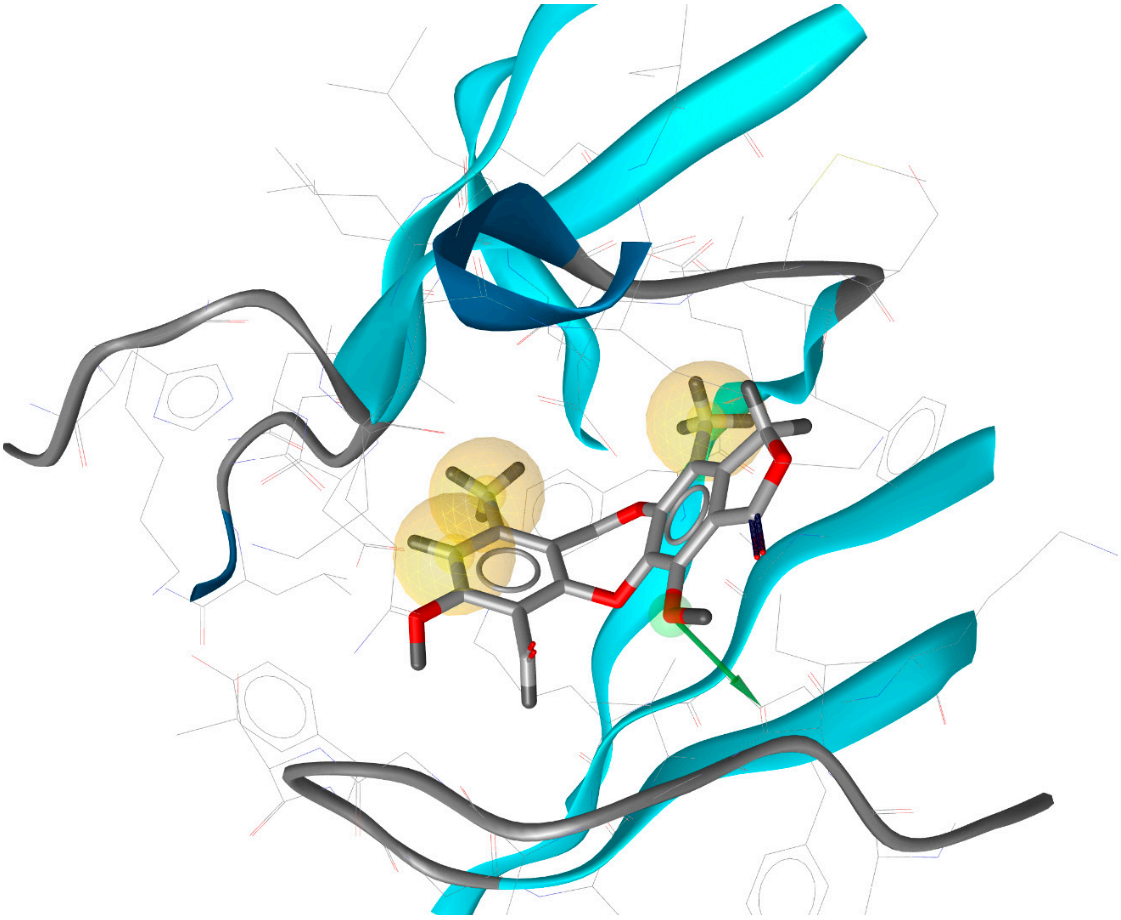

Figure S11. Pharmacophore analysis of the complex CDK1 and Cordycepsidone A in a 3D representation. This representation was generated with LigandScout 4.5 (2025), Inte:Ligand GmbH, Vienna, Austria (www.inteligand.com/ligandscout).

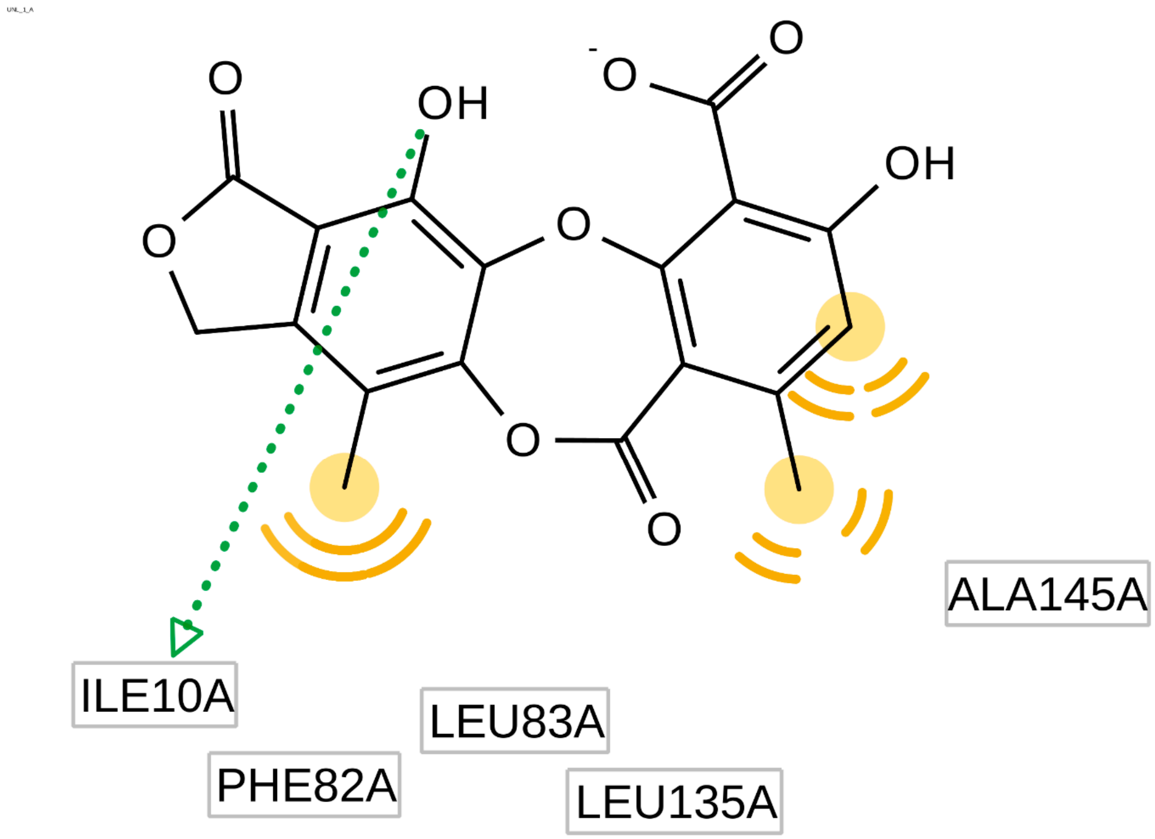

Figure S12. Pharmacophore analysis of the complex CDK1 and Cordycepsidone B in a 2D representation. This representation was generated with LigandScout 4.5 (2025), Inte:Ligand GmbH, Vienna, Austria (www.inteligand.com/ligandscout).

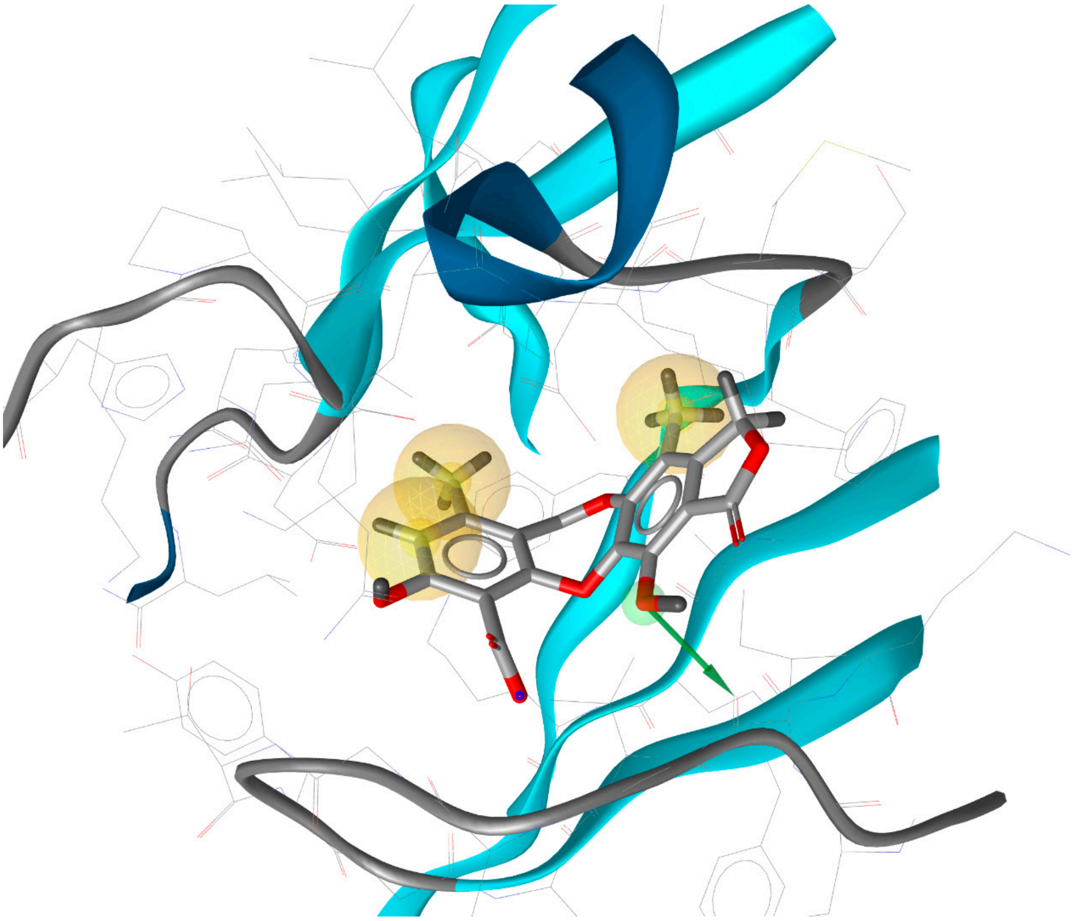

Figure S13. Pharmacophore analysis of the complex CDK1 and Cordycepsidone B in a 3D representation. This representation was generated with LigandScout 4.5 (2025), Inte:Ligand GmbH, Vienna, Austria (www.inteligand.com/ligandscout).

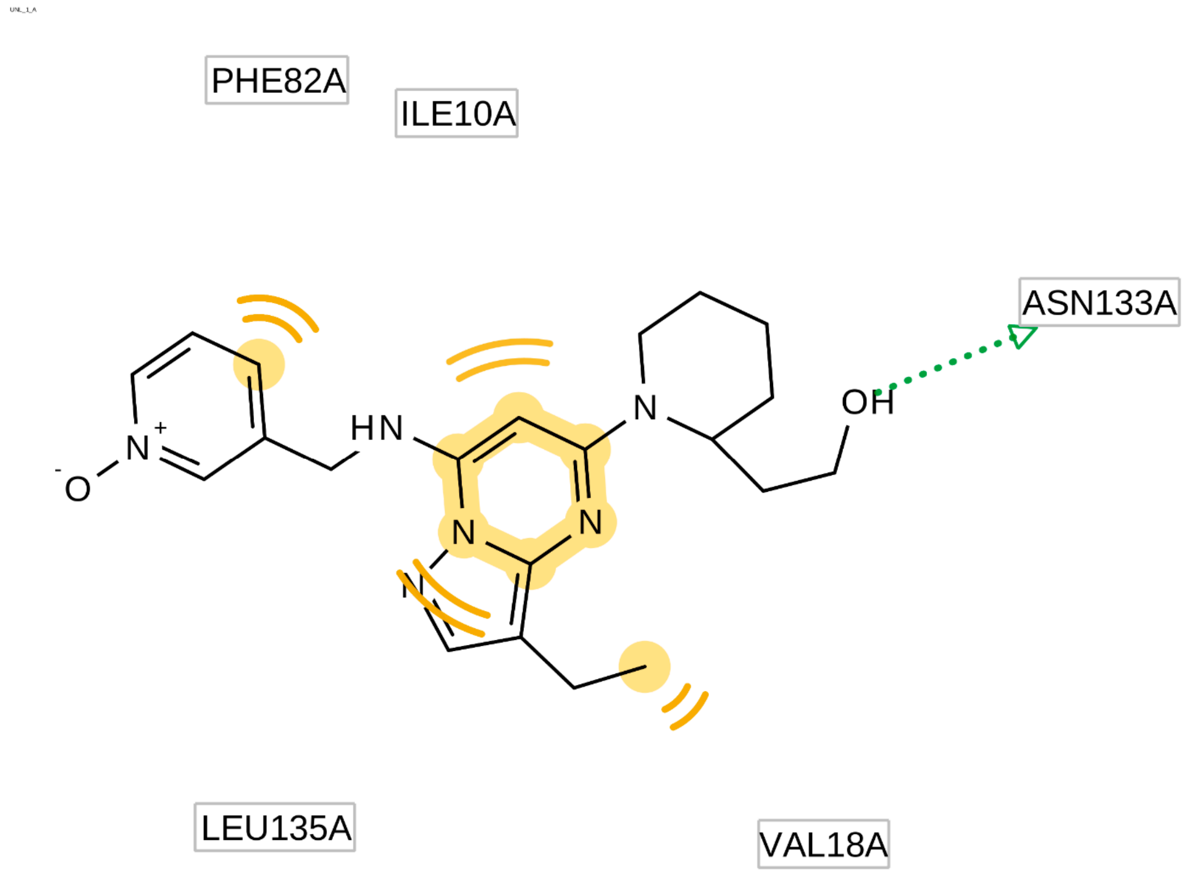

Figure S14. Pharmacophore analysis of the complex CDK1 and Dinaciclib in a 2D representation. This representation was generated with LigandScout 4.5 (2025), Inte:Ligand GmbH, Vienna, Austria (www.inteligand.com/ligandscout).

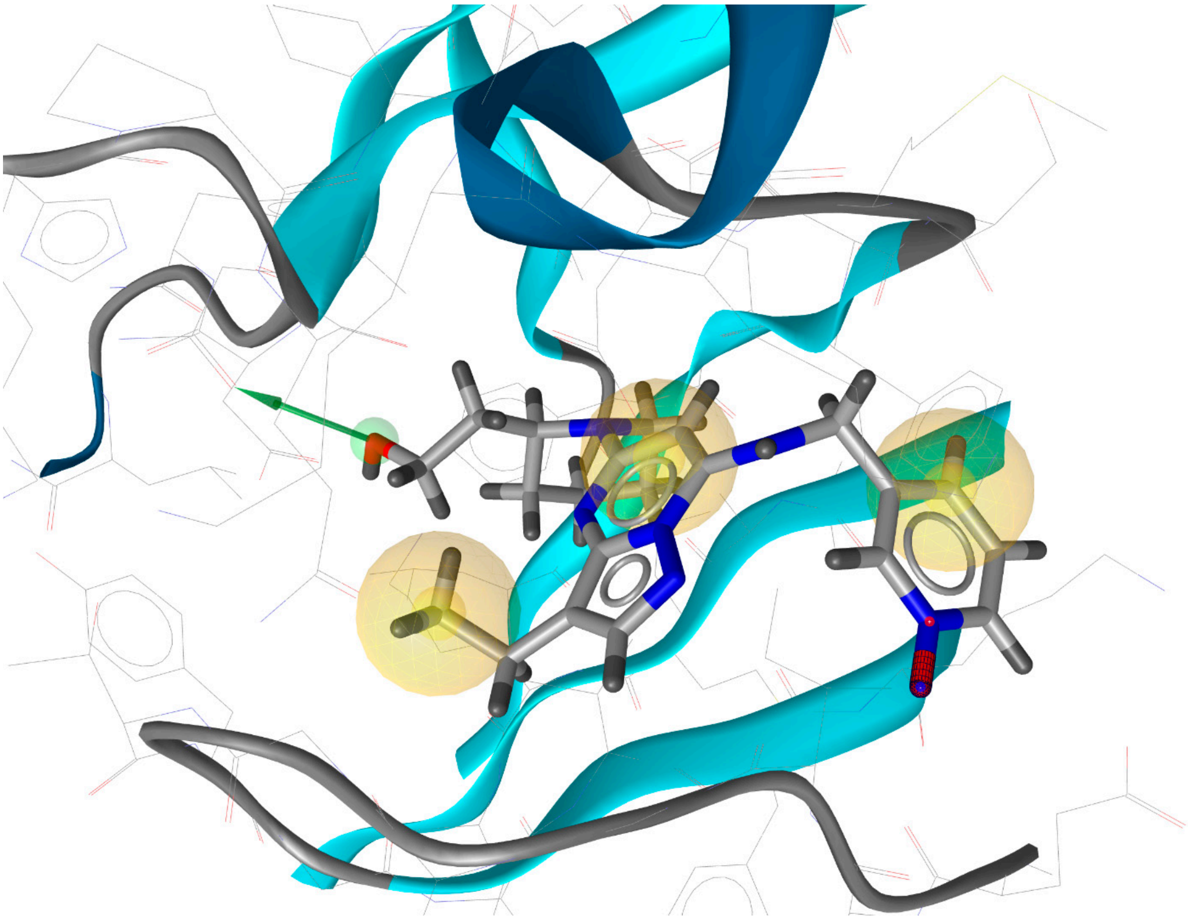

Figure S15. Pharmacophore analysis of the complex CDK1 and Dinaciclib in a 3D representation. This representation was generated with LigandScout 4.5 (2025), Inte:Ligand GmbH, Vienna, Austria (www.inteligand.com/ligandscout).

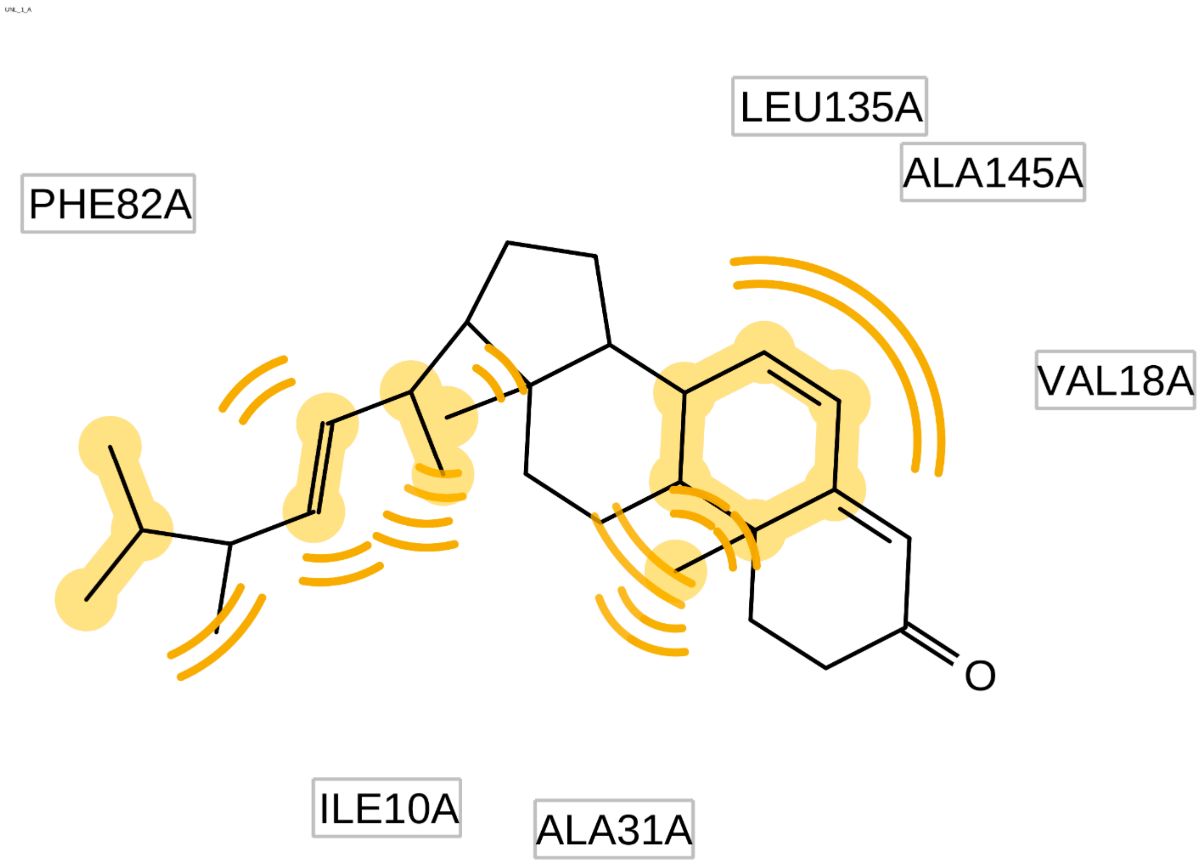

Figure S16. Pharmacophore analysis of the complex CDK1 and Ergosta-4,6,8 (14),22-tetraen-3-one in a 2D representation. This representation was generated with LigandScout 4.5 (2025), Inte:Ligand GmbH, Vienna, Austria (www.inteligand.com/ligandscout).

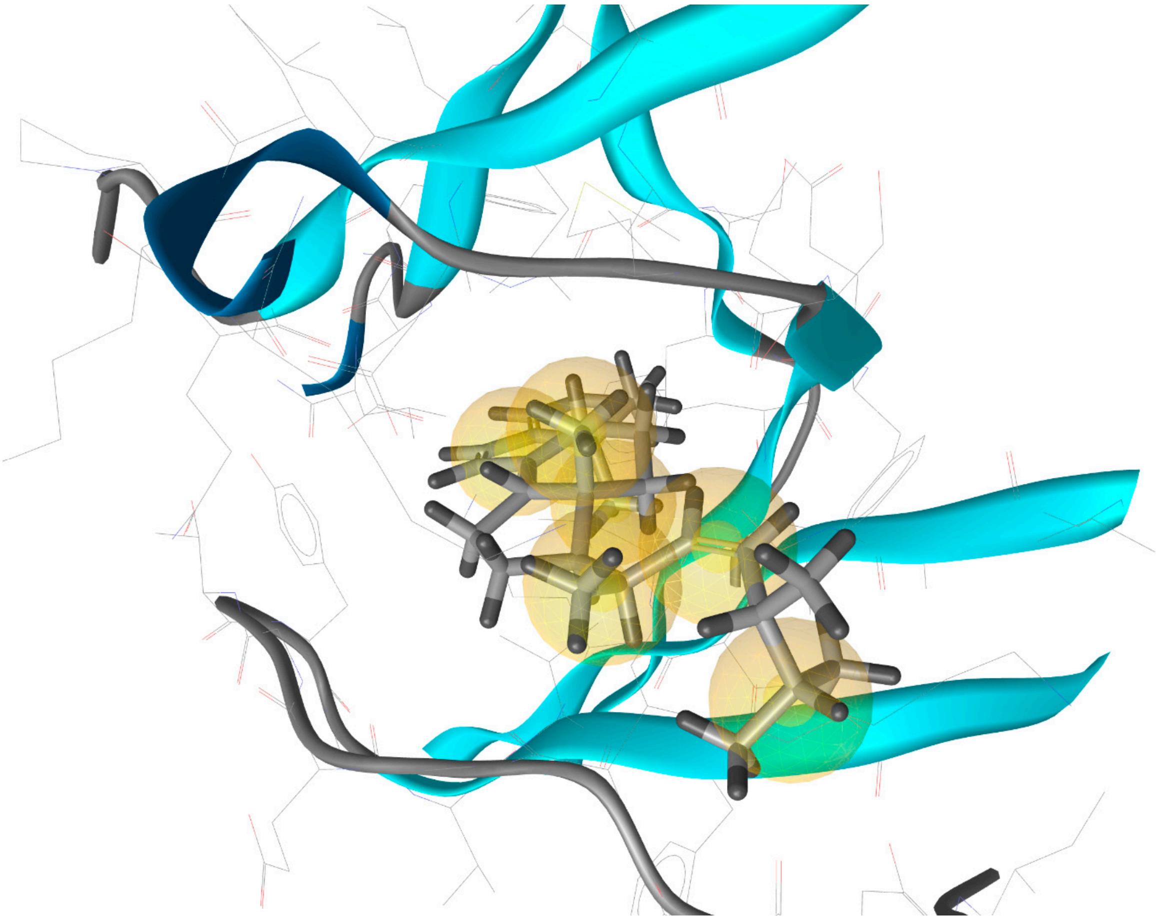

Figure S17. Pharmacophore analysis of the complex CDK1 and Ergosta-4,6,8 (14),22-tetraen-3-one in a 3D representation. This representation was generated with LigandScout 4.5 (2025), Inte:Ligand GmbH, Vienna, Austria (www.inteligand.com/ligandscout).

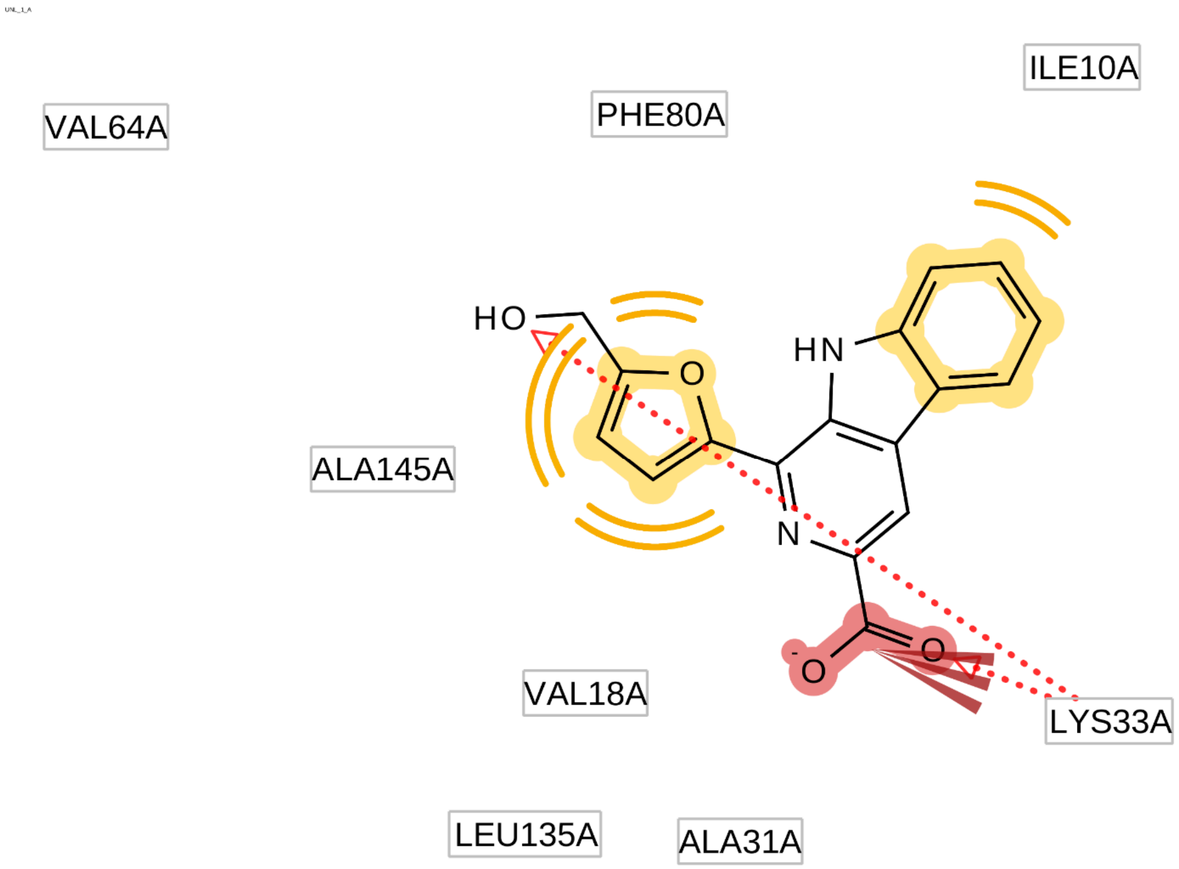

Figure S18. Pharmacophore analysis of the complex CDK1 and Flazin in a 2D representation. This representation was generated with LigandScout 4.5 (2025), Inte:Ligand GmbH, Vienna, Austria (www.inteligand.com/ligandscout).

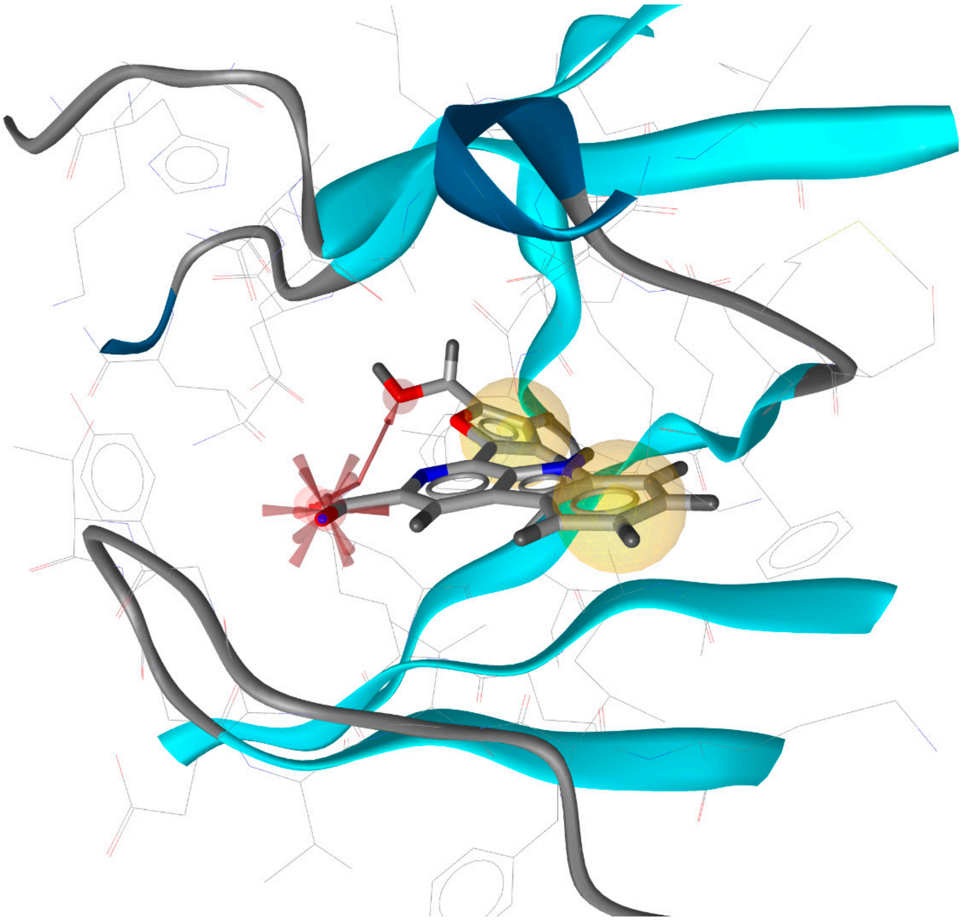

Figure S19. Pharmacophore analysis of the complex CDK1 and Flazin in a 3D representation. This representation was generated with LigandScout 4.5 (2025), Inte:Ligand GmbH, Vienna, Austria (www.inteligand.com/ligandscout).

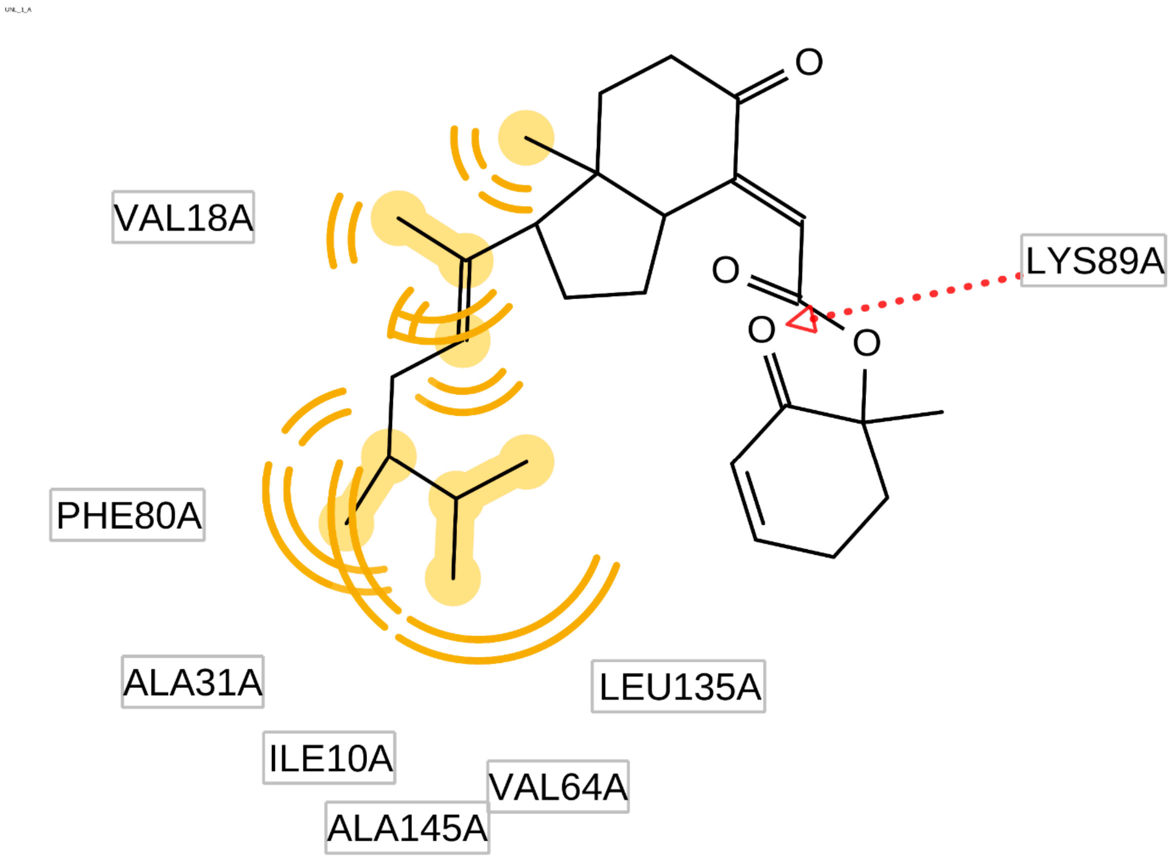

Figure S20. Pharmacophore analysis of the complex CDK1 and Jiangxienone in a 2D representation. This representation was generated with LigandScout 4.5 (2025), Inte:Ligand GmbH, Vienna, Austria (www.inteligand.com/ligandscout).

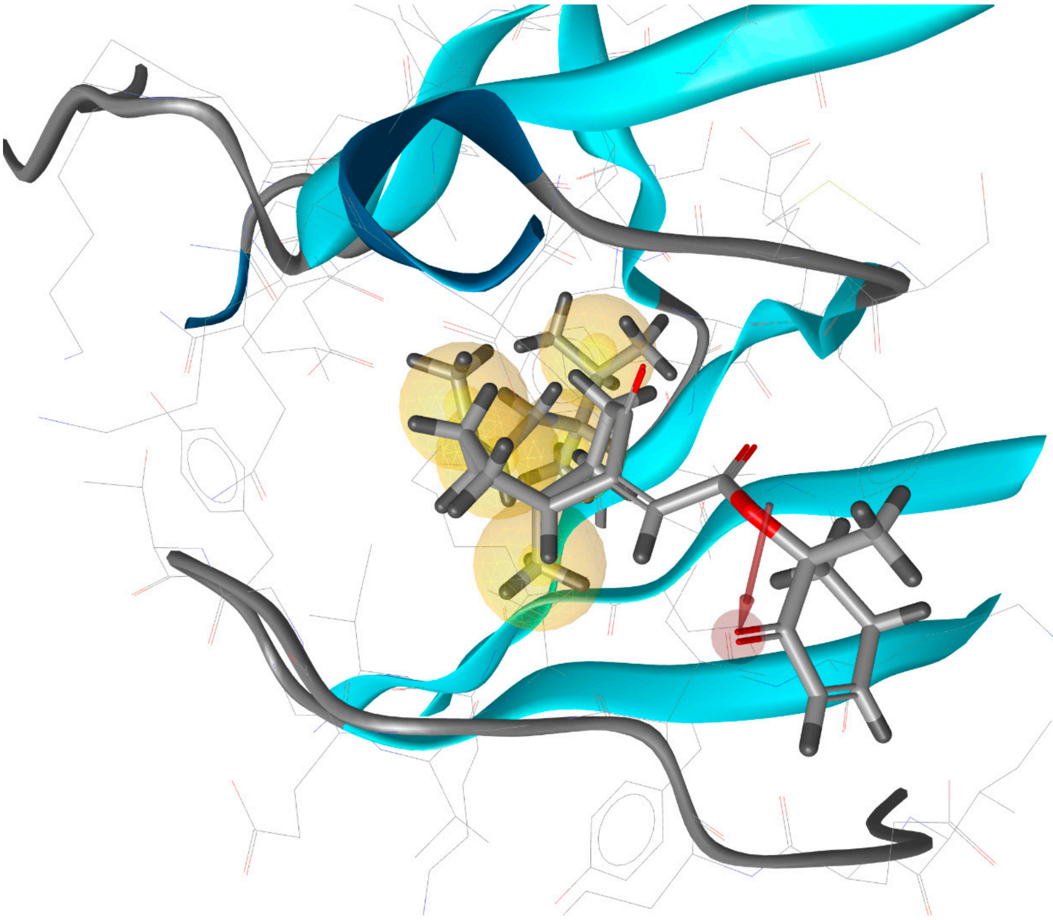

Figure S21. Pharmacophore analysis of the complex CDK1 and Jiangxienone in a 3D representation. This representation was generated with LigandScout 4.5 (2025), Inte:Ligand GmbH, Vienna, Austria (www.inteligand.com/ligandscout).

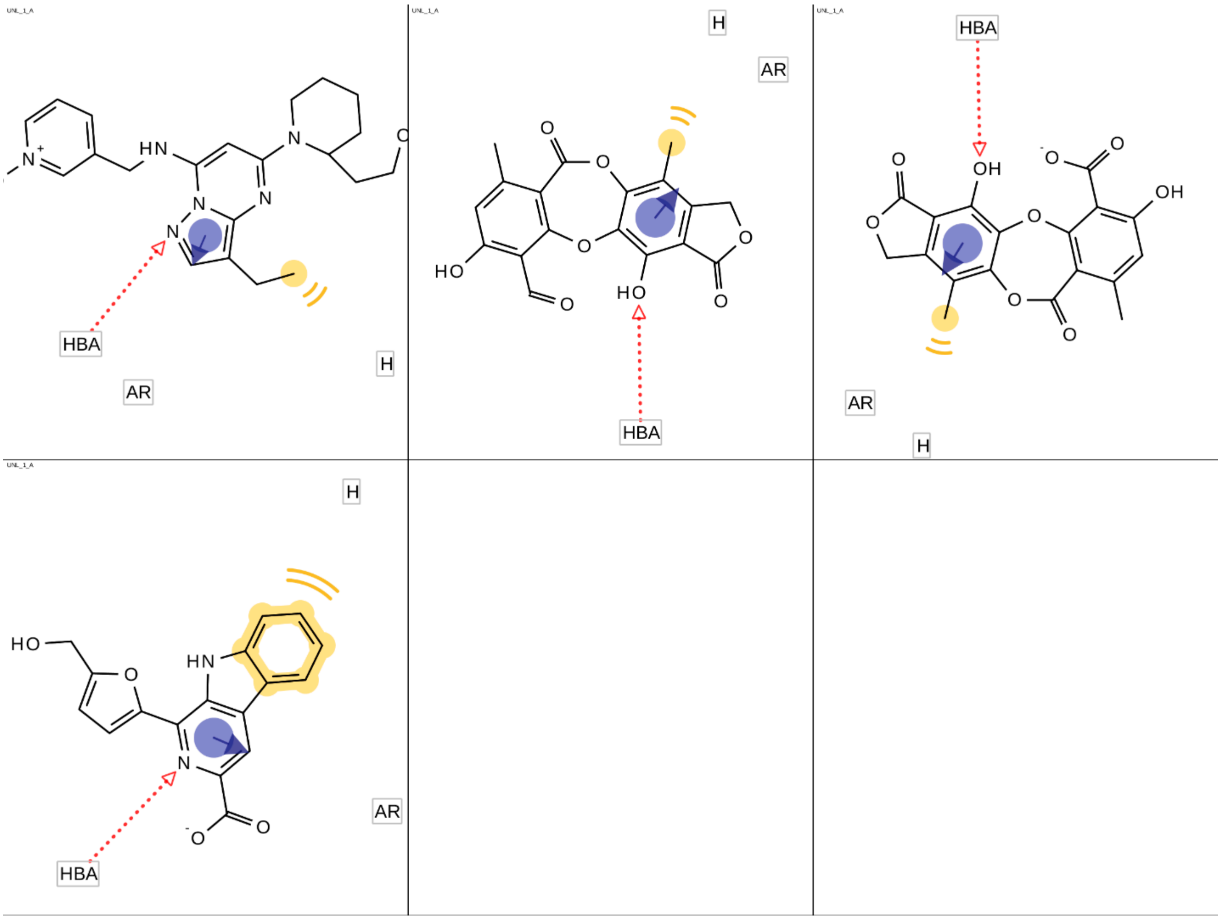

Figure S22. Alignment analysis that shows the shared pharmacophore elements of Dinaciclib, Cordycepsidone A, Cordycepsidone B, and Flazin in a 2D representation. This representation was generated with LigandScout 4.5 (2025), Inte:Ligand GmbH, Vienna, Austria (www.inteligand.com/ligandscout).

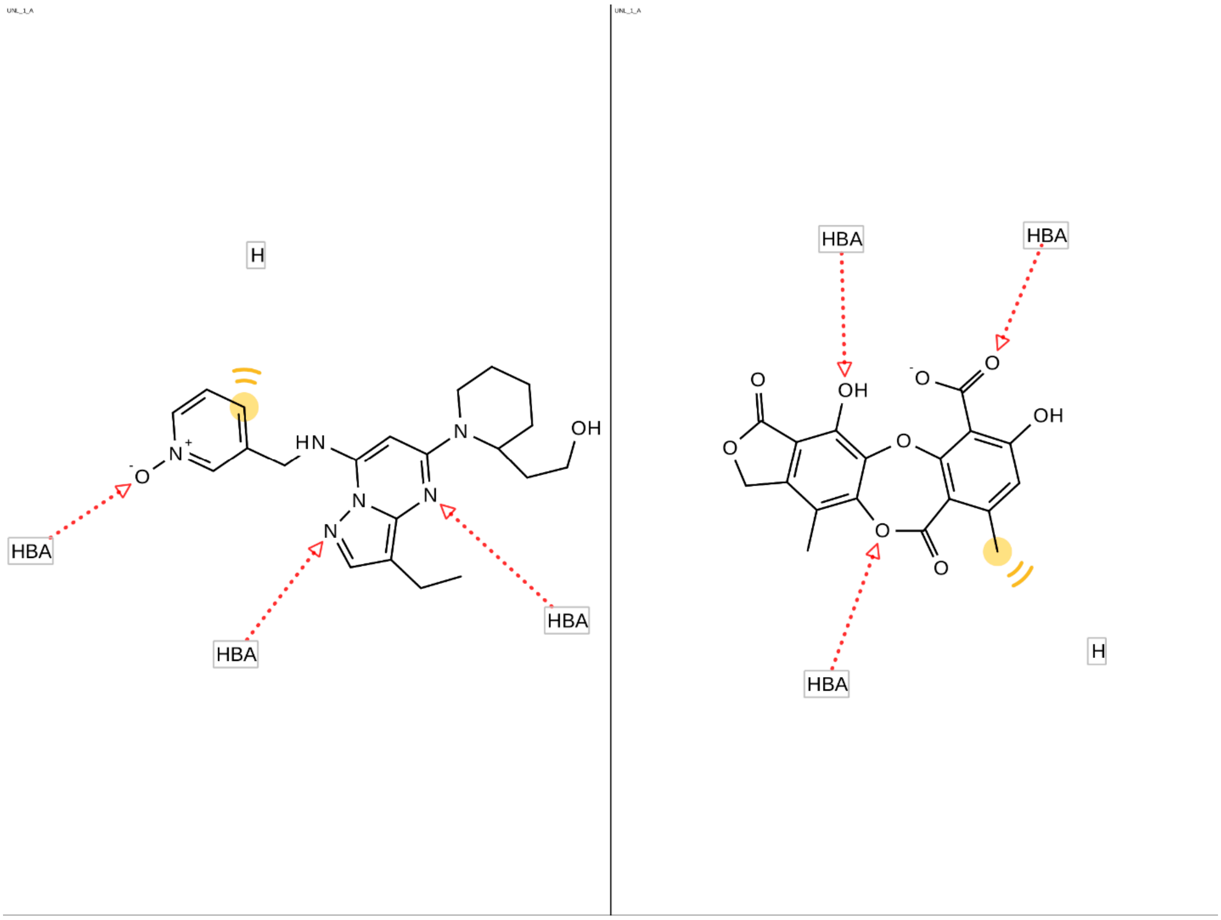

Figure S23. Alignment analysis that shows the shared pharmacophore elements of Dinaciclib and Cordycepsidone B in a 2D representation. This representation was generated with LigandScout 4.5 (2025), Inte:Ligand GmbH, Vienna, Austria (www.inteligand.com/ligandscout).

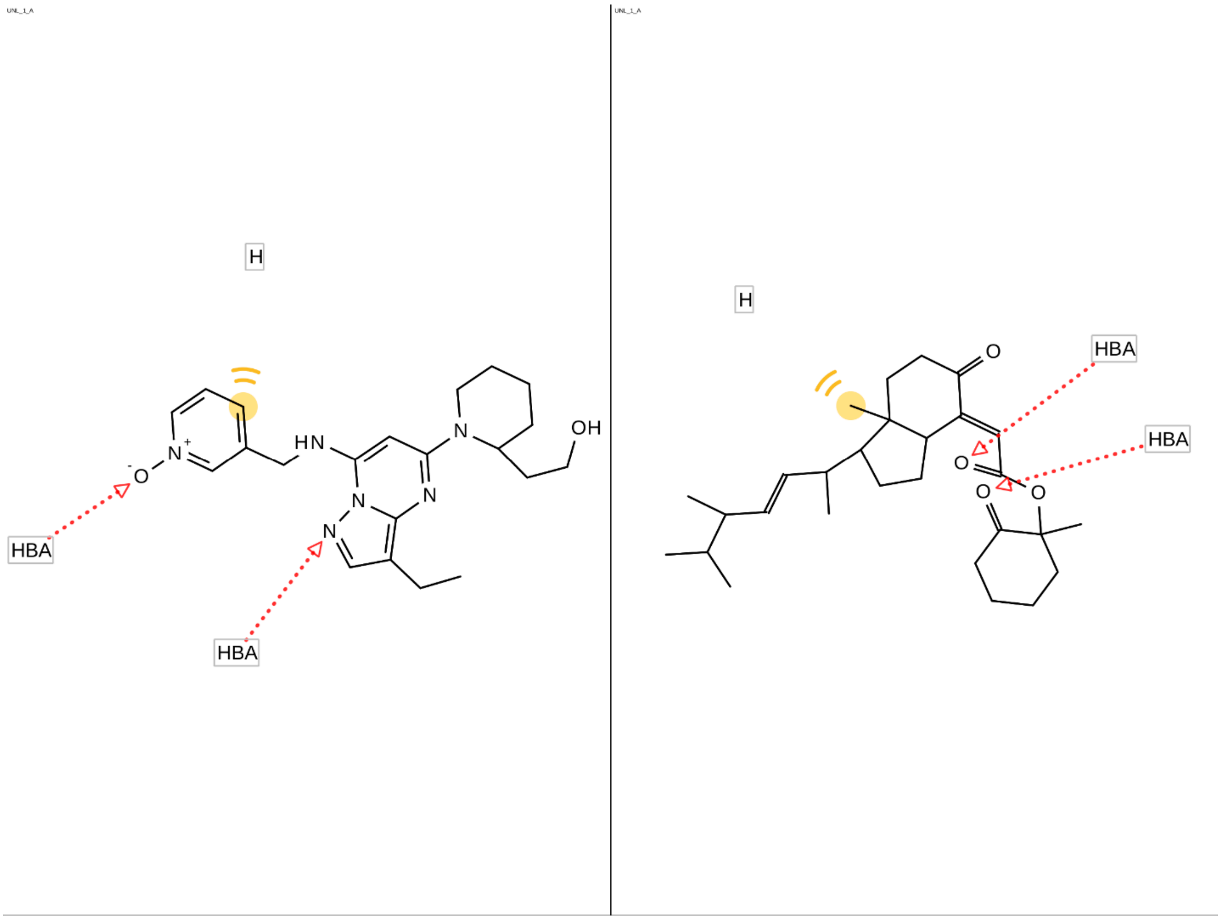

Figure S24. Alignment analysis that shows the shared pharmacophore elements of Dinaciclib and Jiangxienone in a 2D representation. This representation was generated with LigandScout 4.5 (2025), Inte:Ligand GmbH, Vienna, Austria (www.inteligand.com/ligandscout).

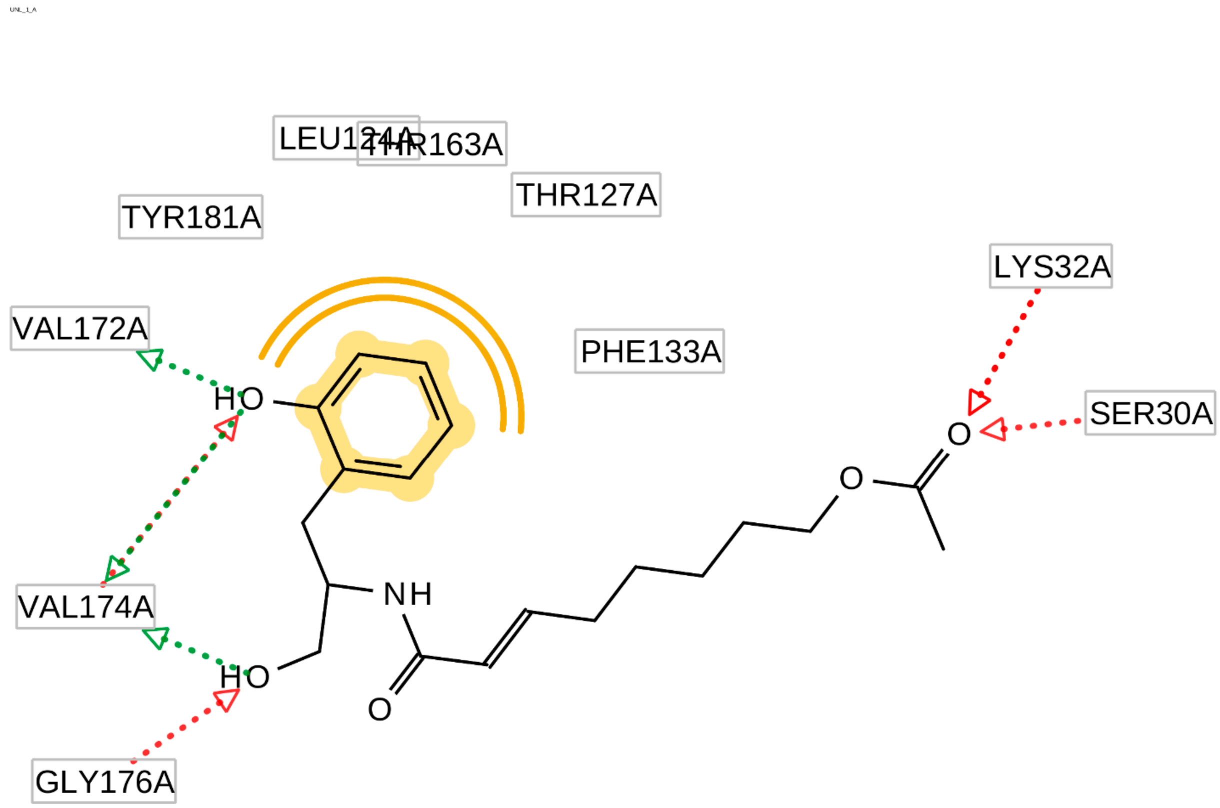

Figure S25. Pharmacophore analysis of the complex TK-1 and Cordycepiamide C in a 2D representation. This representation was generated with LigandScout 4.5 (2025), Inte:Ligand GmbH, Vienna, Austria (www.inteligand.com/ligandscout).

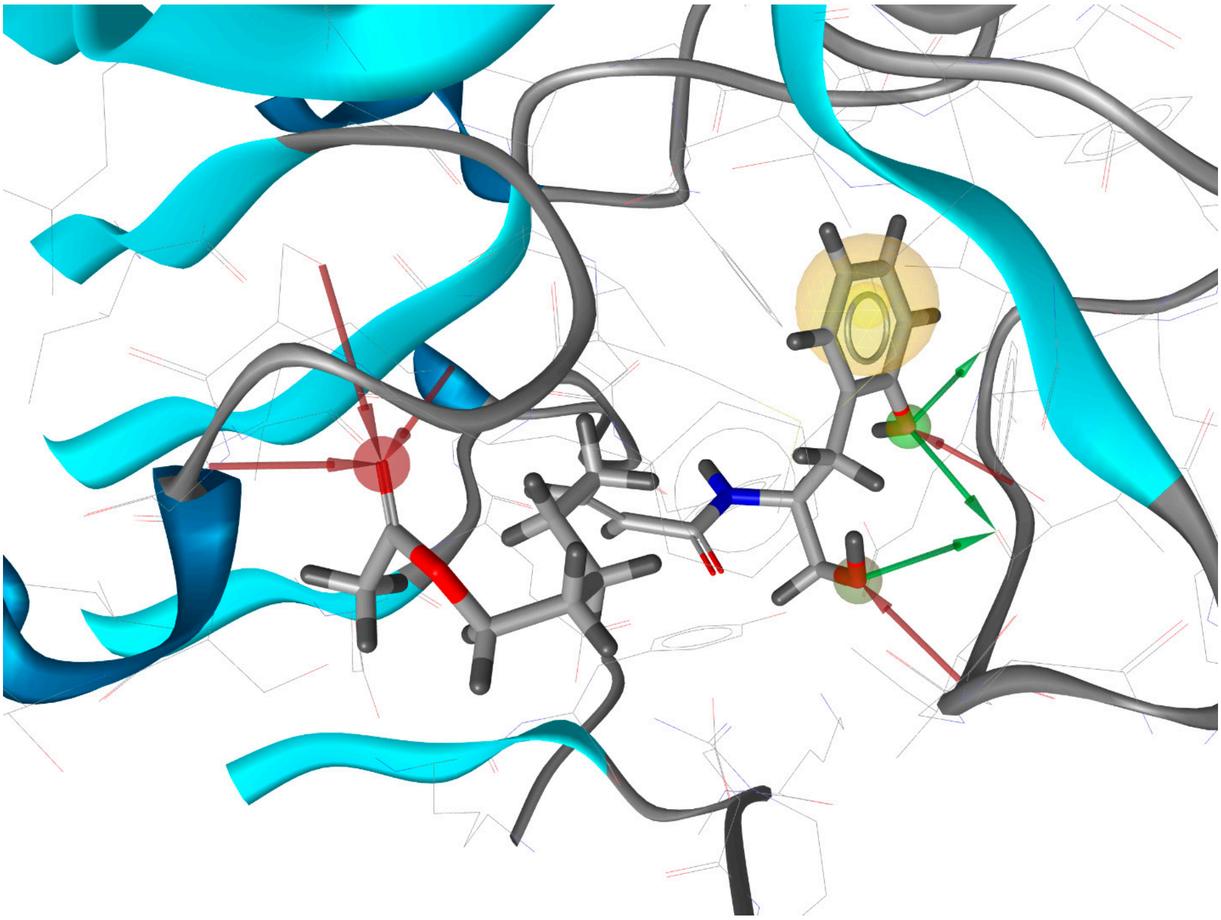

Figure S26. Pharmacophore analysis of the complex TK-1 and Cordycepiamide C in a 3D representation. This representation was generated with LigandScout 4.5 (2025), Inte:Ligand GmbH, Vienna, Austria (www.inteligand.com/ligandscout).

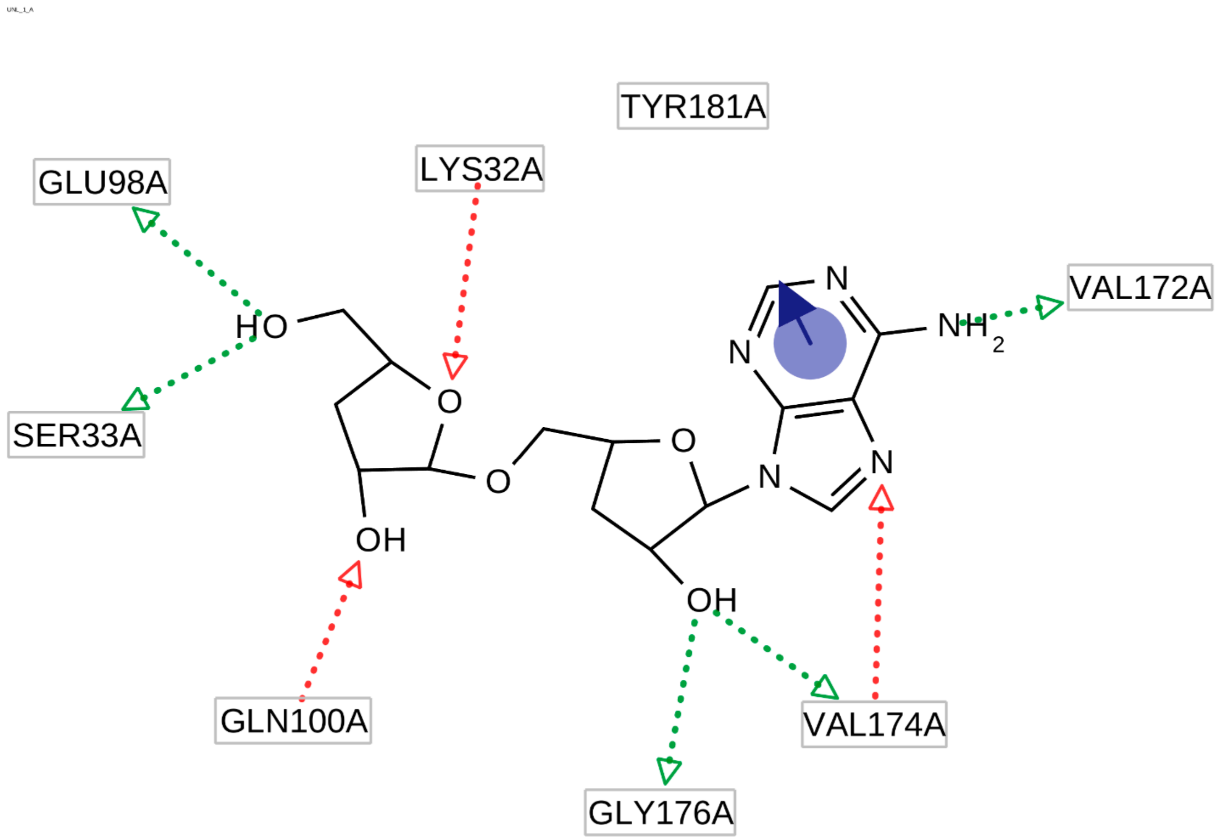

Figure S27. Pharmacophore analysis of the complex TK-1 and 5′-(3′′-Deoxy-β-D-ribofuranosyl)-3′-deoxyadenosine in a 2D representation. This representation was generated with LigandScout 4.5 (2025), Inte:Ligand GmbH, Vienna, Austria (www.inteligand.com/ligandscout).

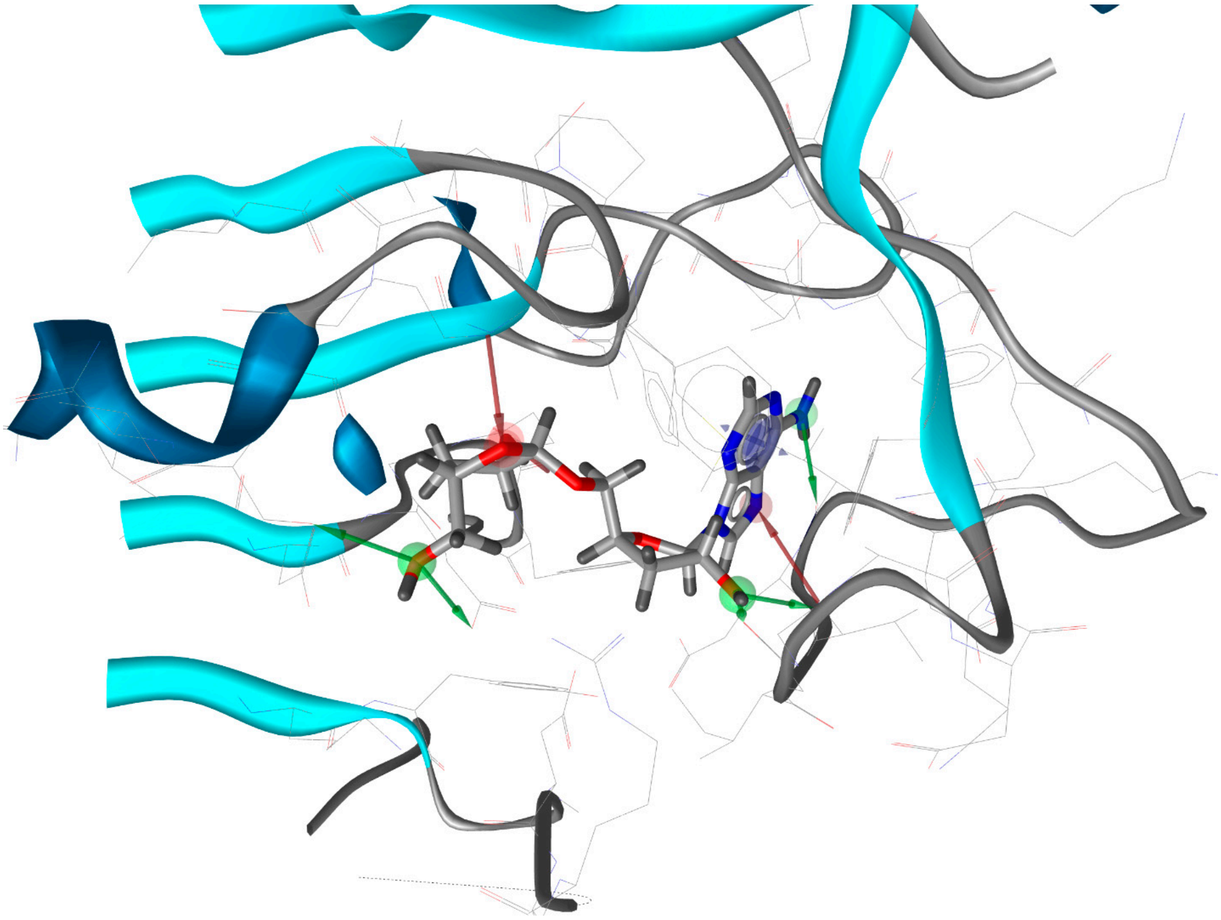

Figure S28. Pharmacophore analysis of the complex TK-1 and 5′-(3′′-Deoxy-β-D-ribofuranosyl)-3′-deoxyadenosine in a 3D representation. This representation was generated with LigandScout 4.5 (2025), Inte:Ligand GmbH, Vienna, Austria (www.inteligand.com/ligandscout).

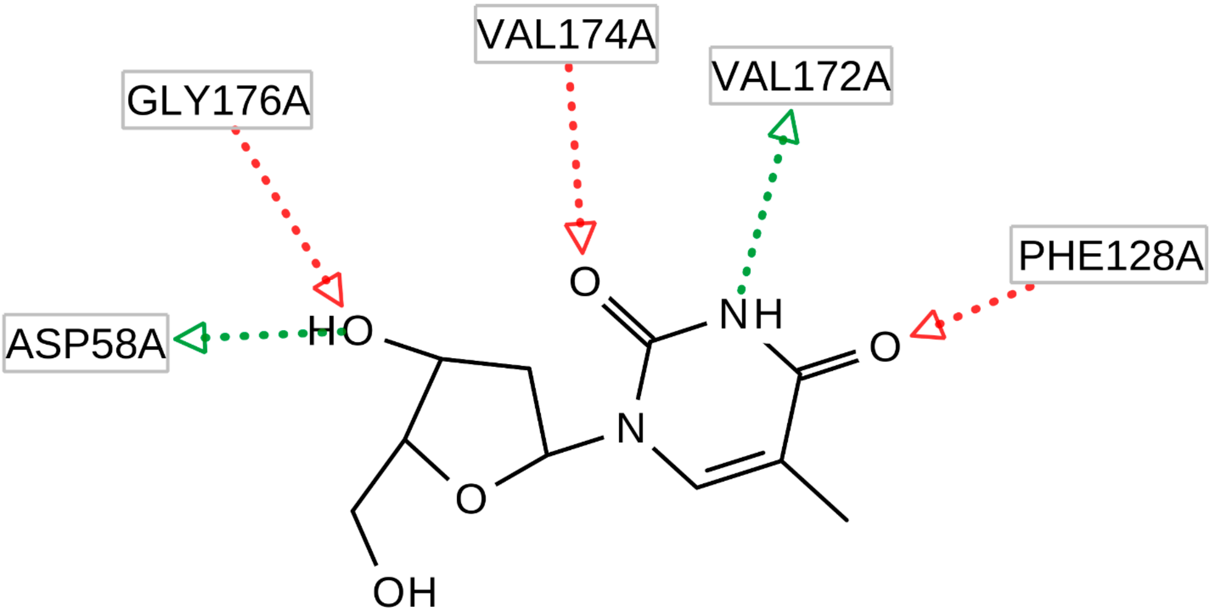

Figure S29. Pharmacophore analysis of the complex TK-1 and Thymidine in a 2D representation. This representation was generated with LigandScout 4.5 (2025), Inte:Ligand GmbH, Vienna, Austria (www.inteligand.com/ligandscout).

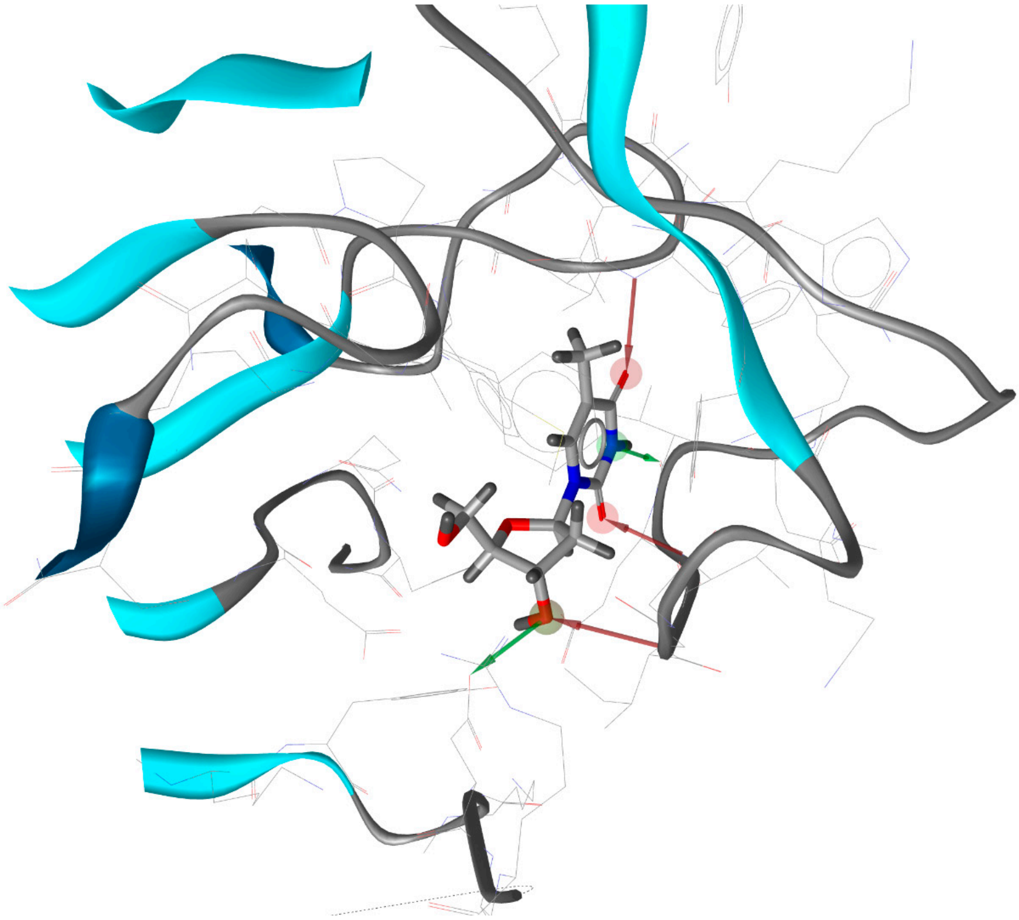

Figure S30. Pharmacophore analysis of the complex TK-1 and Thymidine in a 3D representation. This representation was generated with LigandScout 4.5 (2025), Inte:Ligand GmbH, Vienna, Austria (www.inteligand.com/ligandscout).

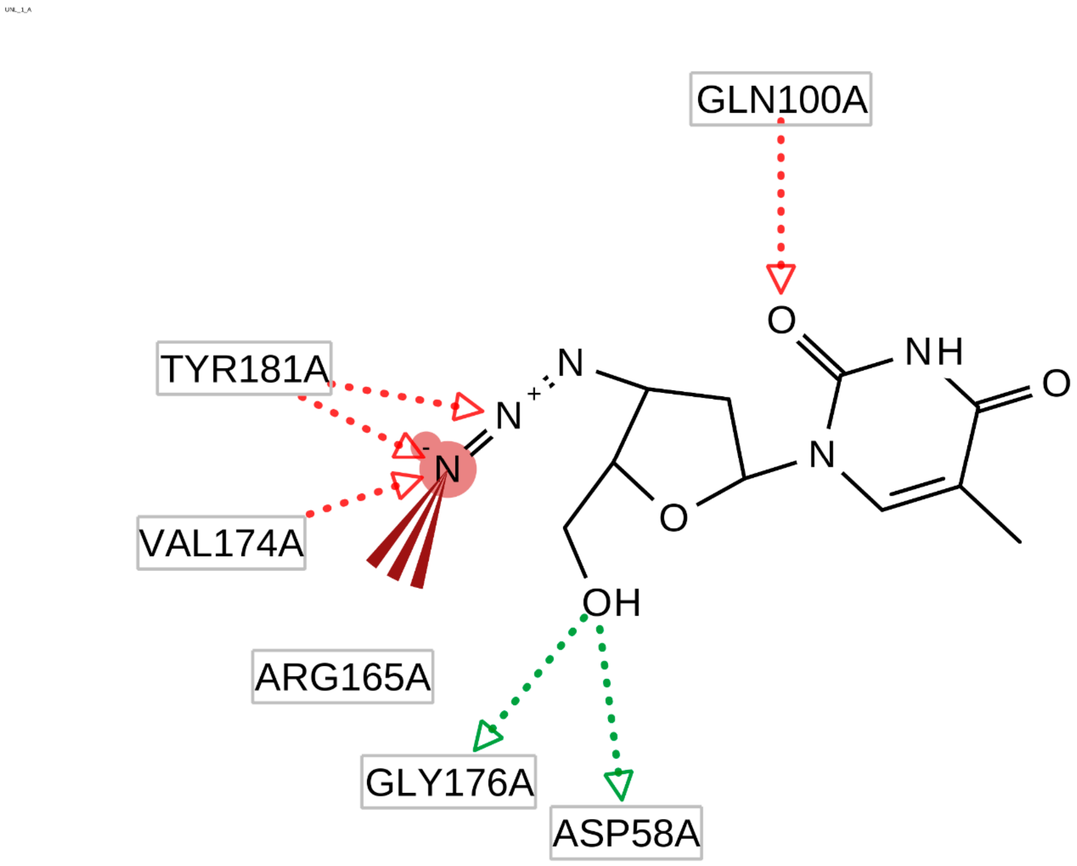

Figure S31. Pharmacophore analysis of the complex TK-1 and Zidovudine in a 2D representation. This representation was generated with LigandScout 4.5 (2025), Inte:Ligand GmbH, Vienna, Austria (www.inteligand.com/ligandscout).

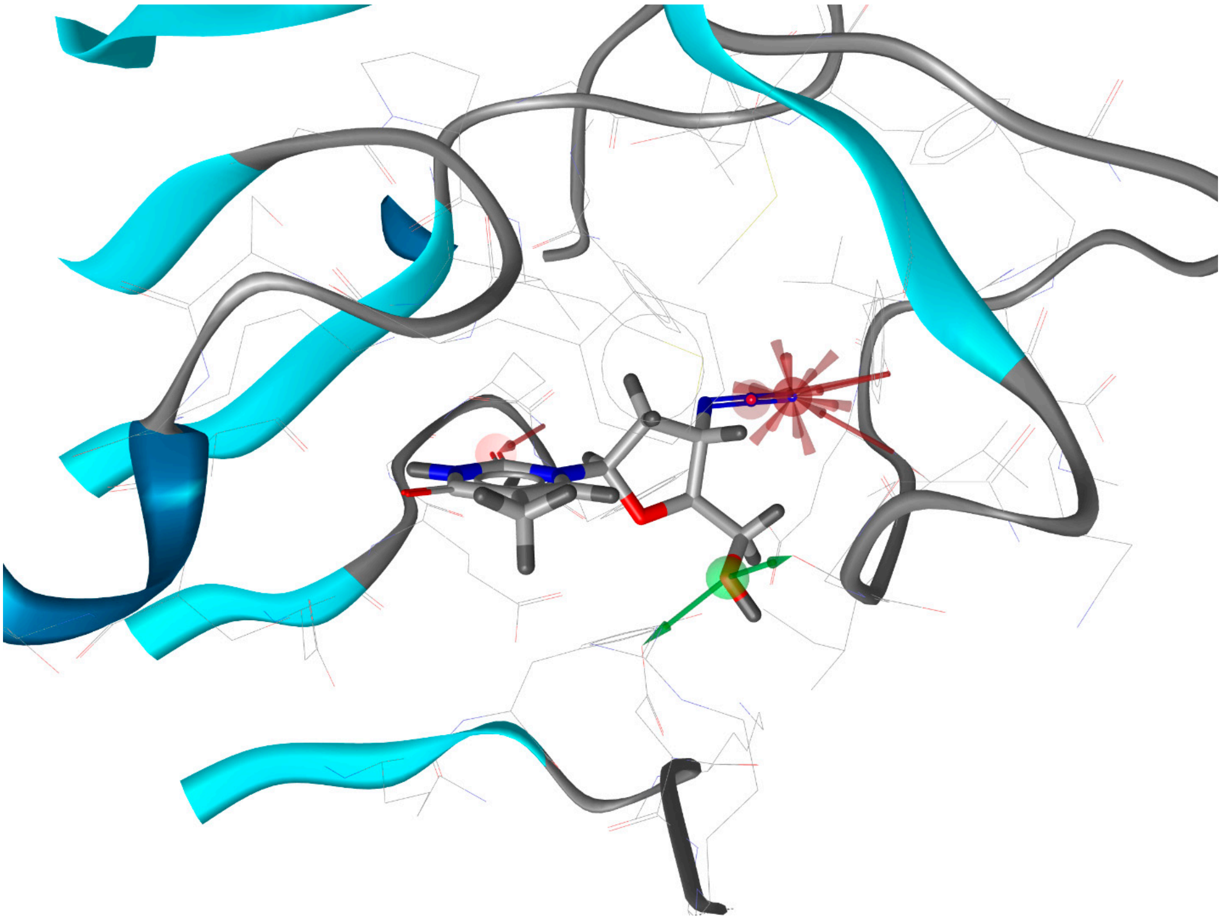

Figure S32. Pharmacophore analysis of the complex TK-1 and Zidovudine in a 3D representation. This representation was generated with LigandScout 4.5 (2025), Inte:Ligand GmbH, Vienna, Austria (www.inteligand.com/ligandscout).

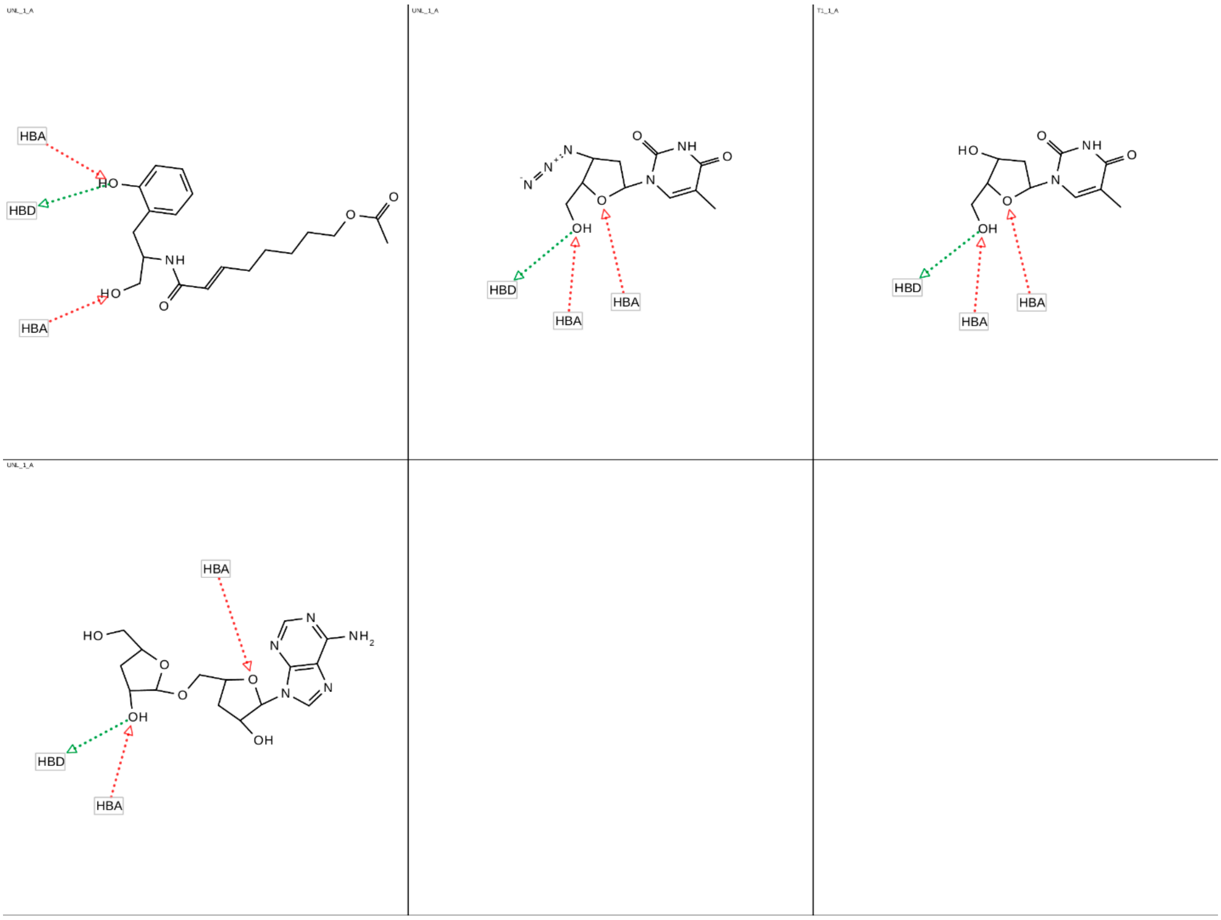

Figure S33. Alignment analysis that shows the shared pharmacophore elements of Cordycepiamide C, Zidovudine, Thymidine, and 5′-(3′′-Deoxy-β-D-ribofuranosyl)-3′-deoxyadenosine in a 2D representation. This representation was generated with LigandScout 4.5 (2025), Inte:Ligand GmbH, Vienna, Austria (www.inteligand.com/ligandscout).

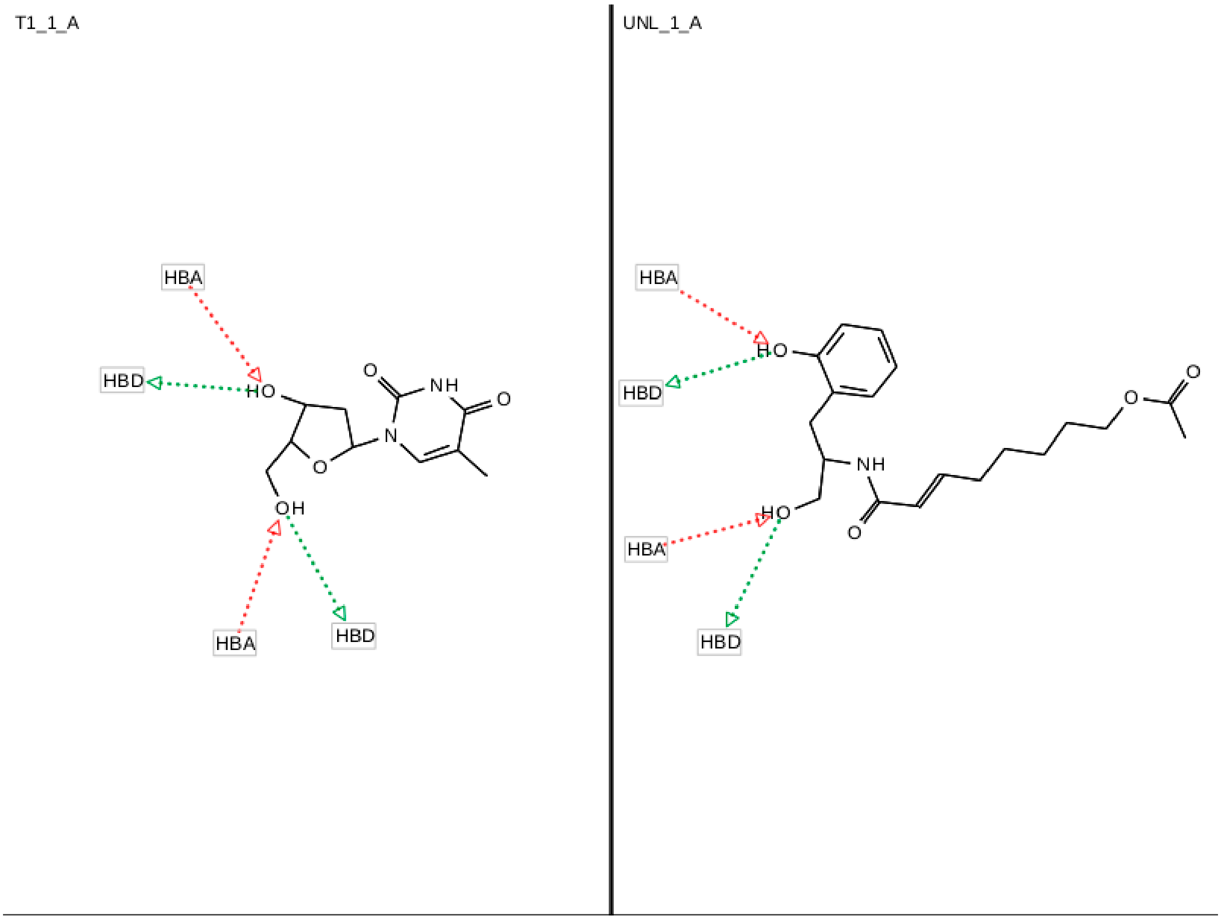

Figure S34. Alignment analysis that shows the shared pharmacophore elements of Cordycepiamide C and Thymidine in a 2D representation. This representation was generated with LigandScout 4.5 (2025), Inte:Ligand GmbH, Vienna, Austria (www.inteligand.com/ligandscout).

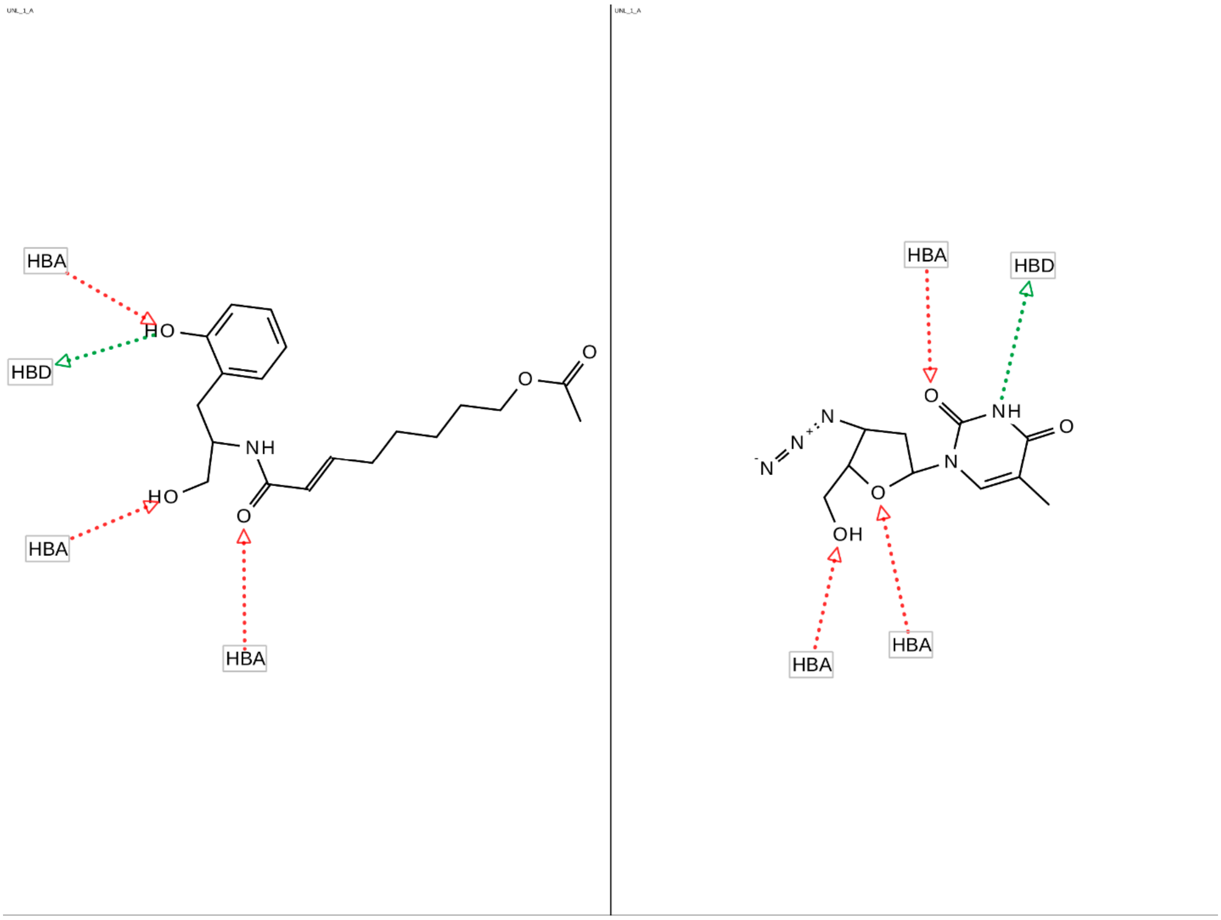

Figure S35. Alignment analysis that shows the shared pharmacophore elements of Cordycepiamide C and Zidovudine in a 2D representation. This representation was generated with LigandScout 4.5 (2025), Inte:Ligand GmbH, Vienna, Austria (www.inteligand.com/ligandscout).

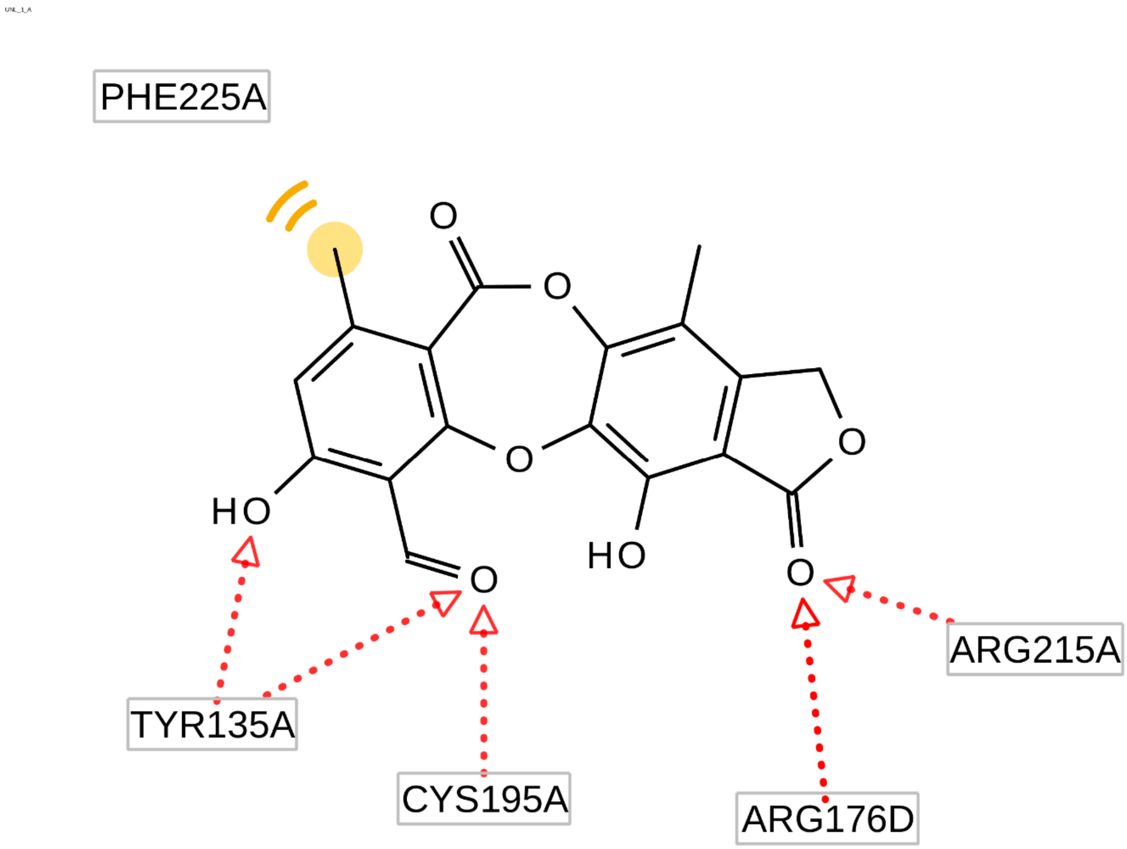

Figure S36. Pharmacophore analysis of the complex TYMS and Cordycepsidone A in a 2D representation. This representation was generated with LigandScout 4.5 (2025), Inte:Ligand GmbH, Vienna, Austria (www.inteligand.com/ligandscout).

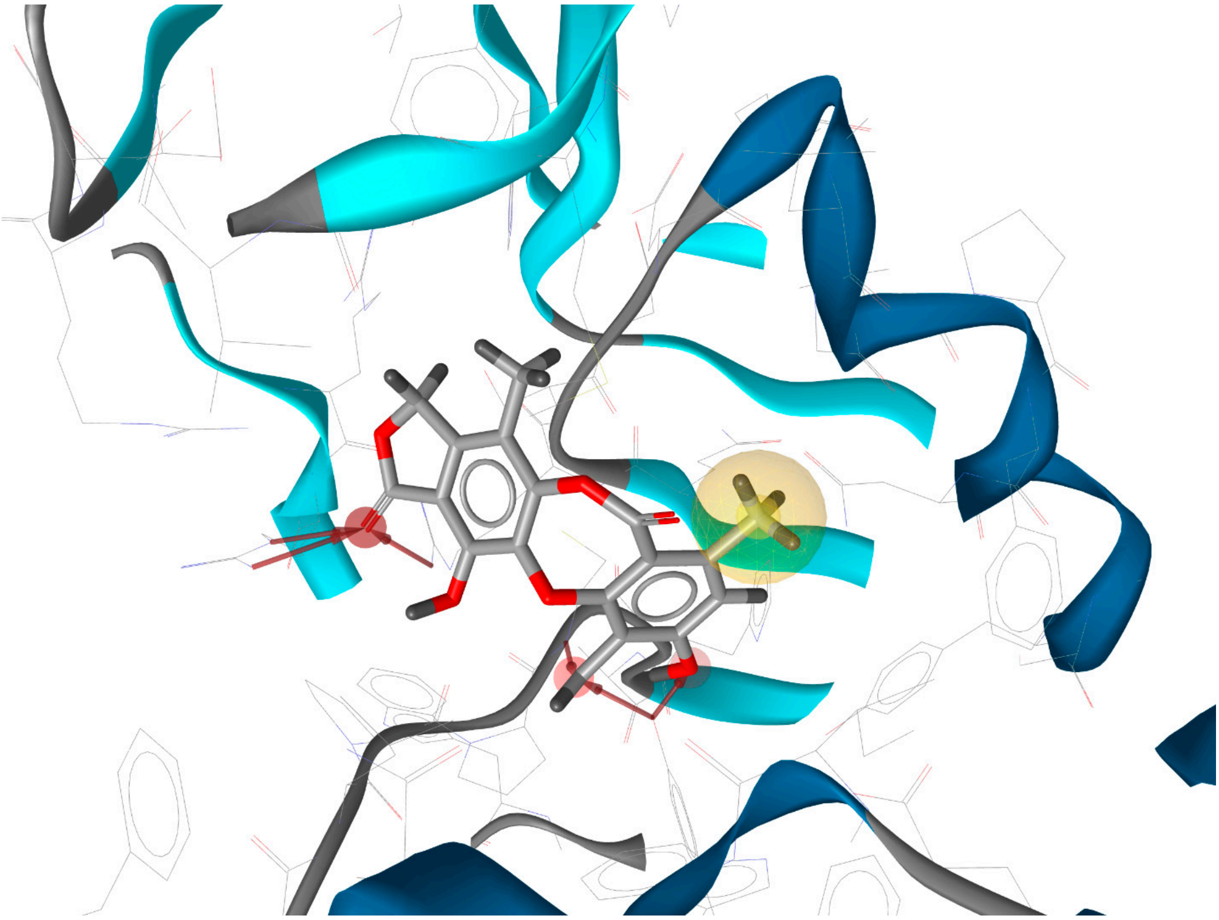

Figure S37. Pharmacophore analysis of the complex TYMS and Cordycepsidone A in a 3D representation. This representation was generated with LigandScout 4.5 (2025), Inte:Ligand GmbH, Vienna, Austria (www.inteligand.com/ligandscout).

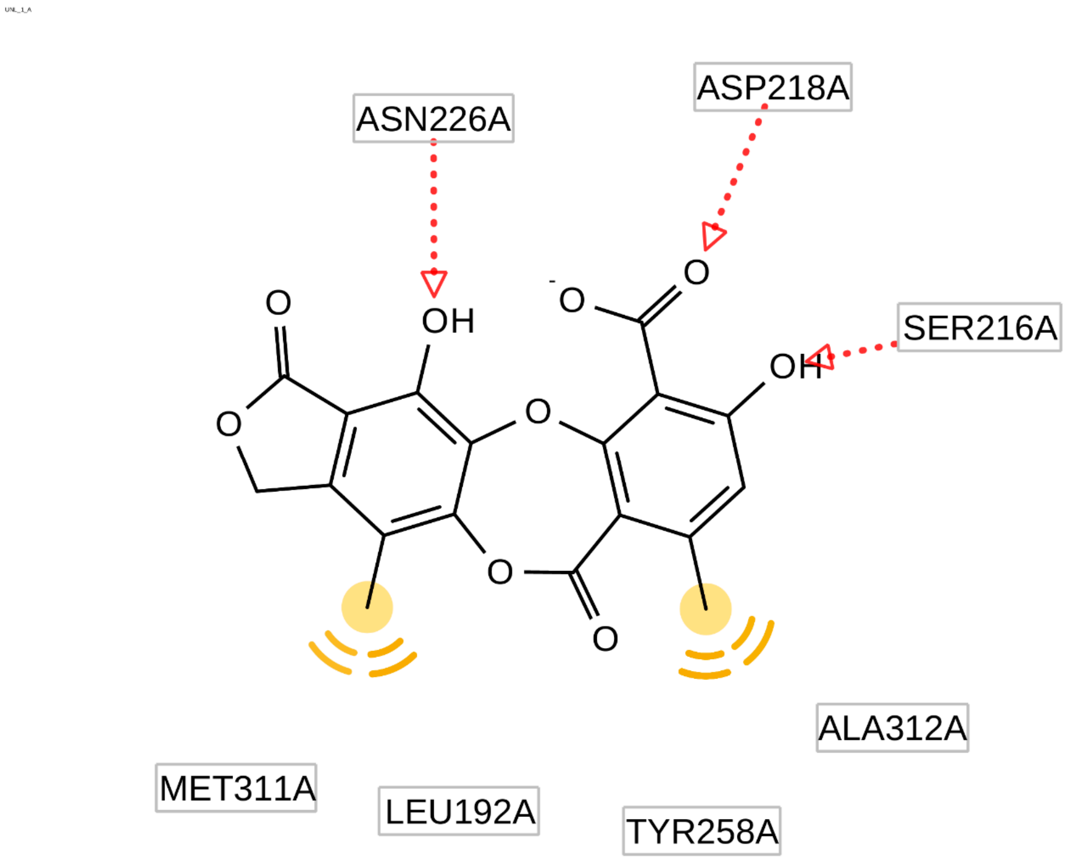

Figure S38. Pharmacophore analysis of the complex TYMS and Cordycepsidone B in a 2D representation. This representation was generated with LigandScout 4.5 (2025), Inte:Ligand GmbH, Vienna, Austria (www.inteligand.com/ligandscout).

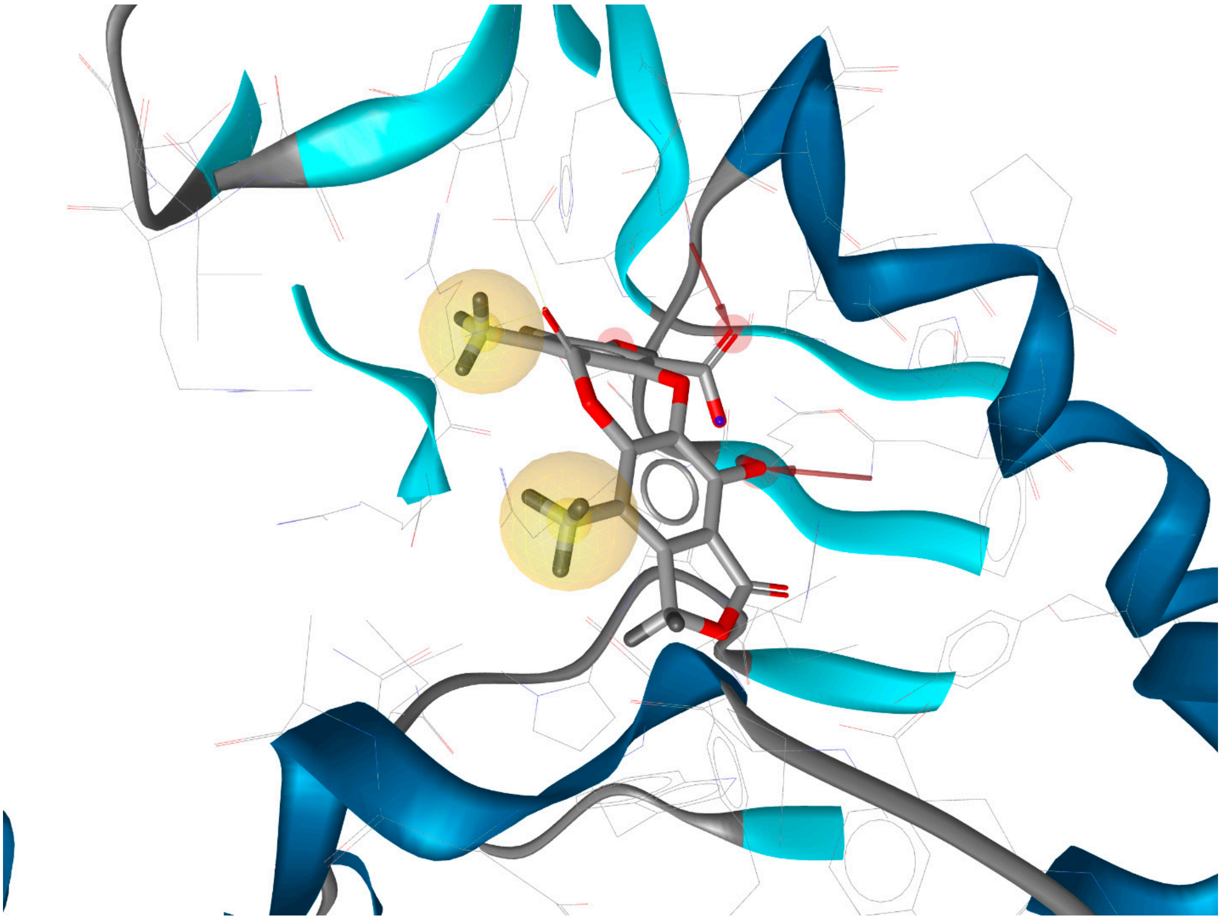

Figure S39. Pharmacophore analysis of the complex TYMS and Cordycepsidone B in a 3D representation. This representation was generated with LigandScout 4.5 (2025), Inte:Ligand GmbH, Vienna, Austria (www.inteligand.com/ligandscout).

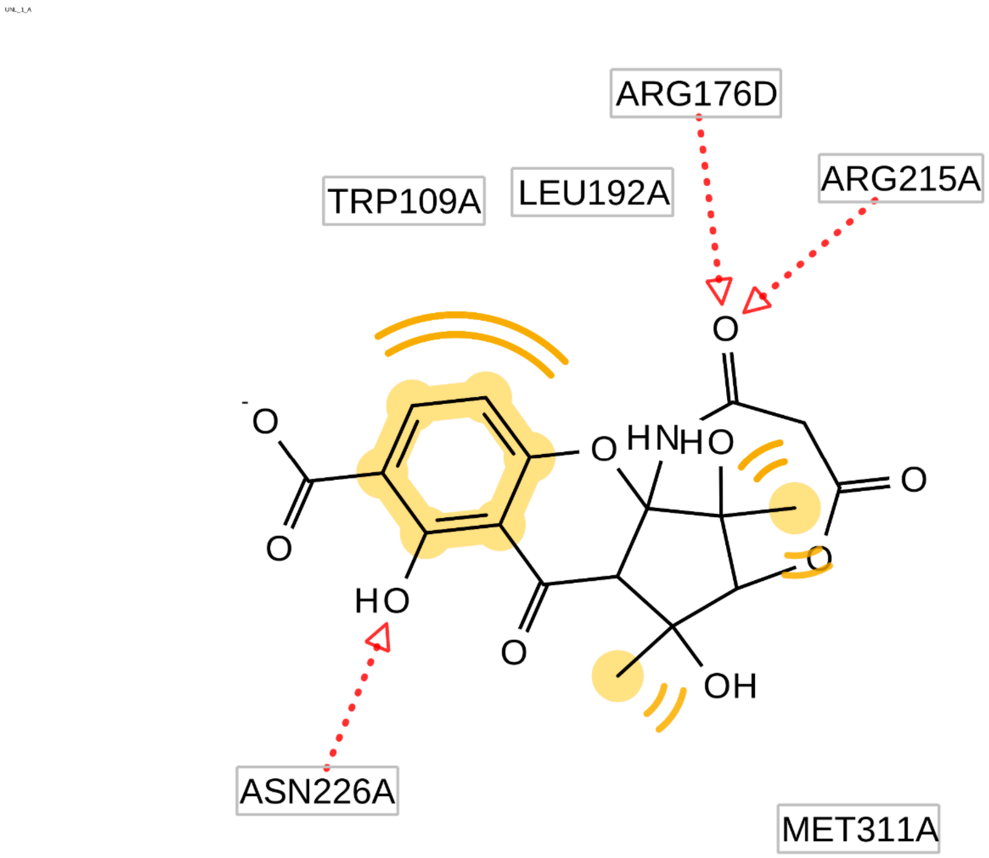

Figure S40. Pharmacophore analysis of the complex TYMS and Cryptosporioptide A in a 2D representation. This representation was generated with LigandScout 4.5 (2025), Inte:Ligand GmbH, Vienna, Austria (www.inteligand.com/ligandscout).

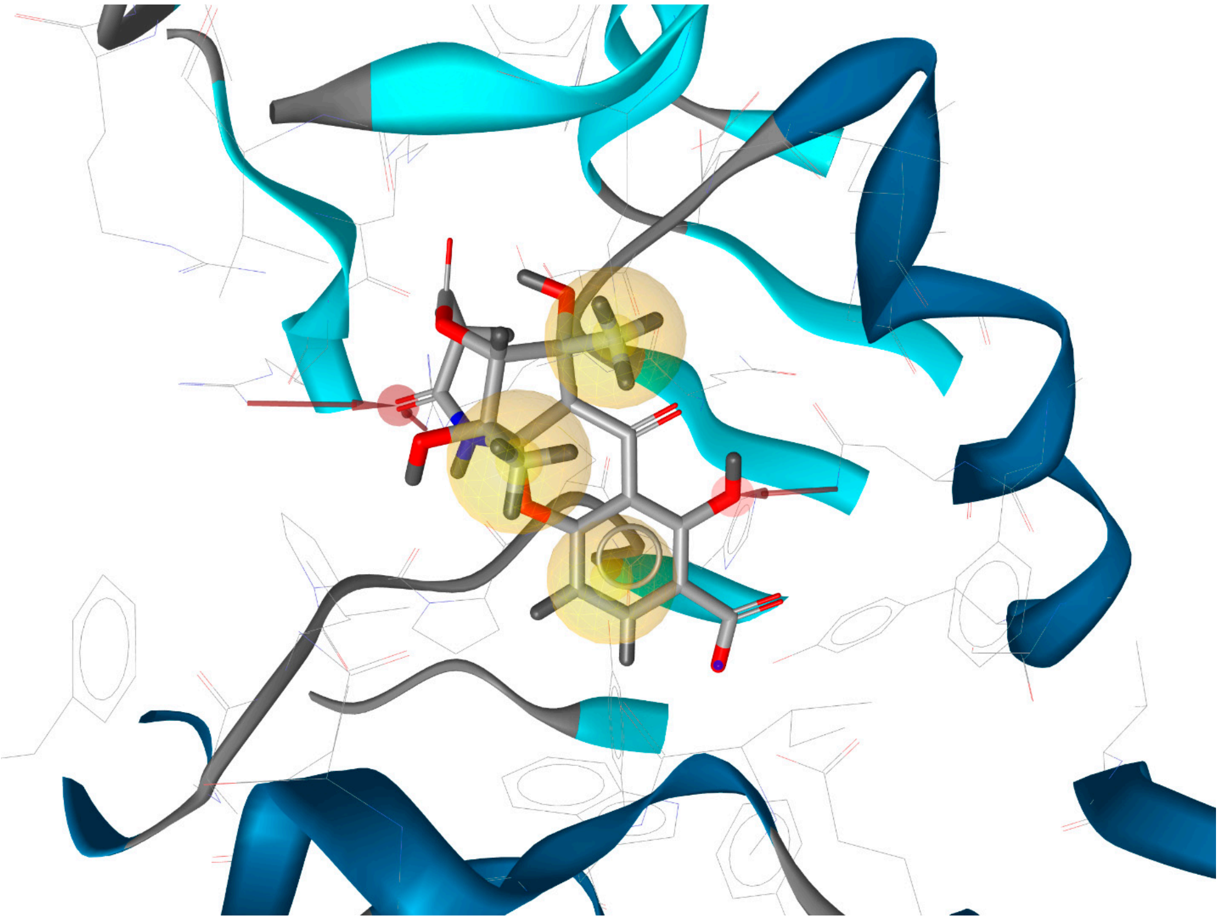

Figure S41. Pharmacophore analysis of the complex TYMS and Cryptosporioptide A in a 3D representation. This representation was generated with LigandScout 4.5 (2025), Inte:Ligand GmbH, Vienna, Austria (www.inteligand.com/ligandscout).

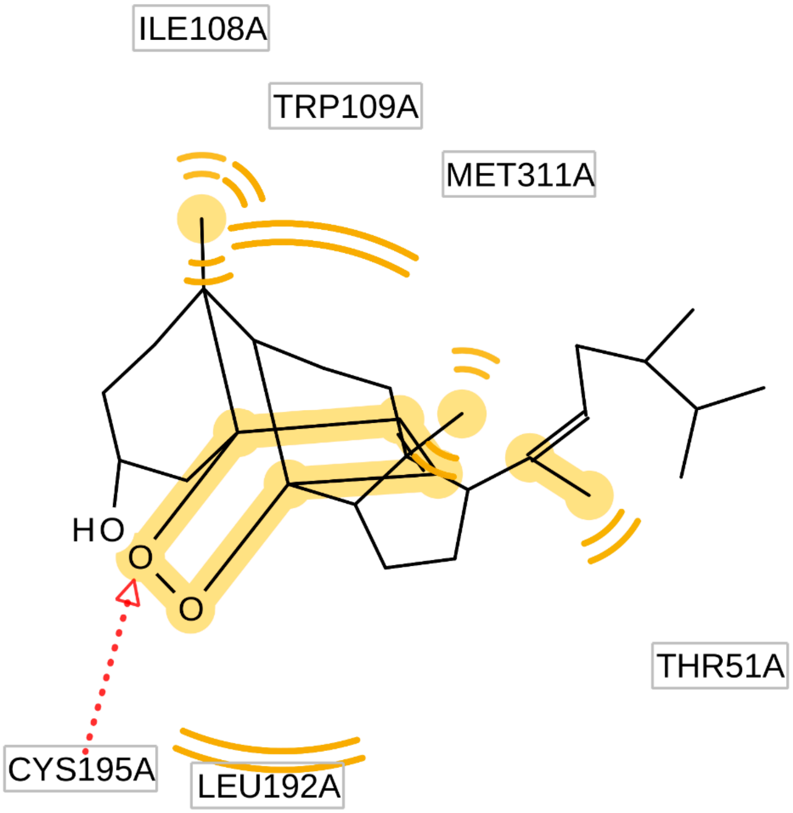

Figure S42. Pharmacophore analysis of the complex TYMS and Ergosterol peroxide in a 2D representation. This representation was generated with LigandScout 4.5 (2025), Inte:Ligand GmbH, Vienna, Austria (www.inteligand.com/ligandscout).

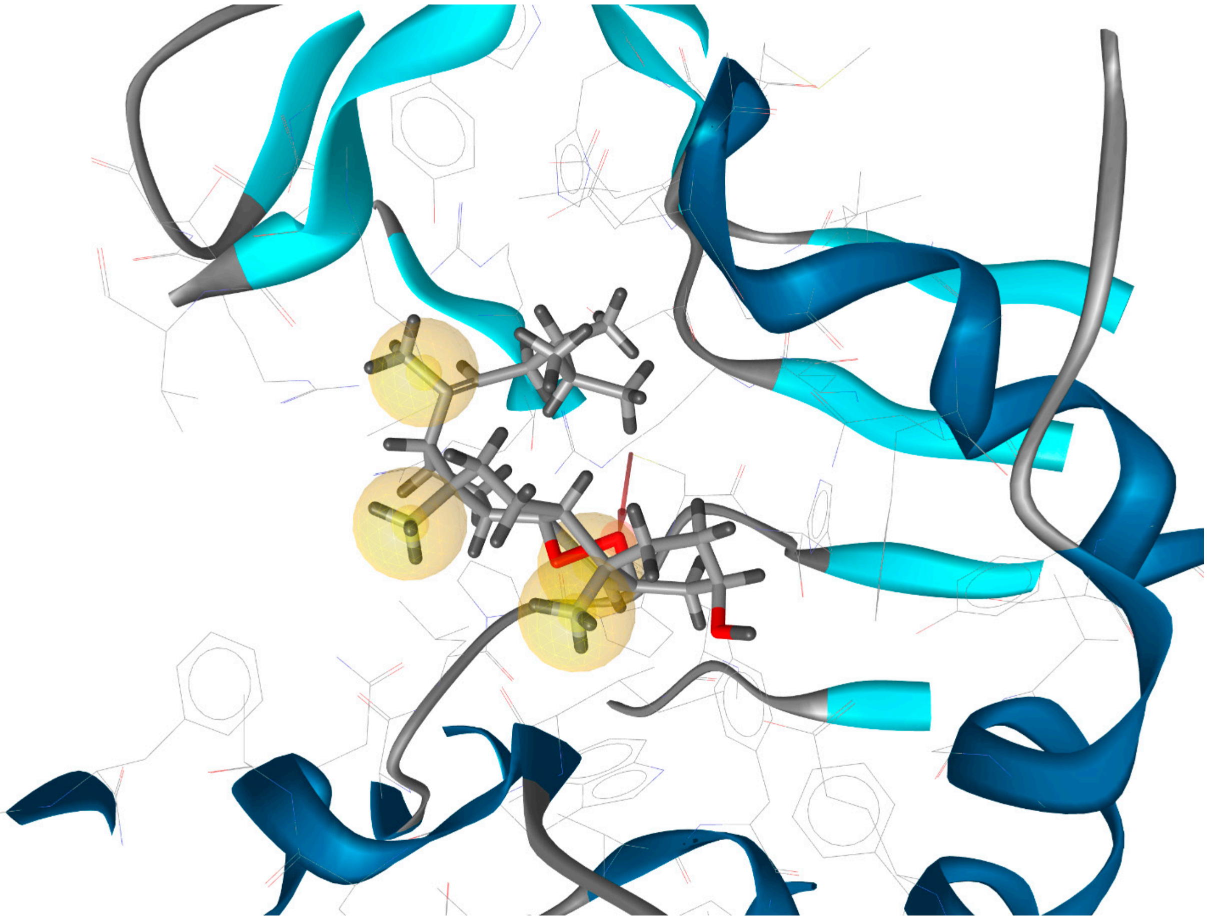

Figure S43. Pharmacophore analysis of the complex TYMS and Ergosterol peroxide in a 3D representation. This representation was generated with LigandScout 4.5 (2025), Inte:Ligand GmbH, Vienna, Austria (www.inteligand.com/ligandscout).

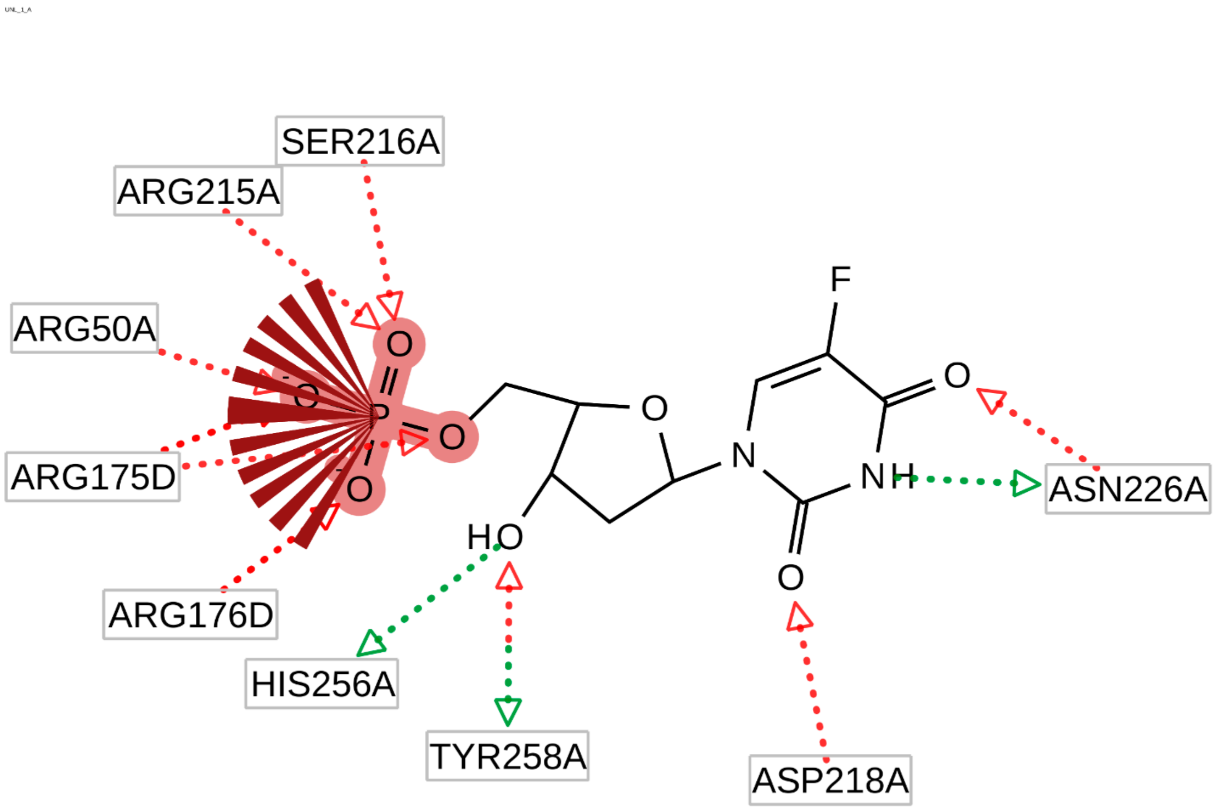

Figure S44. Pharmacophore analysis of the complex TYMS and FdUMP in a 2D representation. This representation was generated with LigandScout 4.5 (2025), Inte:Ligand GmbH, Vienna, Austria (www.inteligand.com/ligandscout).

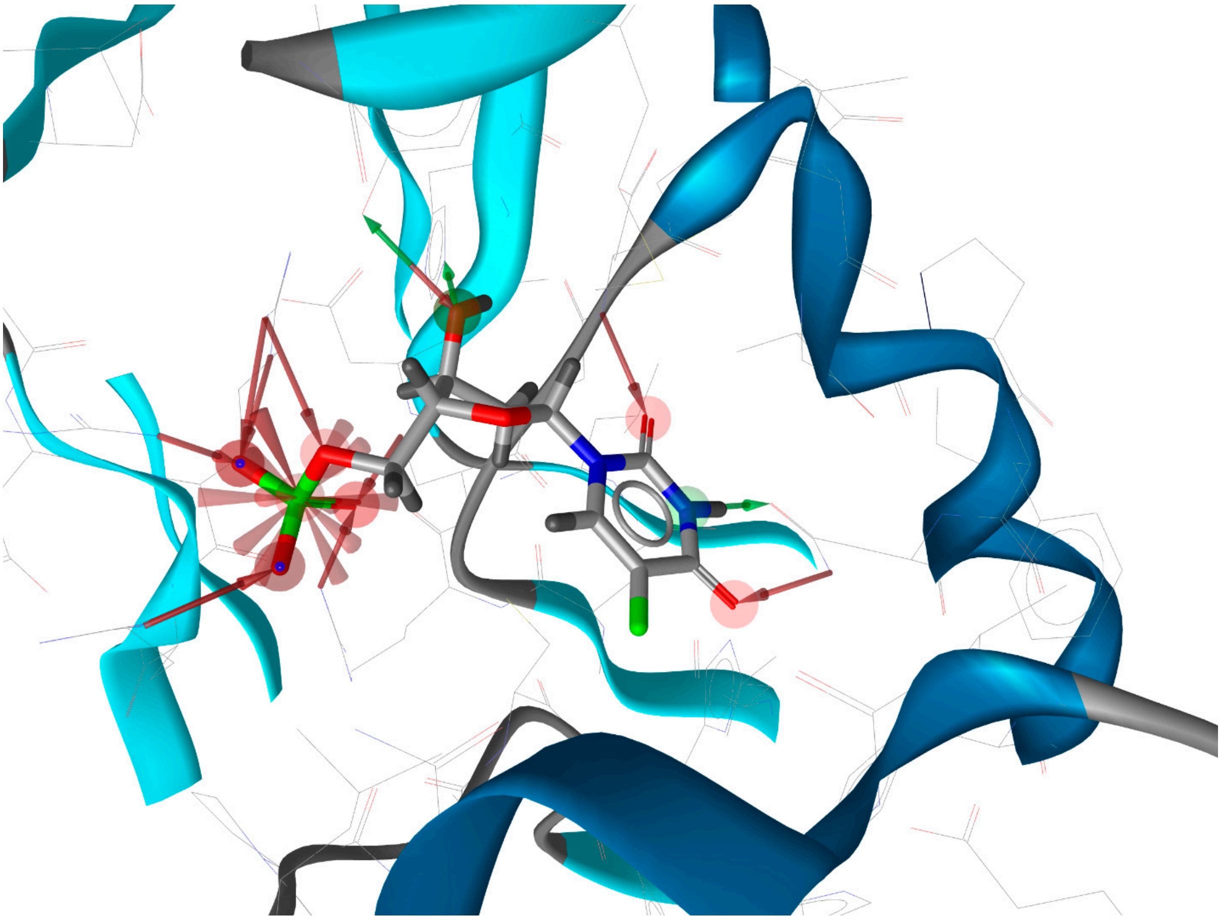

Figure S45. Pharmacophore analysis of the complex TYMS and FdUMP in a 3D representation. This representation was generated with LigandScout 4.5 (2025), Inte:Ligand GmbH, Vienna, Austria (www.inteligand.com/ligandscout).

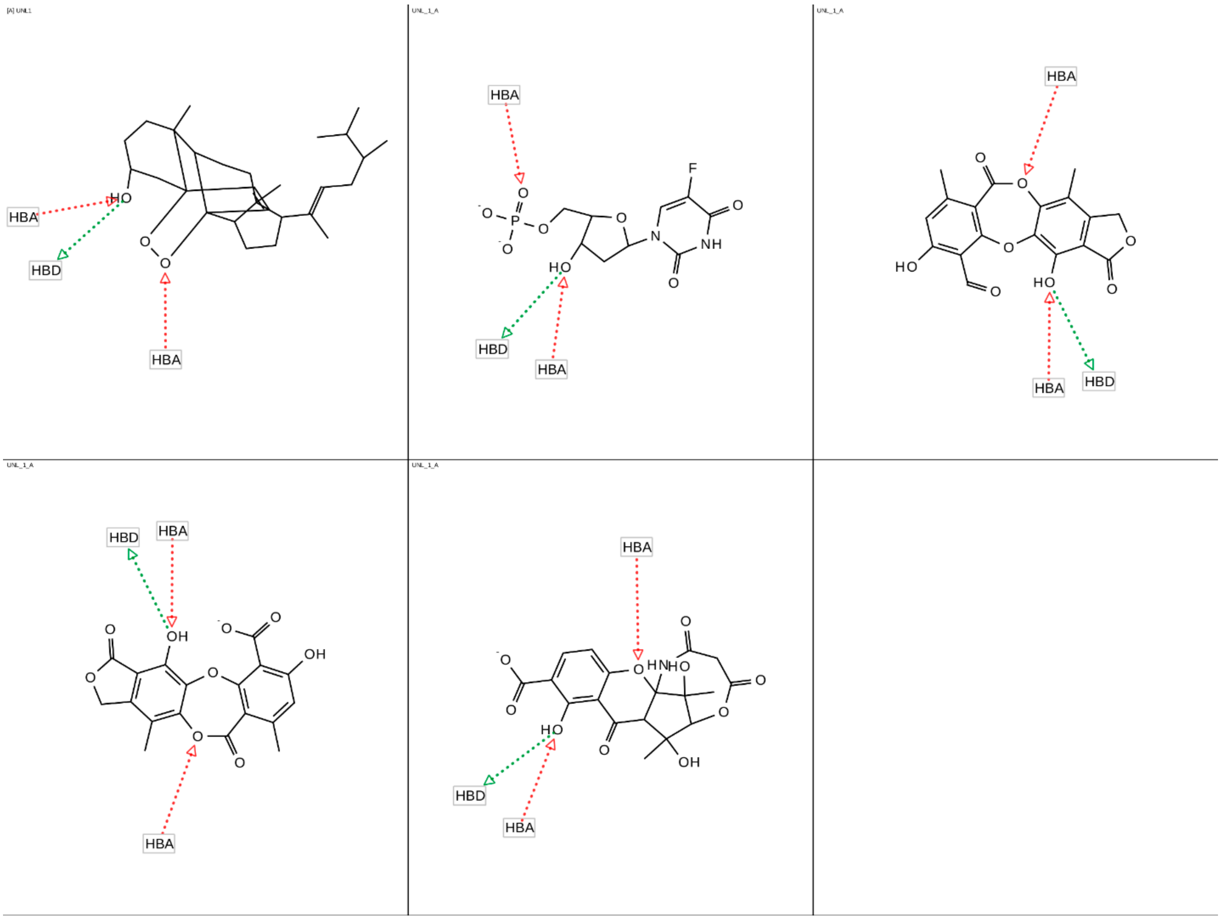

Figure S46. Alignment analysis that shows the shared pharmacophore elements of Ergosterol peroxide, FdUMP, Cordycepsidone A, Cordycepsidone B, and Cryptosporioptide A in a 2D representation. This representation was generated with LigandScout 4.5 (2025), Inte:Ligand GmbH, Vienna, Austria (www.inteligand.com/ligandscout).

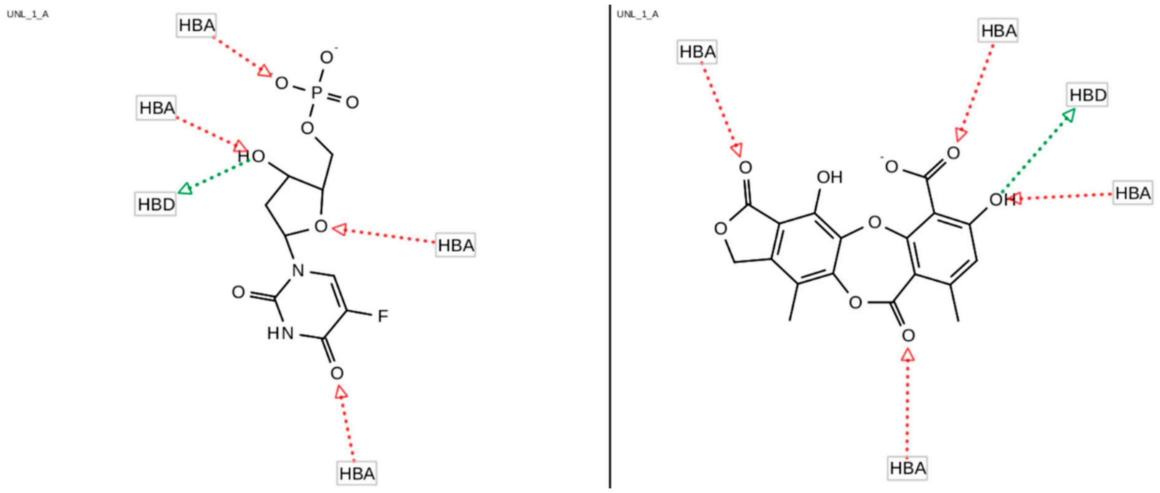

Figure S47. Alignment analysis that shows the shared pharmacophore elements of FdUMP and Cordycepsidone B in a 2D representation. This representation was generated with LigandScout 4.5 (2025), Inte:Ligand GmbH, Vienna, Austria (www.inteligand.com/ligandscout).
